# Unraveling Binding Mechanism and Stability of Urease Inhibitors: A QM/MM MD Study

**DOI:** 10.3390/molecules28062697

**Published:** 2023-03-16

**Authors:** Shunya Suenaga, Yu Takano, Toru Saito

**Affiliations:** 1 Faculty of Information Sciences, Hiroshima City University, 3-4-1 Ozuka-Higashi, Asa-Minami-Ku, Hiroshima 731-3194, Japan; 2 Graduate School of Information Sciences, Hiroshima City University, 3-4-1 Ozuka-Higashi, Asa-Minami-Ku, Hiroshima 731-3194, Japan

**Keywords:** urease, inhibitor, hydrolysis, QM/MM MD, metadynamics

## Abstract

Soil bacteria can produce urease, which catalyzes the hydrolysis of urea to ammonia (NH_3_) and carbamate. A variety of urease inhibitors have been proposed to reduce NH_3_ volatilization by interfering with the urease activity. We report a quantum mechanics/molecular mechanics molecular dynamics (QM/MM MD) study on the mechanism employed for the inhibition of urease by three representative competitive inhibitors; namely, acetohydroxamic acid (AHA), hydroxyurea (HU), and *N*-(*n*-butyl)phosphorictriamide (NBPTO). The possible connections between the structural and thermodynamical properties and the experimentally observed inhibition efficiency were evaluated and characterized. We demonstrate that the binding affinity decreases in the order NBPTO >> AHA > HU in terms of the computed activation and reaction free energies. This trend also indicates that NBPTO shows the highest inhibitory activity and the lowest IC_50_ value of 2.1 nM, followed by AHA (42 μM) and HU (100 μM). It was also found that the X=O moiety (X = carbon or phosphorous) plays a crucial role in the inhibitor binding process. These findings not only elucidate why the potent urease inhibitors are effective but also have implications for the design of new inhibitors.

## 1. Introduction

**Scheme 1:**
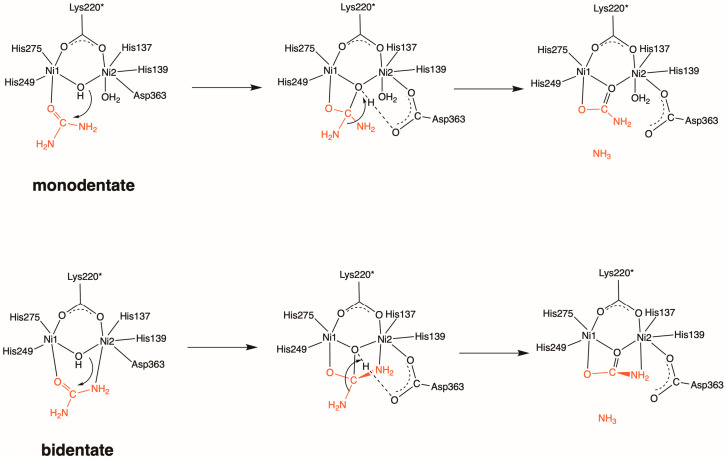
Proposed reaction mechanisms for urease-catalyzed urea hydrolysis starting from monodentate (**top**) and bidentate (**bottom**) urea–urease complexes.

Increasing food demand resulting from the growth in the world’s population is a global challenge that requires more environmentally friendly fertilizers. Nitrogen (N) fertilizers—and, in particular, urea-based fertilizers—have widely been used in agriculture [1]. In soil, N losses from urea-based fertilizers occur due to biological degradation. Soil bacteria can produce urease, a dinickel containing metalloprotein, which catalyzes the hydrolysis of urea to generate ammonia (NH_3_) and carbamate [2]. The active site of urease contains two divalent nickel ions (Ni1 and Ni2), which are bridged by a hydroxide ion and a carbamylated lysine denoted as Lys220*. His249, His275, and one water molecule (W1) are coordinated to Ni1, while His137, His139, Asp363, and one water molecule (W2) are bound to Ni2. As urea approaches the active site, its carbonyl oxygen atom binds to Ni1 in place of W1. Whether an amine N atom of urea is substituted for W2 is controversial (see Scheme 1 below). The resulting carbamate bound to the dinickel center spontaneously decomposes into NH_3_ and bicarbonate without the need for a catalyst. The NH_3_ volatilization causes 70% N losses and, thus, reduces the efficiency of the N fertilizers [3]. In addition, the functioning of urease also affects human health [4]. The emission of NH_3_ into the atmosphere may be responsible for the formation of particulate matter air pollution [5]. The World Health Organization reported that *Helicobacter pylori* (*H. pylori*) infection is a major carcinogen contributing to gastric cancer [6,7,8]. The active site of *H. pylori* urease is the same as that for the abovementioned bacterial and jack bean ureases. It neutralizes the gastric acid by generating NH_3_ so that the pathogen can colonize the human stomach. 

To interfere with the urea hydrolysis reaction catalyzed by urease, a variety of both competitive and non-competitive urease inhibitors have been developed, including hydroxyurea (HU), thiourea, acetohydroxamic acid (AHA), phosporamidates, quinones, and Au(III) compounds, to name just a few [9,10,11,12,13,14,15,16,17,18,19,20,21,22,23,24,25]. Since these currently used inhibitors are either toxic or inefficient [18], the rational design of highly effective competitive and non-competitive urease inhibitors is desirable. There have been reports predicting the binding affinity of potential inhibitors with the aid of computational tools. The methods used for this purpose include molecular docking and classical molecular dynamics (MD) simulations [21,22,23,24,25]. The most straightforward way is to elucidate the catalytic mechanism for urease, focusing on the determination of the urea-binding process and the subsequent hydrolysis reaction. Experimental studies have shown that urea binds to the active site either in a monodentate or in a bidentate manner; hence, two major reaction mechanisms have been proposed (Scheme 1) [14,26,27]. On the computational side, although density functional theory (DFT) calculations can be expected to be useful for quantitative prediction of barrier heights and reaction energies, previous DFT studies predicted similar activation barriers and failed to determine which mechanism would be more probable [28,29,30]. 

Very recently, we aimed at resolving a long-standing controversy by taking the protein environment into account. In contrast to the conventional quantum mechanics (QM) calculations with an active-site model, the quantum mechanics/molecular mechanics molecular dynamics (QM/MM MD) simulations clearly indicated that the reaction via the bidentate complex is much more favorable than that via the monodentate complex in terms of computed activation free energies (4.0 vs. 23.1 kcal/mol). We revealed that the switch in binding mode from monodentate to bidentate can facilitate the stabilization of the transition states and intermediates by forming multiple hydrogen bonds with certain active site residues [31]. Our findings support the recent observation of the X-ray structure of a urea-bound bidentate complex reported by Mazzei and co-workers [18]. 

Encouraged by the success in deciphering the urease-catalyzed hydrolysis reaction, we applied the QM/MM MD free energy simulations to the evaluation of the mechanism employed for the inhibition of urease by three representative competitive inhibitors; namely, AHA, HU, and *N*-(*n*-butyl)phosphorictriamide (NBPTO). AHA and HU are classified as substrate analog inhibitors, and NBPTO functions as a transition-state analog inhibitor. The quantitative data obtained in the present study will have implications for the design of new potent inhibitors, making it possible to rationalize the relationship between structural and thermodynamical properties and experimentally observed inhibition efficiency.

## 2. Results and Discussion

### 2.1. Inhibition by AHA *(**1**)*

**Scheme 2:**
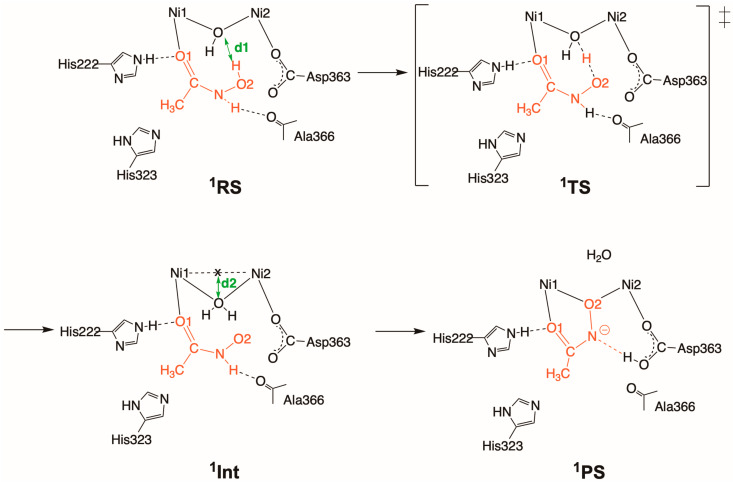
Reaction mechanisms for AHA binding to the dinickel center, starting with the AHA-bound complex (**^1^RS**) and followed by **^1^TS**, **^1^Int**, and the AHA-inhibited complex (**^1^PS**). The active-site residues of the QM region that may interact with AHA (His222, His323, Ala366) are also shown. Reaction coordinates (interatomic distances d1 and d2) are defined as collective variables.

AHA is the most widely studied competitive inhibitor of urease among hydroxamic acid derivatives [10,11,12,13,15]. While it serves as a standard reference with an IC_50_ value of ca. 42 μM, its mechanism of binding to the dinickel center is still unclear. The X-ray structure of AHA-inhibited urease suggests that the carbonyl oxygen atom of AHA (O1) is initially coordinated to Ni1 to yield an AHA-bound complex, and several chemical steps lead to the AHA-inhibited complex with the other oxygen atom (O2) bridging two nickel ions [15]. The Ni1–O2 and Ni2–O2 bond lengths are 1.95 and 2.01 Å, respectively (see also **^1^PS** in Scheme 2 below). Note that we refer to the initial complex as the AHA-bound complex to distinguish it from the AHA-inhibited complex.

**Figure 1:**
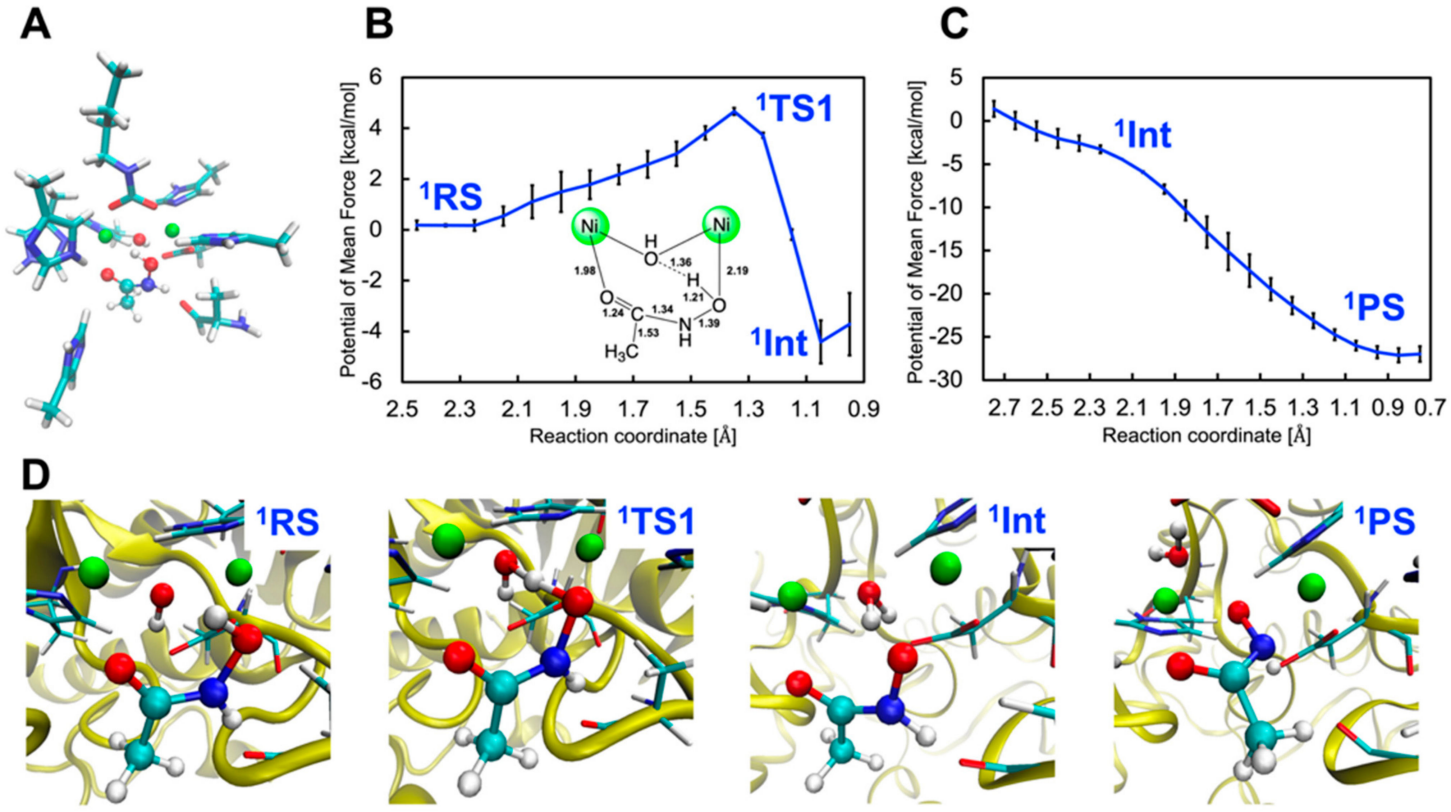
(**A**) QM region of the AHA-bound complex (**^1^RS**) obtained with the QM/MM MD equilibration; (**B**) one-dimensional potentials of mean force (1D-PMFs) for the proton transfer from AHA to WB, with an illustration of **^1^TS** (key distances are provided in Å); (**C**) 1D-PMFs for AHA-inhibited complex formation; and (**D**) representative snapshots of the active sites in **^1^RS**, **^1^TS**, **^1^Int**, and **^1^PS**.

Looking at the QM region after the QM/MM MD equilibration illustrated in Figure 1A, **^1^RS** has a monodentate binding structure with a coordination bond between the Ni1 and O1 atoms, which is in line with the experimental prediction. The reaction mechanism determined by the QM(GFN2-xTB)/MM(CHARMM36) metadynamics simulations is illustrated in Scheme 2. 

This indicates that the proton of the OH group of AHA is transferred to the bridging OH (WB) to generate an intermediate with a bridging H_2_O (**^1^Int**) via **^1^TS**. In this process whereby (**^1^RS** → **^1^TS** → **^1^Int**), the O2 atom may be weakly coordinated with Ni2 with a Ni2–O2 bond of 2.34 ± 0.17 Å. Subsequently, the bridging H_2_O molecule is replaced with O2 to yield the AHA-inhibited complex (**^1^P**), and during this process the proton bound to N is transferred to the carboxylate group of Asp363. 

To undertake a comparison with the QM/MM metadynamics results, we also conducted QM-only calculations at the UB3LYP-D3BJ/def2-SVP level of theory (for details, see Section 3.3 below). Being different from the GFN2-xTB/CHARMM36 MD equilibration, the QM-only cluster model was found to prefer another AHA-bound complex—in which AHA chelates Ni1 and its OH group forms a hydrogen bond with WB (**^1^RS_QMa_**)—over the substrate-like binding mode (**^1^RS_QMb_**), as depicted in Figure S1 from Supplementary Materials. It should be emphasized that the local coordination environments of both **^1^RS_QMa_** and **^1^RS_QMb_** are disrupted, presumably due to artificial stabilization arising from the protein environment not being included. Further arguments providing careful validation of the QM-only cluster model and exchange correlation functionals are out of the scope of the present study, but we discuss the results of the QM-only calculations in the Supplementary Material, as exemplified with a possible reaction mechanism starting from **^1^RS_QMa_** (Figure S2 from Supplementary Materials). The same is true for the QM-only results for the inhibition process of HU (Figures S3 and S4 from Supplementary Materials).

Figure 1B,C show the one-dimensional potentials of mean force (1D-PMFs) for the first reaction step along the H(AHA)•••O(WB) separation, ranging from 0.90 to 2.50 Å (denoted as CV d1), and the second one along the distance between O2 and the center-of-mass position of Ni1 and Ni2, ranging from 0.70 to 2.50 Å (denoted as CV d2), respectively. Representative snapshots of the key states are shown in Figure 1D. The computed free energy barrier at **^1^TS** was 4.7 ± 0.1 kcal/mol, which is slightly higher than that corresponding to the nucleophilic attack of WB on urea (4.0 kcal/mol) [31]. As can be seen in Scheme 2 and Figure 1B, the longer O•••H and shorter O2•••H distances suggest an early transition state, and the switch in the binding mode from monodentate to bidentate can occur at **^1^TS**. The formation of **^1^Int** is exergonic, with a value of –4.4 ± 0.9 kcal/mol relative to **^1^RS**. To our surprise, all three metadynamics simulations yielded 1D-PMFs with no barriers between **^1^Int** and **^1^P**, indicating that the second substitution step takes place spontaneously or that the free energy landscape in the vicinity of **^1^Int** takes the form of a plateau or shoulder. This might be driven by the hydrogen-bonding interactions with nearby residues and positive entropic contributions [32,33]. The reaction free energy of –27.1 ± 0.8 kcal/mol was more exergonic compared to that calculated for the NH_3_ and carbamate formation catalyzed by urease (–22.5 kcal/mol) [31]. 

As in the case of the urease-catalyzed urea hydrolysis reaction, multiple hydrogen bonds with adjacent residues stabilize **^1^TS**, **^1^Int**, and **^1^P** (Scheme 2). Stable hydrogen-bonding interactions between AHA and His222 remain throughout the binding processes, with O2(AHA)•••Nε(His222) separations of 3.01 ± 0.29 Å. AHA also forms a hydrogen bond with Ala366 before the proton migration occurs from AHA to Asp363. The N(AHA)•••O(Ala366) distance of 2.89 ± 0.15 Å in the first step becomes elusive, with an increased distance of 3.43 ± 0.40 Å in the second one. In **^1^P**, the hydrogen-bond interaction between AHA and Asp363 seems not to be rigid in terms of the N(AHA)•••O(Asp363) separation of 3.27 ± 0.33 Å (Figure S5 from Supplementary Materials). 

### 2.2. Inhibition by HU (***2*** and ***2′***)

#### 2.2.1. Binding Mode 2

Urea derivatives may be candidates as substrate analogs that show inhibitory activity [9,20]. Replacement of one of the NH_2_ groups of urea with the NHOH group leads to HU, which can inhibit the activity of urease with an IC_50_ value of ca. 100 μM and has also been used as a reference inhibitor [20,24]. On the other hand, semicarbazide, which has the NHNH_2_ group instead, does not function as an inhibitor but rather undergoes hydroxylation by urease [34]. The mechanisms for the binding and inhibition of HU are still unknown, and no X-ray structures for HU-inhibited complexes have been reported so far. The kinetic analysis of urease hydrolysis in the presence of HU demonstrated that the reaction exhibits biphasic kinetics starting with a rapid burst phase, which is followed by a slow plateau phase due to inhibition by HU [9,10,33]. 

**Scheme 3:**
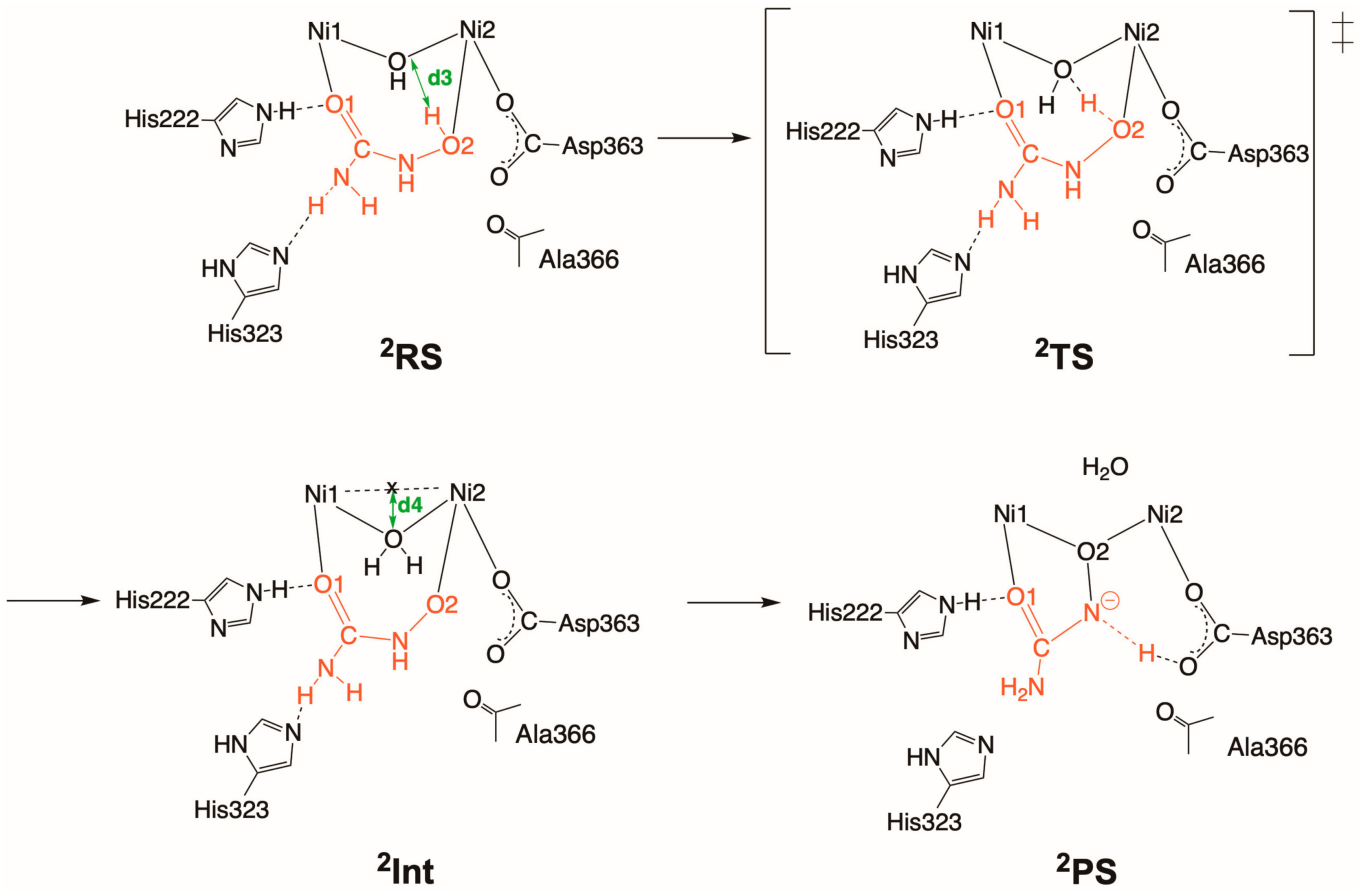
Reaction mechanisms for HU (**2**) binding to the dinickel center, starting with the HU-bound complex (**^2^RS**) and followed by **^2^TS**, **^2^Int**, and the HU-inhibited complex (**^2^PS**). The active-site residues of the QM region that may interact with HU (His222, His323, Ala366) are also shown. Reaction coordinates (interatomic distances d3 and d4) are defined as collective variables.

**Scheme 4:**
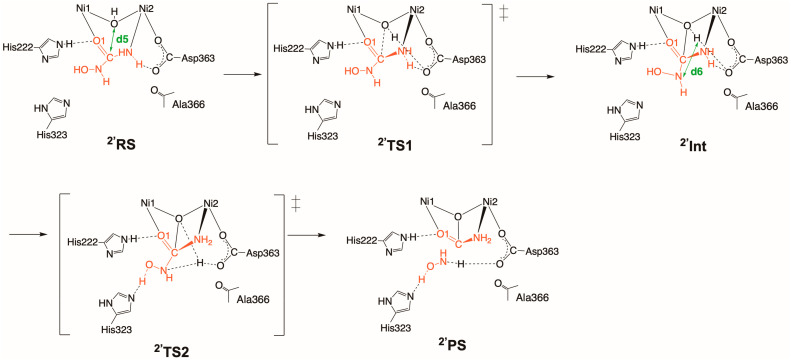
Reaction mechanisms for HU (**2′**) binding to the dinickel center, starting with the HU-bound complex (**^2′^RS**) and followed by **^2′^TS1**, **^2′^Int, ^2′^TS2,** and the HU-inhibited complex (**^2′^PS**). The active-site residues of the QM region that may interact with HU (His222, His323, Ala366) are also shown. Reaction coordinates (interatomic distances d5 and d6) are defined as collective variables.

**Figure 2:**
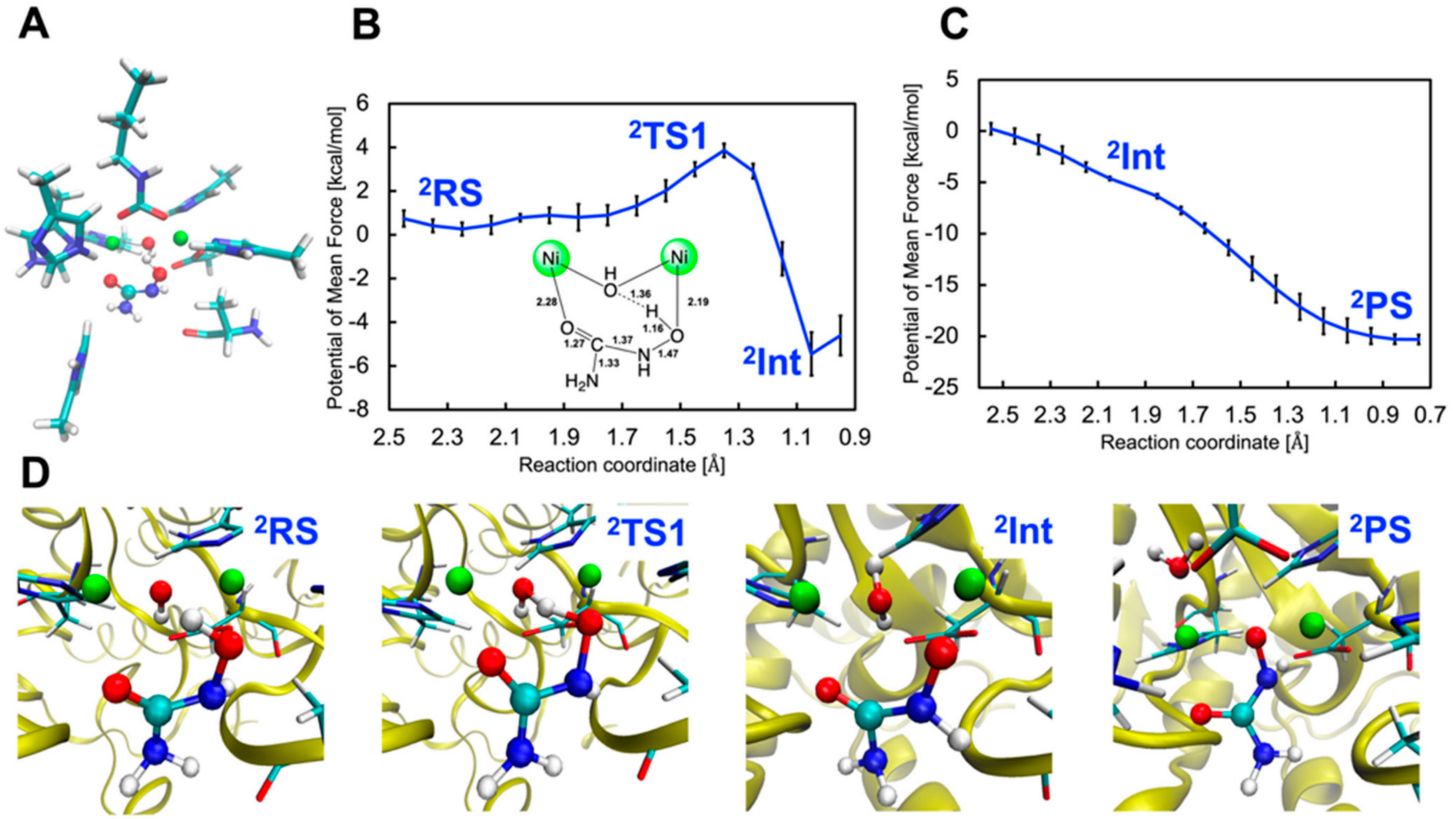
(**A**) QM region of HU(**2**)-bound complex (**^2^RS**) obtained with the QM/MM MD equilibration; (**B**) one-dimensional potentials of mean force (1D-PMFs) for the proton transfer from HU to WB, with an illustration of **^2^TS** (key distances are provided in Å); (**C**) 1D-PMFs for AHA-inhibited complex formation; and (**D**) representative snapshots of the active sites in **^2^RS**, **^2^TS**, **^2^Int**, and **^2^PS**.

This means that HU might act simultaneously as a substrate and irreversible inhibitor of urease. In light of these previous experimental studies, we examined HU-bound complexes in two different binding modes (**2** and **2′**; see also Scheme 3 and Scheme 4). In the remainder of this section, we discuss the results starting with **2**. As can be seen in Scheme 3 and Figure 2A, the HU-bound complex (**^2^RS**) obtained from the QM/MM MD equilibration step exhibits a weak bidentate coordination, with Ni1-O1 and Ni2-O2 bond lengths of 2.25 and 2.34 Å. The Ni1-coordinated O1 atom of HU forms a hydrogen bond with His222, as in the case of AHA. Since HU differs from AHA in that it has the NH_2_ group in place of the CH_3_ group, a hydrogen bond between the NH_2_ group and His323 could also be observed in **^2^RS**. The attempt failed to elucidate the hydrolysis of HU departing from **^2^RS** that would yield NH_3_, despite several trials using different QM/MM MD equilibrated snapshots as the initial geometry. Instead of pursuing the nucleophilic attack of WB, we considered that a dead-end complex may be formed; namely, the HU-inhibited complex, which resembles the AHA-inhibited complex.

Figure 2B displays the 1D-PMFs along the reaction coordinate set to the H(HU)•••O(WB) separation in the range from 0.90 to 2.50 Å (denoted as CV d3). We were able to locate suitable transition state (**^2^TS**) and intermediate (**^2^Int**) configurations. The structural features of **^2^TS** closely resemble those of **^1^TS**, with an elongated O–H bond-forming distance of 1.36 Å. The activation free energy at **^2^TS** of 3.9 ± 0.3 kcal/mol is slightly lower than the value estimated for AHA of 4.7 ± 0.1 kcal/mol. The formation of **^2^Int** shows a slightly larger exergonicity compared to the reaction for AHA (–5.5 ± 1.0 vs. –4.4 ± 0.9 kcal/mol).

Then, the 1D-PMFs corresponding to the release of a new bridging water molecule were estimated along the distance between O2 and the center-of-mass position of Ni1 and Ni2, ranging from 0.70 to 2.50 Å (denoted as CV d4). Figure 2C shows that the formation of the HU-inhibited complex (**^2^P**) via chelation through O1 and O2 atoms is barrier-free, similar to the **^1^P** production. Likewise, the proton binding to N is transferred to the carboxylate group of Asp363, forming a weak hydrogen bond N(HU)•••H(Asp363) = 3.02 ± 0.29 Å (Figure 2D and Figure S6). The hydrogen-bond distance of 3.02 ± 0.29 Å between O1(HU) and Nε(His222) is much shorter than that of 3.41 ± 0.37 Å between N(HU) and Nδ(His323). The hydrogen-bond interactions with His323 gradually disappear, showing a N(HU)•••Nδ(His323) separation of 4.21 ± 0.54 Å, while those with His222 become less rigid judging from the O(HU)•••Nε(His222) of 3.23 ± 0.40 Å. 

#### 2.2.2. Binding Mode ***2′***

**Figure 3:**
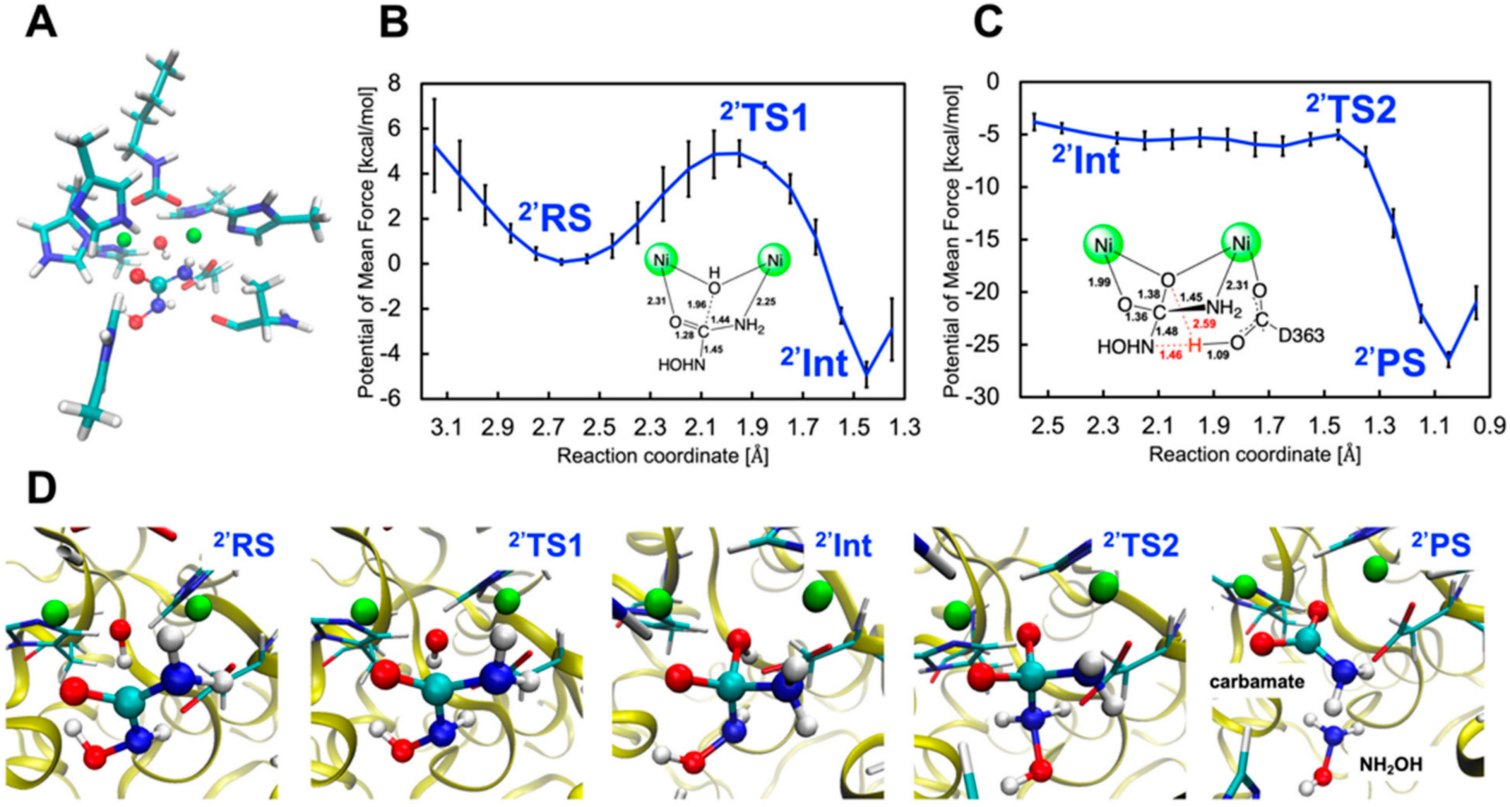
(**A**) QM region of HU(**2′**)-bound complex (**^2^RS**) obtained with the QM/MM MD equilibration; (**B**) one-dimensional potentials of mean force (1D-PMFs) for the nucleophilic addition step, with an illustration of **^2′^TS1** (key distances are provided in Å); (**C**) 1D-PMFs for the release of NH_2_OH and HU-inhibited complex formation, with an illustration of **^2′^TS2** (key distances are provided in Å); and (**D**) representative snapshots of the active site in **^2′^RS**, **^2′^TS1**, **^2′^Int**, **^2′^TS2,** and **^2^PS**.

Let us look into to the other binding mode **2′**. In the reactant state (**^2′^RS**), HU seems to form a weak bidentate complex, as well as **^2^RS**, with Ni1–O1 and Ni2–N distances of 2.34 and 2.41 Å (Scheme 4 and Figure 3A). Our QM(GFN2-xTB)/MM(CHARMM36) metadynamics calculations demonstrated that **^2′^RS** triggers the hydrolysis of HU by using the C(HU)•••O(WB) separation ranging from 1.30 to 3.20 Å as the reaction coordinate (denoted as CV d5). As shown in Scheme 4, the formation of a tetrahedral intermediate (**^2′^Int**) proceeds through the nucleophilic attack of WB on the carbonyl carbon atom via **^2′^TS1**.

The C–O bond-forming distance of 1.96 Å, as well as the shortened Ni1–O1 and Ni2–N distances in **^2′^TS1,** matches well with the result obtained for the urea hydrolysis (Figure 3B) [31]. The strong hydrogen bonding between O1(HU) and Nε(His222) of 3.08 ± 0.26 Å plays a key role in enhancing inhibitor binding. The NHOH moiety of HU may form weak and fluctuating hydrogen bonds with His323 and Asp363 during the nucleophilic attack step, as the O(HU)•••Nδ(His323) and N(HU)•••O(Asp363) bond separations are 3.18 ± 0.46 and 3.15 ± 0.44 Å, respectively (see Scheme 4). Figure 3B highlights that the free energy barrier of 4.9 ± 0.6 kcal/mol at **^2′^TS** is higher in energy than that at **^2^TS** discussed above, and the reaction free energy of −4.9 ± 0.6 kcal/mol at **^2′^Int** with respect to **^2′^RS** is less exergonic compared to the result for **2** (−5.5 ± 1.0 kcal/mol).

Upon the formation of **^2′^Int**, we examined the elimination process caused by proton transfer from WB to the departing NHOH group of HU based on the reaction coordinate set for the H(WB)•••N(HU) separation in the range from 0.90 to 2.70 Å (denoted as CV d6). All three of the QM/MM metadynamics simulations demonstrated the same reaction mechanism involving Asp363-assisted proton transfer, as illustrated in Figure 3C. The proton bound to Asp363 in **^2′^TS2** interconnects between **^2′^Int** and the product state **^2′^P**, where the product carbamate is tridentately bound to the nickel center and NH_2_OH is released (Figure 3D). It is obvious that this step is highly exergonic, with **^2′^TS2** and **^2′^P** presenting free energies of −5.0 ± 0.5 and −26.4 ± 0.7 kcal/mol relative to **^2′^RS**. During the second reaction step, the hydrogen-bond distances between O1(HU) and Nε(His222) and N(HU) and Nδ(His323) are 2.88 ± 0.15 and 2.99 ± 0.35 Å, respectively. As shown by the analysis of the trajectories, the proton can move back and forth between the isolated hydroxylamine group and the carboxylate group of Asp363, existing as either NH_2_OH and the deprotonated Asp363 or NHOH^–^ and the protonated Asp363 (Figure S7 from Supplementary Materials). This behavior was not observed in the simulations of urea hydrolysis. 

Unlike the results for **1** and **2**, the UB3LYP-D3BJ geometry optimization favored a bidentate HU-bound complex (**^2′^RS_QMa_**), as shown in Figure S9 from Supplementary Materials. It resembles the GFN2-xTB-optimized reactant complex (**^2′^RS_QMb_**). At the QM-only level, the hydrolysis of hydroxyurea was found to proceed similarly to that of urea, in that the elimination processes involving proton transfer followed by C–N bond cleavage require larger activation barriers than the nucleophilic attack process [31]. The activation potential energies are 10.8 and 5.2 kcal/mol for the former process and 18.1 and 8.4 kcal/mol for the latter process, as calculated at the UB3LYP-D3BJ and GFN2-xTB levels of theory. The comparison between the QM/MM metadynamics simulations and QM-only calculations clearly indicates the importance of the protein environment. The explicit inclusion of the protein environment is crucial for lowering the activation barrier for the elimination steps because it might offer the benefits of hydrogen-bonding interactions with nearby residues and a positive entropic contribution [32].

Comparison of the results for **2** and **2′** suggested that these reactions are competitive and, because of this, HU might function simultaneously as a substrate and irreversible inhibitor, which is in good agreement with the experimental results [9,10]. The reaction of urease with **2** is involved in the generation of the dead-end complex **^2^P**, where an HU-derived compound is irreversibly bound to the active site by chelating Ni1. On the other hand, in the other binding mode **2′**, HU was found to undergo hydrolysis catalyzed by urease. Since NH_2_OH produced by hydrolysis has no inhibitory activity towards urease, the generated carbamate in **^2′^P** is supposed to be decomposed into NH_3_ and bicarbonate, as can be observed in urea hydrolysis. Although the amount of NH_3_ arising from HU hydrolysis is half of that arising from urea hydrolysis, the inhibition activity of HU is inferior to that of AHA, and this reflects the difference in the IC_50_ values (42 vs. 100 μM).

### 2.3. Inhibition by NBPTO *(**3**)*

Finally, the inhibition process for NBPTO was explored. *N*-(*n*-butyl) thiophosphoric triamide (NBPT), which is the most well-known phosporamidate-type urease inhibitor in agriculture, is a pro-drug of NBPTO. Analogous to other phosporamidate derivatives, NBPTO works as a suicide substrate and shows a substantially low IC_50_ value of 2.1 nM. When NBPTO approaches the active site, urease mistakenly classifies it as a true substrate and hydrolyzes it to diamido phosphoric acid (DAP) and *n*-butylamine instead of releasing NH_3_. The resulting transition-state analog DAP is strongly bound to the active site of urease in a tridentate manner; thus, the binding of another urea molecule can be blocked. We investigated the relationship between the high inhibitory activity of NBPTO and DAP formation catalyzed by urease. Specifically, we assessed whether NBPTO hydrolysis is essentially the same as urea hydrolysis or whether it is subject to a different reaction pathway.

**Scheme 5:**
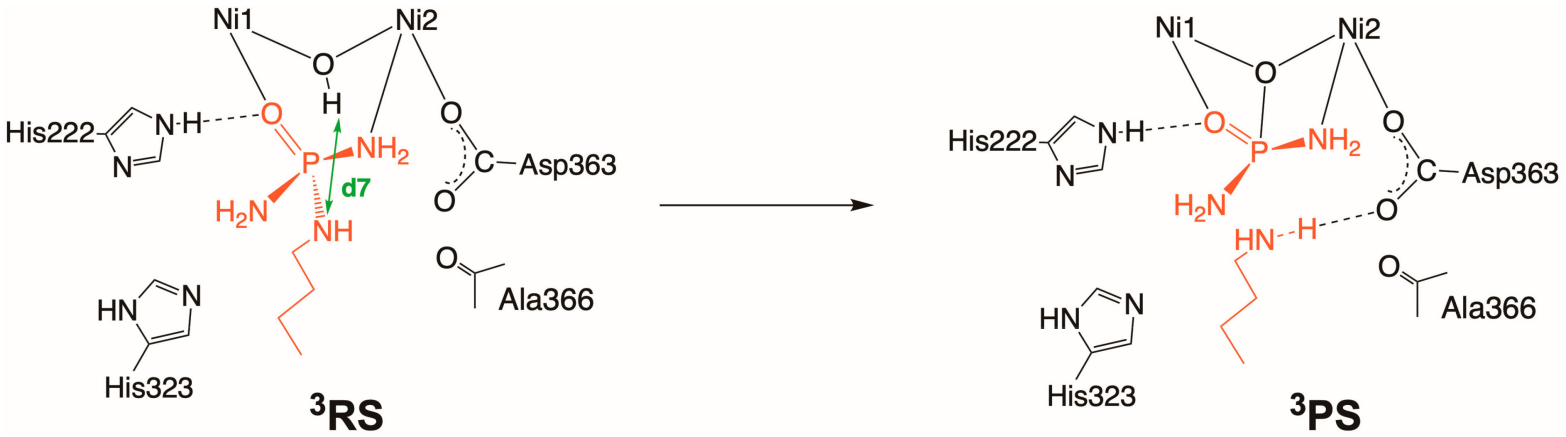
Reaction mechanisms for NBPTO binding to the dinickel center via urease-catalyzed hydrolysis reaction, starting with the NBPTO-bound complex (**^3^RS**) and followed by the DAP-inhibited complex (**^3^PS**). The active-site residues of the QM region that may interact with NBPTO (His222, His323, Ala366) are also shown. The reaction coordinate (interatomic distance d7) is defined as a collective variable.

**Figure 4:**
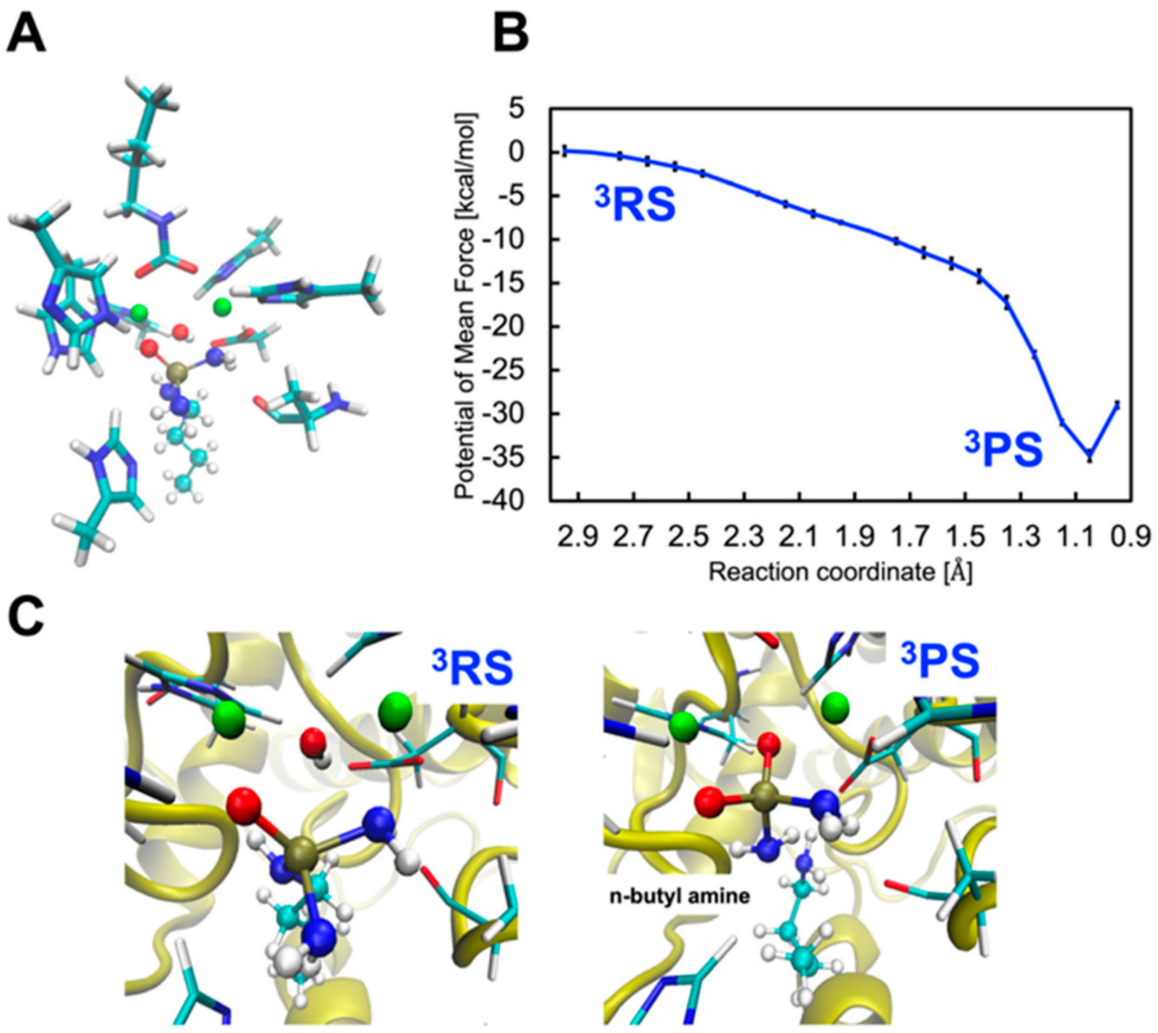
(**A**) QM region of NBPTO-bound complex (**^3^RS**) obtained with the QM/MM MD equilibration; (**B**) one-dimensional potentials of mean force (1D-PMFs) for the release of *n*-butylamine and the DAP-inhibited complex formation step; and (**C**) representative snapshots of the active sites in **^3^RS** and **^3^PS**.

Following the QM/MM MD equilibration procedure, the NBPTO-bound complex (**^3^RS**) was obtained (Scheme 5). It is characterized by a bidentate complex that has Ni1–O and Ni2–O bonds of 2.22 and 2.13 Å, and the distance between O(WB) and P(NBPTO) is 2.69 Å (Figure 4A).

We first attempted to track the formation of a tetrahedral intermediate that would be the precursor of DAP, with the reaction coordinate set to the O(WB)•••P(NBPTO) separation ranging from 1.40 to 3.20 Å (denoted as CV d7). However, all metadynamics simulations failed to determine an appropriate free energy profile for the nucleophilic addition reaction. In contrast to the case of urea hydrolysis, the P–OH bond is not likely to be formed. For this reason, we predicted that P–O bond formation would coincide with P–N bond scission via an Asp363-assisted proton migration reaction from WB to the amine N atom. The H(WB)•••N(NBPTO) distance in a range of 0.90 to 2.70 Å was chosen as the reaction coordinate. Looking at the 1D-PMFs shown in Figure 4B and the corresponding trajectories, it appears that the proton binding to WB moves to the amine N atom directly and not via Asp363, and this is followed by the formation of a barrierless P–O bond and concomitant P–N bond breaking whereby DAP and *n*-butylamine (**^3^P**) are generated.

After *n*-butylamine is produced, its NH_2_ group forms a hydrogen bond with Asp363 and the proton moves back and forth between *n*-butylamine and Asp363 (Figure S8 from Supplementary Materials). The resulting **^3^P** is stable with a reaction free energy of –34.8 ± 0.7 kcal/mol. The hydrogen bond between NBPTO and His222 remains strong throughout the inhibition processes, with O(NBPTO)•••Nε(His222) separations of 2.90 ± 0.20 Å. No hydrogen-bonding interactions were detected between His323 and the amino group of NBPTO that were not coordinated with Ni2. Our data support the notion that the presence of a C=O or P=O moiety is mandatory for potent inhibition of urease, as NBPTO with a P=O bond shows much better inhibition than NBPT with no P=O bond in the region of four orders of magnitude [16].

**Figure 5:**
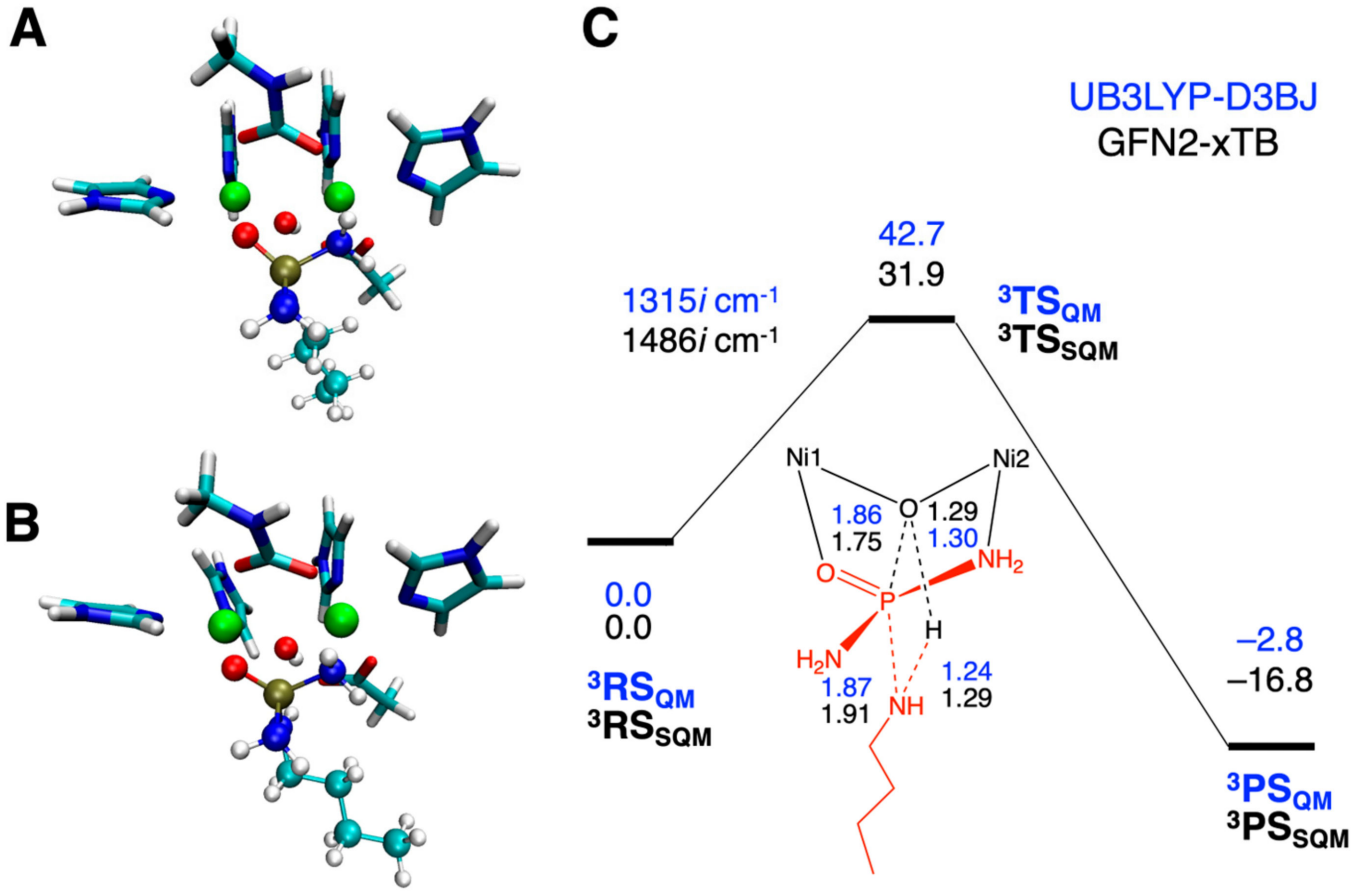
(**A**) NBPTO-bound complex optimized at the UB3LYP-D3BJ/def2-SVP level (**^3^RS_QM_**); (**B**) NBPTO-bound complex optimized at the GFN2-xTB level (**^3^RS_SQM_**); and (**C**) potential energy profiles (in kcal/mol) and illustration of the transition state structures with computed imaginary frequencies corresponding to the inhibition process for NBPTO obtained with the UB3LYP-D3BJ/def2-SVP and GFN2-xTB computations.

In contrast to the results for **1**, **2**, and **2′**, the UB3LYP-D3BJ geometry optimization demonstrated a proper bindentate NBPTO-bound complex (**^3^RS_QM_**), as shown in Figure 5A. **^3^RS_QM_** appears to be comparable to **^3^RS** (Figure 4A), as well as the GFN2-xTB-optimized complex (Figure 5B), which was additionally obtained for comparison. Then, the formation of DAP and *n*-butylamine was explored by locating stationary points. The resulting **^3^TS_QM_** and **^3^PS_QM_** suggested that the direct proton transfer mechanism was also underpinned by the QM-only results. The same trend was observed when using GFN2-xTB, leading to **^3^TS_SQM_** and **^3^PS_SQM_**. Comparison of the relative energies of the stationary points calculated using UB3LYP-D3BJ and GFN2-xTB revealed that GFN2-xTB underestimated the barrier height by 10.6 kcal/mol and overestimated the magnitude of the reaction energy by 14.0 kcal/mol. The results are qualitatively consistent with those for urea hydrolysis but totally different from those for the present GFN2-xTB/CHARMM36 metadynamics simulations (Figure 4B). Further discussion should be postponed until UDFT-based QM/MM metadynamics simulations are routinely performed.

## 3. Methods

### 3.1. Model Preparation and Classical MD Simulations

The X-ray crystal structure of fluoride-inhibited *Sporosarcina pasteurii* urease was obtained from the Protein Data Bank (PDB entry 6QDY) [18]. The fluoride bridged between the two Ni ions was substituted for a hydroxide ion, which is referred to as WB. Urea bound to the active site was replaced with AHA, HU, and NBPTO to construct inhibitor-bound complexes (see Figure 1, Figure 2, Figure 3 and Figure 4), as noted above. For each complex, the system preparation for the subsequent calculations, including classical MD simulations, was carried out in the same manner as our previous work [31]. The QM subsystem consists of the inhibitor, Ni1, Ni2, WB, and side chains of residues coordinated with two Ni ions (His137, His139, Lys220*, His249, His275, and Asp363). The protonation states of titratable residues were estimated based on the p*K*a values computed with the PROPKA3 program [35,36], and then hydrogen atoms were added with the CHARMM-GUI input generator [37]. Concerning the active-site histidine residues (His222 and His323), the epsilon position of His222 and the delta position of His323 were respectively assumed to be protonated. Previous studies recommended the spin-unrestricted B3LYP (UB3LYP) calculation as suitable for the calculation of the structural and magnetic properties of the urease active site [28,29,30,31,38,39,40,41,42]. The geometry was fully optimized using the Gaussian version of UB3LYP with D3BJ dispersion corrections [40,41,42,43,44] in conjunction with the def2-SVP basis set [45], which is referred to as UB3LYP-D3BJ/def2-SVP. The ChelpG charges [46] computed for the optimized structure were assigned to the force field partial atomic charges for Ni ions, WB, and ligating residues. These calculations were conducted with the ORCA 5.0.1 program package [47]. The Lennard-Jones parameters for Ni were taken from [48]. The CHARMM force field parameters for each inhibitor and Lys220* were generated with the CHARMM General Force Field program [49].

Subsequently, each system was immersed in a 100 × 110 × 100 Å rectangular box of water molecules and neutralized with two Na^+^ ions using the Visual Molecular Dynamics (VMD) program [50]. Classical MD simulations were performed under periodic boundary conditions in the NPT ensemble at 300 K with a time step of 2.0 fs. Long-range electrostatic interactions were treated with the particle mesh Ewald (PME) method [51] with a tolerance of 10^−6^. The electrostatic and van der Waals interactions were cut off at 14 Å with a switching distance of 12 Å. The system was minimized over 2000 steps, which was followed by a 10 ns classical MD simulation using the NAMD program [52]. During the MD simulation, the QM atoms were kept fixed, while the atoms in the MM region were represented by the CHARMM36 force fields and the TIP3P water models [53]. The resulting trajectories for all systems are presented in Figures S10–S13. The final coordinates of this simulation were used as the initial configuration for the following QM/MM MD equilibrations.

### 3.2. QM/MM MD Simulations

The QM/MM MD equilibrations were carried out with NAMD [52], ORCA [47] and the NAMD-ORCA interface [54], which manages data communication between the QM and MM results obtained by ORCA and NAMD. The QM region was treated using the semiemprical GFN2-xTB method [55,56] in accordance with our previous QM/MM MD study on urea hydrolysis catalyzed by urea [31]. GFN2-xTB was able to locate local minima and transition states, whereas the other semiempirical methods, such as the PMx series [57,58], tended to cause the collapse of the binuclear active-site conformations. This was presumably because the PMx family is not compatible with the coordination environment of the active site of urease. The total charge and spin multiplicities in the QM region were set to 1 and 5 for each system, while the MM subsystem was described using CHARMM36 force field parameters [49] in the same way as the classical MD simulation. Electrostatic interactions between QM and MM atoms were dealt with using the electrostatic embedding scheme, and the QM-MM boundary atoms were capped by link (hydrogen) atoms with the charge shift method [59]. The simulations were run for 2.0 ps with a time step of 0.5 fs.

After that, we performed three independent QM(GFN2-xTB)/MM(CHARMM36) metadynamics simulations [60] to determine the free energy landscape. The collective variables (CVs) for the metadynamics simulation were set to drive the reactions in the following manner (see also Scheme 2, Scheme 3, Scheme 4 and Scheme 5 and the main text). For the inhibition process for AHA (**1**), the first CV (d1) represented proton transfer from the hydrogen atom of AHA to the WB, and the second one (d2) indicated the dissociation of the formed H_2_O molecule through the breaking of both the Ni1–O and Ni2–O bonds. The same was applied for the CVs for the reaction involving HU (**2**) (d3 and d4). For the CVs for the hydrolysis of HU (**2’**), the first CV (d5) represented the nucleophilic attack of the bridging oxo on the carbonyl carbon atom, and the second CV (d6) indicated the elimination of the generated NH_3_. The CV for the process of inhibition by NBPTO (**3**) (d7) represented the P(NBPTO)–O(WB) binding, proton transfer, and the dissociation of *n*-butylamine, all of which occur concurrently. On these bases, 1D-PMFs for the inhibition mechanisms were evaluated in the NVT ensemble at 300 K. The time step, hill weight, hill width, and hill frequency were set to 0.5 fs, 0.40 kcal/mol, 2.5 Å, and 50 fs, respectively. The simulation time ranged from 15 to 50 ps, depending on the type of reaction and the choice of reaction coordinates, as mentioned above. One barrier recrossing over the transition state was taken as the convergence criterion, as recommended in literature studies [61,62,63]. At least one barrier recrossing was observed in the former processes (d1, d3, and d5) for each system, whereas, in the other cases (d2, d4, d6, and d7), the dissociated products—namely, H_2_O, NH_2_OH, and *n*-butylamine—moved away from the reaction site and the systems did not return to the reactant states. Thus, for the latter cases, we optimized the simulation times for each case, so that three independent runs generated consistent PMFs and the simulations were deemed to have converged.

### 3.3. QM-Only Cluster Model Approach

GFN2-xTB can rigorously retain the coordination environment of the binuclear nickel active site of urease, but one must keep in mind that it may provide significantly large errors in the range of 10–30 kcal/mol for reactions containing metals [64]. Our previous QM-only calculations on urea hydrolysis gave rise to significant deviations in relative energies between the GFN2-xTB and UB3LYP/def2-SVP results, although GFN2-xTB successfully provided the reaction mechanisms that were obtained from UB3LYP/def2-SVP [31]. As such, the possible mechanisms for **1**–**3** predicted by the GFN2-xTB/CHARMM36 MD simulations were also explored at the UB3LYP-D3BJ/def2-SVP level of theory, together with QM-only cluster models. For each system, the total charge and spin multiplicities were set to 1. The open-shell singlet solutions were generated using the FlipSpin and FinalMs keywords available in ORCA. To exclude artificial intramolecular interactions, we constructed each QM-only cluster model by removing the second sphere amino acids (His222, His323, and Ala366) and replacing Lys220* and histidines with methylcarbamic acid and imidazole (Figures S1, S3 and S9). All QM-only calculations were conducted with the ORCA program [47].

## 4. Conclusions

In the present study, we revealed the inhibition mechanism for urease employed by three representative competitive inhibitors; namely, AHA, HU, and NBPTO. The reactivity of these inhibitors and the stability of inhibitor-bound complexes were evaluated and characterized with free energy profiles computed with state-of-the-art QM/MM MD simulations. We also accounted for the differences between urea and inhibitors in the binding mode and interactions with nearby residues. Emphasis was further placed on elucidating the possible connections between the calculated results, such as the structural and thermodynamic features, and inhibition efficiency.

Analysis of the structural dynamics of the reaction of urease with the three inhibitors underscored the similarities in their binding mechanisms. AHA, HU, and NBPTO have in common that each of their carbonyl or phosphate oxygen atoms is primarily coordinated with a Ni1 ion and that the Ni1-bound oxygen atom forms a hydrogen bond with His222. AHA and HU are subject to a bidentate binding mode in the middle of the binding process, even though they finally form the chelated AHA- and HU-inhibited complexes. Unlike the urea hydrolysis reaction reported in our previous study, it was found that a protonated form of Asp363 appears to be stabilized in the three inhibitors’ final states. From a thermodynamical point of view, we demonstrated that the order of binding affinity for the three inhibitors is NBPTO >> AHA > HU based on their calculated reaction free energies of –34.8 ± 0.7, –27.1 ± 0.8, and –26.4 ± 0.7 kcal/mol; the fact that urease-catalyzed DAP formation from NBPTO occurs in a barrierless process; and the fact that binding of AHA requires a slightly smaller free energy barrier than that of HU. This order obtained from our computed results agrees well with the inhibitory activity in the descending order NBPTO (2.1 nM) >> AHA (42 μM) > HU (100 μM) in terms of increasing IC_50_ values.

Overall, it should be emphasized that hydrogen bonding with His222 and Ni2–N amine bond formation are crucial for anchoring an inhibitor to the active site and for stabilizing the intermediate and final product complex. These findings not only elucidate why the existing urease inhibitors are effective but also have implications for the design of new inhibitors.

## Data Availability

The data presented are available on request from the corresponding author, T.S.

## Supplementary Materials

The following supporting information can be downloaded at: https://www.mdpi.com/article/10.3390/molecules28062697/s1, Figure S1. Two different AHA-bound complexes (**^1^RS_QMa_** and **^1^RS_QMb_**) optimized at the UB3LYP-D3BJ/def2-SVP level of theory, Figure S2. Potential energy profile (in kcal/mol) and illustrations of the transition state, intermediate and product structures corresponding to the inhibition process of AHA obtained at the UB3LYP-D3BJ/def2-SVP level of theory, Figure S3. Two different HU-bound complexes (**^2^RS_QMa_** and **^2^RS_QMb_**) optimized at the UB3LYP-D3BJ/def2-SVP level of theory, Figure S4. Potential energy profile (in kcal/mol) and illustrations of the transition state, intermediate and product structures corresponding to the inhibition process of HU obtained at the UB3LYP-D3BJ/def2-SVP level of theory, Figure S5. Selected hydrogen bond distances between N(AHA) and O(Asp363) during a 25 ps QM/MM metadynamics simulation, Figure S6. Selected hydrogen bond distances between N(HU) and O(Asp363) during a 25 ps QM/MM metadynamics simulation, Figure S7. Selected hydrogen bond distances between N(HU) and O(Asp363) during a 15 ps QM/MM metadynamics simulation, Figure S8. Selected hydrogen bond distances between N(DAP) and O(Asp363) during a 50 ps QM/MM metadynamics simulation, Figure S9. HU-bound complexes (^2′^RS_QM_) optimized at the (a) UB3LYP-D3BJ/def2-SVP and (b) GFN2-xTB levels of theory. (c) Potential energy profile (in kcal/mol) and illustrations of the transition state, intermediate and product structures corresponding to the hydrolysis process of HU obtained at the UB3LYP-D3BJ/def2-SVP and GFN2-xTB levels of theory, Figure S10. RMSD with respect to the initial conformations during a 10 ns classical MD simulation for an AHA-bound complex (1), Figure S11. RMSD with respect to the initial conformations during a 10 ns classical MD simulation for an HU-bound complex (2), Figure S12. RMSD with respect to the initial conformations during a 10 ns classical MD simulation for an HU-bound complex (2′), Figure S13. RMSD with respect to the initial conformations during a 10 ns classical MD simulation for an NBPTO-bound complex (3), and Cartesian coordinates for QM/MM MD equilibrated and QM-only optimized structures.

## Sample Availability

Samples of the compounds are not available from the authors.
