# Supplementary material for: Unraveling Binding Mechanism and Stability of Urease Inhibitors: A QM/MM MD Study
Source: Molecules. 2023 Mar 16;28(6):2697. doi: 10.3390/molecules28062697 (PMC10051795; doi:10.3390/molecules28062697)
Supplement: Supplementary file 1 [file molecules-28-02697-s001.zip › molecules-2217764-supplementary.pdf]

molecules

Supplementary Materials

Unraveling Binding Mechanism and Stability of Urease Inhibitors: A QM/MM MD Study

Shunya Suenaga [1], Yu Takano [2] and Toru Saito [2,*]

[1] Faculty of Information Sciences, Hiroshima City University, 3-4-1 Ozuka-Higashi, Asa-Minami-Ku, Hiroshima 731-3194, Japan
[2] Graduate School of Information Sciences, Hiroshima City University, 3-4-1 Ozuka-Higashi, Asa-Minami-Ku, Hiroshima 731-3194, Japan
* Correspondence: tsaito@hiroshima-cu.ac.jp; Tel.: +81-82-830-1617

Contents

Section 1: AHA-bound complexes (^{1}**RS**$_{QMa}$ and ^{1}**RS**$_{QMb}$) (Figures S1) and the reaction mechanism of AHA inhibition process obtained from QM-only (UB3LYP-D3BJ) computations (Figure S2)

Section 2: HU-bound complexes (^{2}**RS**$_{QMa}$ and ^{2}**RS**$_{QMb}$) (Figures S3) and the reaction mechanism of AHA inhibition process obtained from QM-only (UB3LYP-D3BJ) computations (Figure S4)

Section 3: The reaction mechanism of HU hydrolysis process obtained from QM-only (UB3LYP-D3BJ and GFN2-xTB) computations (Figure S5)

Section 4: Analysis of hydrogen bonds between inhibitors and Asp363 during QM/MM metadynamics simulation (Figure S6–S9)

Section 5: Results of classical MD simulations for inhibitor-bound complexes (Figures S10–S13)

Section 6: Cartesian coordinates for QM/MM MD equilibrated and QM-only optimized structures

Section 1: AHA-bound complexes ($^1RS_{QMa}$ and $^1RS_{QMb}$) and the reaction mechanism of AHA inhibition process obtained from QM-only (UB3LYP-D3BJ) computations

As shown in Figure S1, the UB3LYP-D3BJ/def2-SVP geometry optimizations using the truncated active-site (QM-only cluster) model defined in section 3.3 gave two different AHA-bound complexes (**$^1RS_{QMa}$** and **$^1RS_{QMb}$**). In **$^1RS_{QMa}$**, AHA chelates Ni1 and its OH group forms a hydrogen bond with WB. On the other hand, **$^1RS_{QMb}$** binds to the dinickel center in a bidentate manner, with bond lengths of 2.26 and 2.34 Å between Ni1 and the carbonyl oxygen and Ni2 and hydroxylamine oxygens. The coordination modes of carboxylate ligands (Lys220* and Asp363) are different from **$^1RS_{QMa}$** and **$^1RS_{QM}$**, and **$^1RS_{QMb}$** is 12.4 kcal/mol less stable than **$^1RS_{QMa}$**. Thus, we investigated the formation of AHA-inhibited complex starting with **$^1RS_{QMa}$**.

The reaction turns out to proceed via proton transfer from the hydroxylamine to WB, followed by the dissociation of the generated water molecule in line with the experimental suggestion (Figure S2). The formation of a reaction intermediate (**$^1Int_{QM}$**) is found to be feasible requiring a small activation barrier of only 1.1 kcal/mol and to be slightly exothermic. However, we could not locate a proper transition state that corresponds to the dissociation of the bridging water molecule. We constructed an initial guess for a product complex in which the water molecule was manually moved away from the nickel ions. The geometry optimization led to **$^1P_{QM}$**, lying 5.1 kcal/mol above **RS_{QMa}**. These QM-only results indicate that the binding of AHA inhibitor is endothermic albeit with its high inhibitory activity established as a standard reference. Therefore, we conclude that the QM-only cluster approach is insufficient for the prediction of mechanisms of binding and inhibition of AHA, possibly due to lack of taking into account of the protein environment.

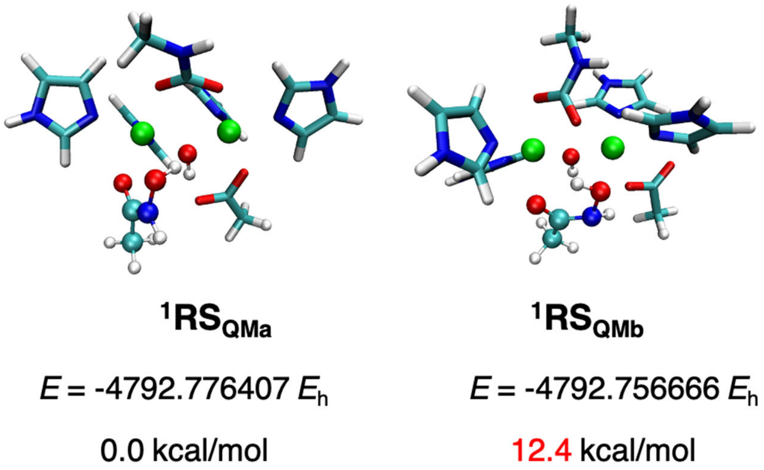

Figure S1. Two different AHA-bound complexes (**$^1RS_{QMa}$** and **$^1RS_{QMb}$**) optimized at the UB3LYP-D3BJ/def2-SVP level of theory.

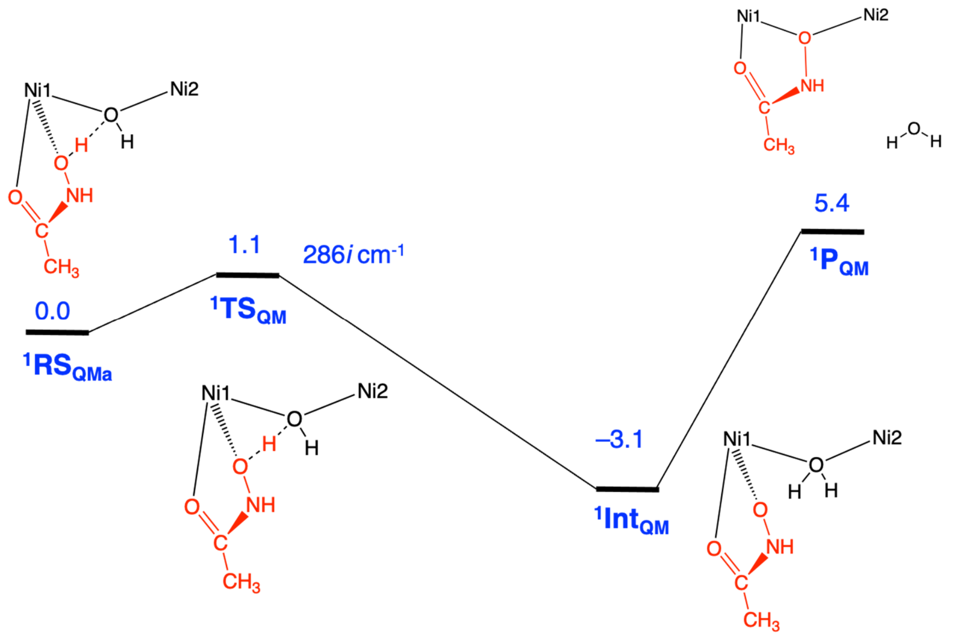

Figure S2. Potential energy profile (in kcal/mol) and illustrations of the transition state, intermediate and product structures corresponding to the inhibition process of AHA obtained at the UB3LYP-D3BJ/def2-SVP level of theory.

Section 2: HU-bound complexes ($^2RS_{QMa}$ and $^2RS_{QMb}$) and the reaction mechanism of AHA inhibition process obtained from QM-only (UB3LYP-D3BJ) computations

As shown in Figures S3 and S4, the UB3LYP-D3BJ calculations on the binding of HU inhibitor also highlight that QM-only cluster approach is not suitable for the prediction of mechanisms of binding and inhibition of HU.

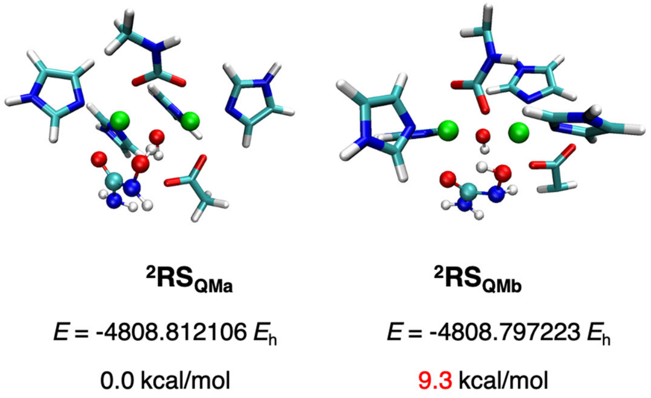

Figure S3. Two different HU-bound complexes (**$^2RS_{QMa}$** and **$^2RS_{QMb}$**) optimized at the UB3LYP-D3BJ/def2-SVP level of theory.

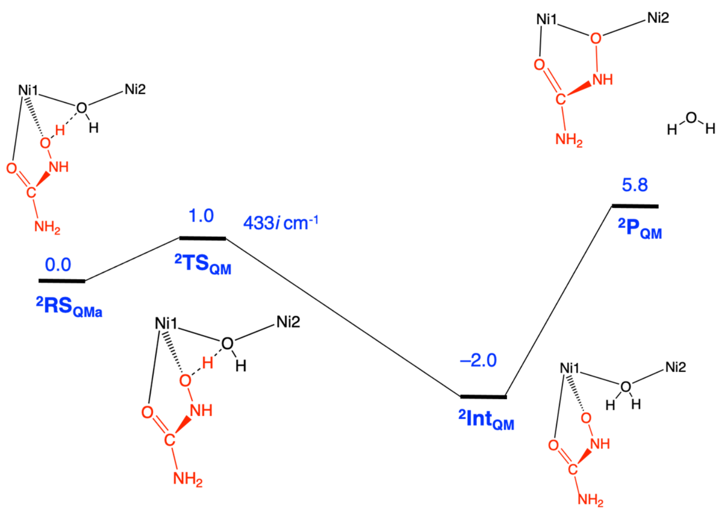

Figure S4. Potential energy profile (in kcal/mol) and illustrations of the transition state, intermediate and product structures corresponding to the inhibition process of HU obtained at the UB3LYP-D3BJ/def2-SVP level of theory.

Section 3: Analysis of hydrogen bonds between inhibitors and Asp363 during QM/MM metadynamics simulation

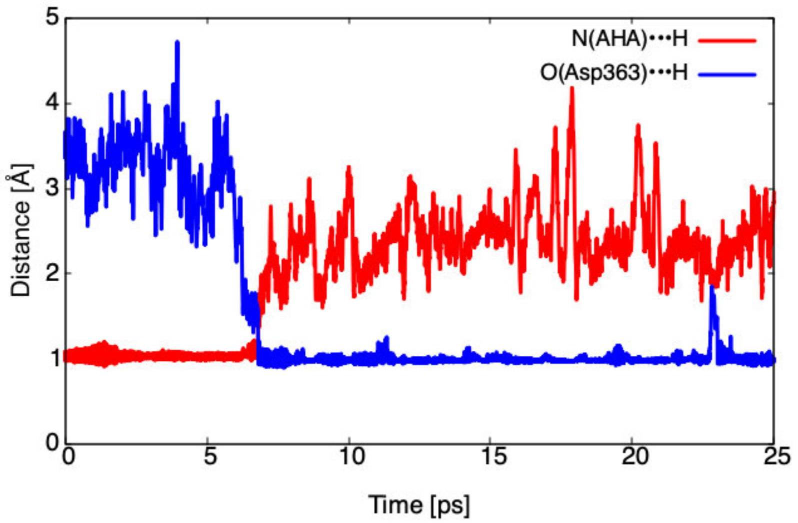

Figure S5. Selected hydrogen bond distances between N(AHA) and O(Asp363) during a 25 ps QM/MM metadynamics simulation.

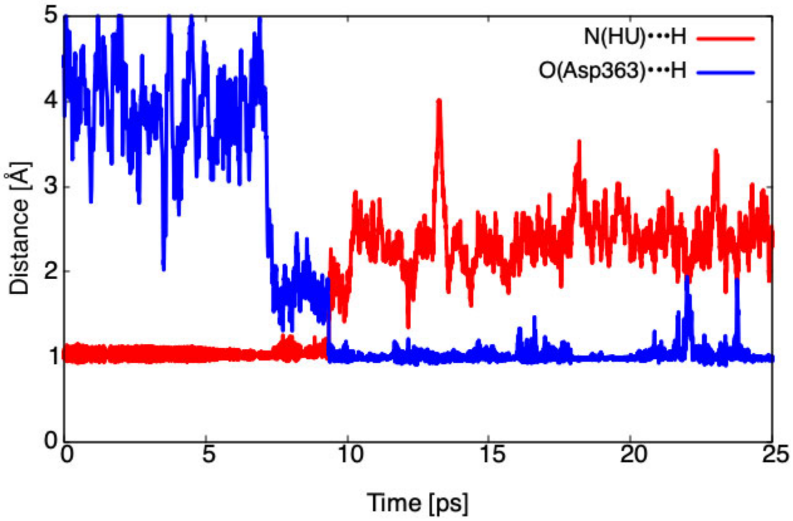

Figure S6. Selected hydrogen bond distances between N(HU) and O(Asp363) during a 25 ps QM/MM metadynamics simulation.

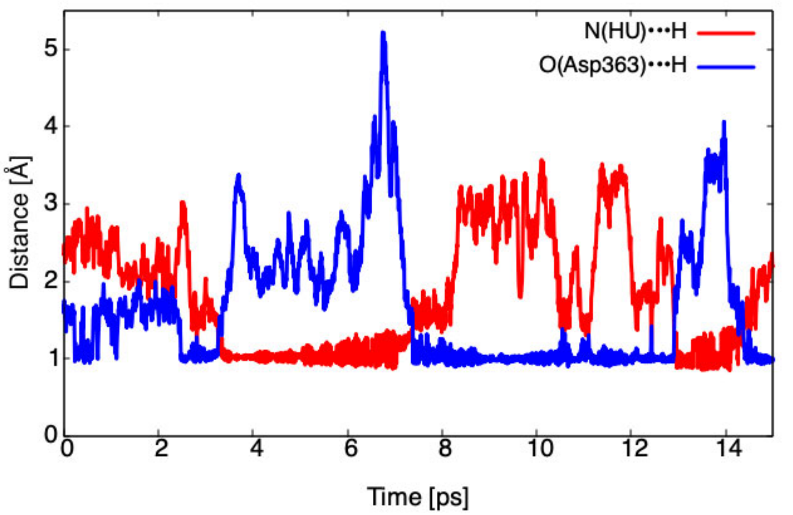

Figure S7. Selected hydrogen bond distances between N(HU) and O(Asp363) during a 15 ps QM/MM metadynamics simulation.

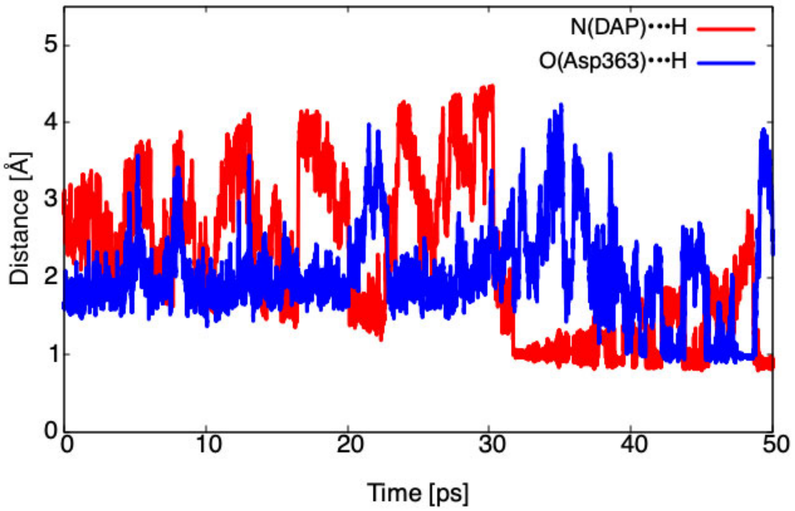

Figure S8. Selected hydrogen bond distances between N(DAP) and O(Asp363) during a 50 ps QM/MM metadynamics simulation.

Section 4: The reaction mechanism of HU hydrolysis process obtained from QM-only (UB3LYP-D3BJ and GFN2-xTB) computations

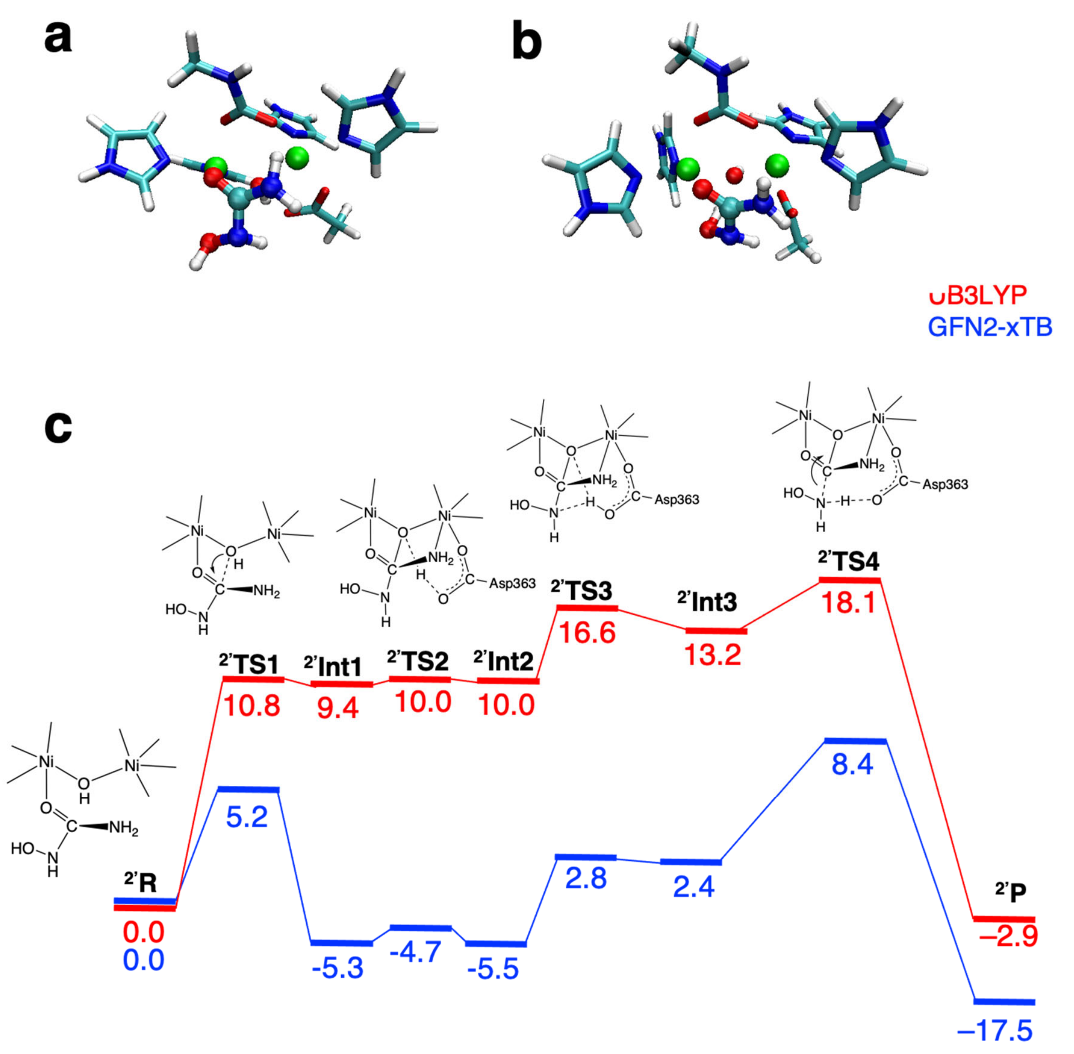

Figure S9. HU-bound complexes ($^{2'}$**RS**$_{QM}$) optimized at the (a) UB3LYP-D3BJ/def2-SVP and (b) GFN2-xTB levels of theory. (c) Potential energy profile (in kcal/mol) and illustrations of the transition state, intermediate and product structures corresponding to the hydrolysis process of HU obtained at the UB3LYP-D3BJ/def2-SVP and GFN2-xTB levels of theory.

Section 5: Results of classical MD simulations for inhibitor-bound complexes

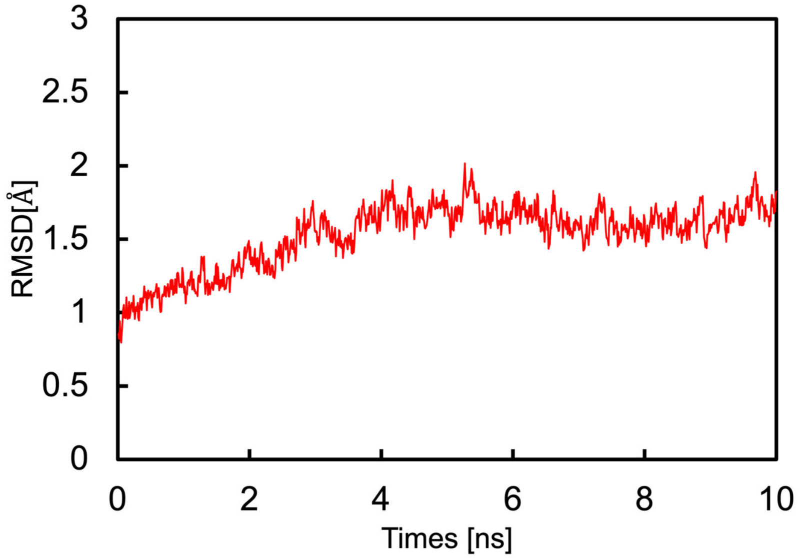

Figure S10. RMSD with respect to the initial conformations during a 10 ns classical MD simulation for an AHA-bound complex (**1**).

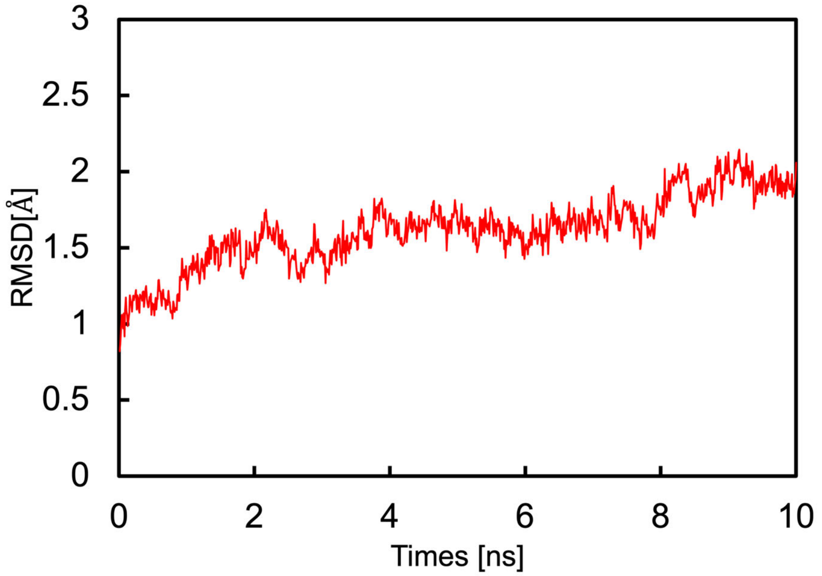

Figure S11. RMSD with respect to the initial conformations during a 10 ns classical MD simulation for an HU-bound complex (**2**).

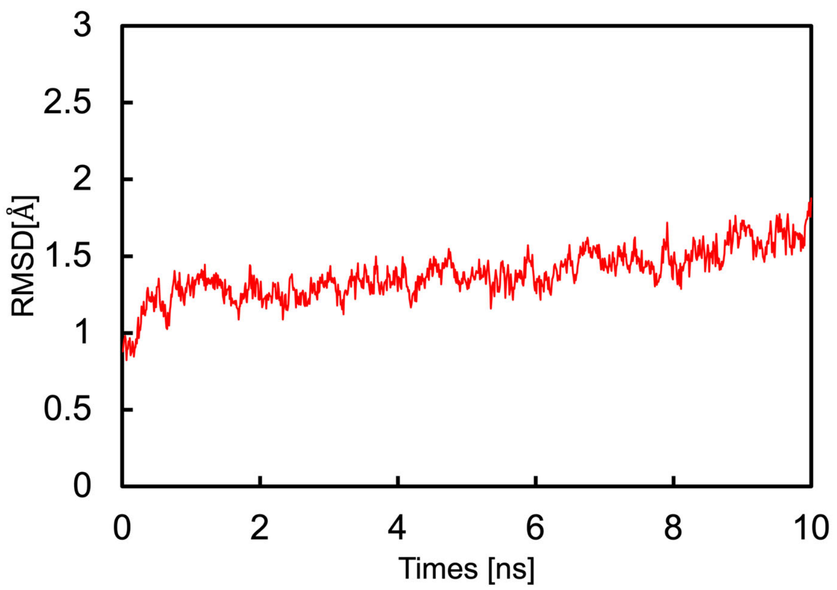

Figure S12. RMSD with respect to the initial conformations during a 10 ns classical MD simulation for an HU-bound complex (**2'**).

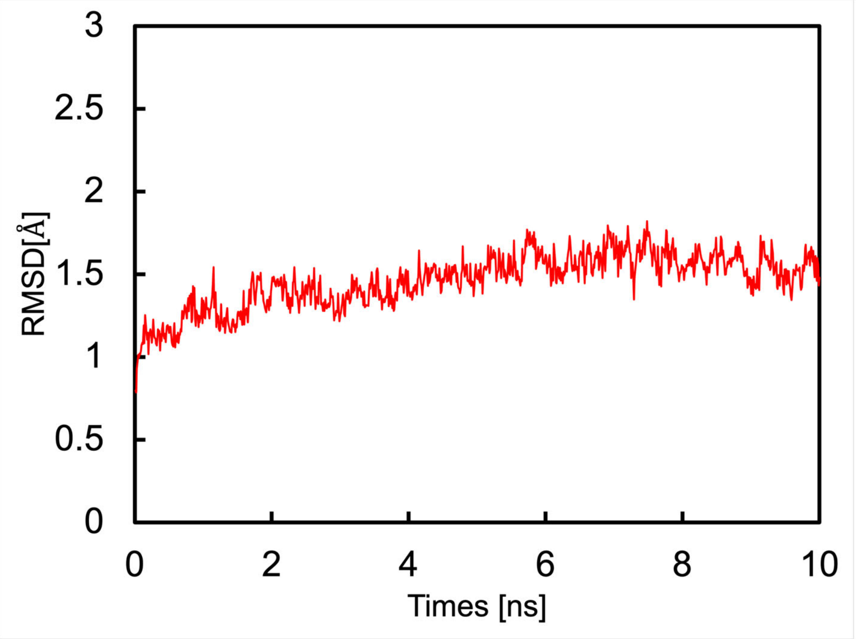

Figure S13. RMSD with respect to the initial conformations during a 10 ns classical MD simulation for an NBPTO-bound complex (**3**).

Section 6. Cartesian coordinates for QM/MM MD equilibrated and QM-only optimized structures.

The Cartesian coordinates of the QM atoms after QM/MM MD equilibration

[1]RS

C 3.520486 -8.886891 0.065882

H 3.260500 -8.337237 0.958674

H 4.329604 -8.369802 -0.428173

N 5.363554 -10.120766 0.987536
H 5.932014 -9.289942 0.955132
C 4.084709 -10.162296 0.520902
C 5.794266 -11.368177 1.204951
H 6.792806 -11.566065 1.399841
N 4.830903 -12.209895 0.905773
C 3.747636 -11.466487 0.454249
H 2.871631 -11.960615 0.156933
C -1.101490 -15.326490 1.009244
H -1.317062 -16.349956 0.667430
H -1.508092 -15.201462 1.971661
N 1.115588 -16.307522 0.686453
H 0.749078 -17.173528 0.249931
C 0.351310 -15.205371 0.931130
C 2.401584 -16.047523 0.924873
H 3.117345 -16.773997 0.734592
N 2.471956 -14.840220 1.434099
C 1.226658 -14.302479 1.501248
H 1.142354 -13.325443 1.833569
C 6.151383 -15.153649 -6.314915
H 5.611734 -14.218349 -6.290071
H 5.472479 -15.979840 -6.672274
C 6.572959 -15.623739 -4.918648
H 5.681620 -16.009119 -4.342046
H 7.343467 -16.413859 -5.060198
C 7.324070 -14.587832 -4.050866
H 8.372974 -14.343183 -4.321159
H 6.745356 -13.653622 -4.119029
C 7.400416 -15.160978 -2.652272
H 7.743699 -16.190782 -2.736052
H 8.116675 -14.583336 -2.061676
N 6.138034 -15.178912 -1.939293
H 5.377765 -14.601833 -2.303832
C 6.127774 -15.041897 -0.574793
O 5.106254 -14.734152 0.009987
O 7.224046 -15.326173 -0.018025
C 8.504872 -19.089208 -2.568434
H 8.120948 -19.995994 -3.091043
H 7.894480 -18.232904 -2.820319
N 8.695506 -20.245027 -0.303131
C 8.377948 -19.146553 -1.090666
C 8.332319 -19.907278 0.931955
H 8.598443 -20.570054 1.731611

N 7.783134 -18.678517 0.993068
H 7.649628 -18.075591 1.803079
C 7.714015 -18.255917 -0.309075
H 7.374754 -17.234726 -0.520788
C 10.500155 -15.710763 -0.479485
H 10.132971 -16.511946 -1.147566
H 9.690076 -14.984533 -0.280790
N 9.784154 -16.497539 1.735029
C 10.825916 -16.410805 0.831979
C 10.158632 -17.252535 2.752220
H 9.673283 -17.406856 3.669376
N 11.396686 -17.715001 2.483154
H 11.946453 -18.415201 2.955849
C 11.861506 -17.118192 1.329521
H 12.893783 -17.246006 1.111002
C 9.808069 -10.532300 4.402771
H 8.772421 -10.224157 4.558410
H 10.267790 -10.783177 5.340436
N 10.884067 -12.215586 2.843725
H 11.713851 -11.686857 2.625520
C 9.810521 -11.786567 3.583738
C 10.504626 -13.373934 2.219969
H 11.153699 -13.950184 1.603182
N 9.262440 -13.679717 2.516200
C 8.868217 -12.739839 3.381511
H 7.852212 -12.764073 3.728495
C 7.100512 -23.074560 6.294601
H 7.709695 -23.568805 5.531274
H 7.503323 -23.285130 7.260975
N 7.179360 -21.011682 4.789145
H 6.815166 -21.466877 3.947364
C 7.288826 -21.633182 6.018487
C 7.391603 -19.702231 4.962387
H 7.366663 -18.972921 4.194455
N 7.755238 -19.466066 6.184178
C 7.698784 -20.632729 6.838643
H 7.895010 -20.722509 7.896459
C 3.793768 -10.979793 4.605788
H 3.669049 -11.033834 5.690561
H 4.391670 -10.209484 4.271133
C 4.449976 -12.266327 4.078870
O 3.689748 -13.027597 3.383561
O 5.562668 -12.507689 4.445562

N 0.322504 -15.179385 6.316062
H 0.426781 -14.194321 6.230625
C 1.483566 -15.828624 5.791821
H 1.569080 -16.867922 6.109336
C 1.568448 -15.742803 4.272698
H 1.922370 -14.751406 4.045367
H 0.619945 -15.890524 3.765770
H 2.259540 -16.448489 3.870415
C 2.661188 -15.222156 6.505998
O 3.664879 -14.846039 5.926428
NI 7.935331 -15.277499 1.887916
NI 4.432225 -14.011419 1.714599
O 6.355850 -14.240780 2.430821
H 6.322964 -13.490664 3.080551
C 6.342067 -16.380156 4.200116
O 7.294118 -16.653706 3.437795
N 5.044689 -16.414867 3.788714
H 4.316711 -15.871436 4.294023
O 4.752525 -16.456249 2.426247
H 5.630911 -16.374141 1.982034
C 6.601046 -16.076236 5.603062
H 6.462814 -14.956261 5.621478
H 5.850304 -16.473295 6.259634
H 7.615103 -16.309149 5.963885
H 2.637309 -9.053488 -0.550838
H -1.605510 -14.631688 0.337445
H 6.919526 -15.073008 -7.084040
H 9.505738 -18.931648 -2.970357
H 11.374828 -15.220783 -0.907232
H 10.309630 -9.698258 3.911944
H 6.053195 -23.374443 6.258578
H 2.849480 -10.829137 4.082601
H -0.572769 -15.598354 6.260828
H 2.485912 -15.063053 7.569983

^{2}RS

C 3.204658 -9.367727 0.272543
H 2.870409 -8.769241 1.159108
H 3.857503 -8.616605 -0.231943
N 5.181540 -10.643866 1.017532
H 5.726479 -9.803595 1.089708
C 3.826133 -10.660576 0.656862
C 5.511580 -11.953727 1.186700

H 6.515844 -12.276059 1.189583
N 4.504572 -12.717734 1.000016
C 3.437455 -11.936131 0.679422
H 2.463728 -12.357680 0.574958
C -1.207987 -15.789928 0.736550
H -1.557635 -16.817975 0.561821
H -1.638393 -15.522887 1.698380
N 1.050020 -16.545309 0.154454
H 0.740883 -17.272571 -0.465119
C 0.242467 -15.701615 0.870271
C 2.344577 -16.326232 0.533048
H 3.144725 -16.930143 0.229552
N 2.435061 -15.335132 1.368158
C 1.169703 -14.939671 1.597165
H 0.997445 -14.118417 2.255773
C 5.683433 -15.144448 -6.182182
H 5.065608 -14.283409 -6.036440
H 5.147571 -15.941132 -6.676121
C 6.190225 -15.633409 -4.753286
H 5.316331 -15.852238 -4.127820
H 6.645561 -16.628284 -4.827883
C 7.049022 -14.604940 -3.994431
H 7.973668 -14.379444 -4.540674
H 6.514640 -13.654761 -4.009987
C 7.369459 -15.033501 -2.586034
H 7.874693 -16.003321 -2.599532
H 8.026032 -14.217843 -2.125819
N 6.170524 -15.146363 -1.765750
H 5.283260 -14.943101 -2.173777
C 6.179554 -15.265222 -0.407409
O 5.060822 -15.372138 0.118157
O 7.284744 -15.217560 0.125634
C 8.411402 -19.338529 -2.528454
H 8.277741 -20.388470 -2.629260
H 7.587553 -18.882436 -3.098477
N 8.692767 -19.515915 0.008583
C 8.190704 -18.881391 -1.109077
C 8.062047 -18.870595 0.962797
H 8.230663 -19.021366 2.014128
N 7.167522 -17.983723 0.634299
H 6.699269 -17.338788 1.226627
C 7.199347 -17.994688 -0.718435
H 6.563673 -17.410212 -1.315600

C 10.275593 -15.532669 -0.380510
H 9.833634 -16.198364 -1.133346
H 9.605297 -14.780057 -0.085251
N 9.451510 -16.211110 1.904184
C 10.392037 -16.300105 0.885041
C 9.692500 -17.121227 2.807541
H 9.118046 -17.238651 3.716945
N 10.900013 -17.693899 2.557646
H 11.331367 -18.353538 3.231337
C 11.351004 -17.161406 1.363259
H 12.293217 -17.458058 0.962843
C 9.524422 -10.087668 4.596511
H 8.469146 -9.806340 4.739930
H 9.972110 -10.136316 5.604290
N 10.637674 -12.020785 3.651777
H 11.559830 -11.577689 3.745819
C 9.466397 -11.376028 3.940480
C 10.370669 -13.206686 3.103780
H 11.141166 -13.882780 2.866189
N 9.074267 -13.367173 2.943611
C 8.509991 -12.259172 3.483395
H 7.428701 -12.149698 3.399709
C 7.078290 -22.633239 6.852176
H 7.762609 -23.289810 6.271808
H 7.457105 -22.585467 7.813183
N 6.922072 -21.095850 4.957652
H 6.763189 -21.875849 4.304605
C 7.179719 -21.238396 6.286366
C 7.136275 -19.784931 4.728115
H 6.974108 -19.356249 3.749056
N 7.482769 -19.157719 5.795733
C 7.577891 -20.037047 6.789814
H 7.774758 -19.729717 7.843564
C 3.347411 -11.688610 4.647017
H 3.219731 -11.694536 5.732618
H 4.114988 -10.983780 4.462469
C 3.894645 -13.196653 4.421242
O 3.313400 -13.813847 3.497589
O 4.906314 -13.581263 5.012116
N -0.420480 -15.606791 5.862506
H -0.312157 -14.624619 5.786585
C 0.734636 -16.402431 5.560801
H 0.721516 -17.400334 5.977953

C 1.063585 -16.348434 4.058343
H 1.693722 -15.464533 3.804076
H 0.147161 -16.398380 3.488551
H 1.705914 -17.207325 3.844959
C 1.960640 -15.809743 6.252055
O 3.102041 -15.776580 5.879784
NI 7.954114 -14.971659 2.167682
NI 4.326787 -14.607437 1.901146
O 6.141586 -14.282851 2.394175
H 5.925109 -13.787755 3.182847
C 5.818838 -16.589225 4.024654
O 6.706865 -17.227916 3.434261
N 4.576345 -16.301858 3.438611
H 4.295724 -16.951294 2.731712
H 3.835401 -16.045508 4.093403
N 5.947742 -16.069156 5.274263
H 5.429380 -15.222783 5.490606
O 7.010682 -16.457230 6.071698
H 7.752881 -16.361087 5.430964
H 2.380864 -9.445196 -0.437008
H -1.520071 -15.126746 -0.070229
H 6.506654 -14.897336 -6.852512
H 9.390997 -19.051127 -2.910414
H 11.239228 -15.154098 -0.721380
H 9.978561 -9.236594 4.089035
H 6.073200 -23.048857 6.780374
H 2.433831 -11.458465 4.098835
H -1.320635 -15.996081 5.997685

2'RS C 3.681473 -9.098704 -0.480451
H 3.663005 -8.200350 0.129126
H 4.399810 -8.925121 -1.250823
N 5.428214 -10.390209 0.707667
H 6.180334 -9.714809 0.726847
C 4.117091 -10.232572 0.312933
C 5.622382 -11.705764 1.063284
H 6.546363 -12.128448 1.431123
N 4.392809 -12.333122 1.098463
C 3.491482 -11.417703 0.657799
H 2.498237 -11.733918 0.416566
C -1.193581 -15.529778 0.843963
H -1.467416 -16.520134 0.669058
H -1.596858 -15.231257 1.839897

N 0.935421 -16.809669 0.697036
H 0.559728 -17.708101 0.430024
C 0.357433 -15.580674 0.891581
C 2.295937 -16.588666 0.956113
H 3.121866 -17.289068 0.794777
N 2.551480 -15.329923 1.185463
C 1.317227 -14.694946 1.269516
H 1.314643 -13.624413 1.369561
C 6.062016 -14.962287 -6.328867
H 5.534390 -14.036265 -6.203873
H 5.332635 -15.686187 -6.594298
C 6.554378 -15.425655 -4.866916
H 5.674464 -15.748942 -4.307483
H 7.187257 -16.214844 -5.070533
C 7.317125 -14.330193 -4.037503
H 8.287826 -14.112298 -4.458883
H 6.739772 -13.397317 -3.950151
C 7.519491 -14.973842 -2.630271
H 7.954676 -15.956142 -2.740147
H 8.165197 -14.337732 -1.950861
N 6.311289 -14.990892 -1.873067
H 5.462993 -15.273352 -2.368487
C 6.251684 -14.847627 -0.531982
O 5.138648 -15.057369 0.072318
O 7.411658 -14.682232 0.021171
C 8.933743 -18.749675 -2.681576
H 8.540198 -19.658873 -3.161930
H 8.317547 -17.854433 -3.101150
N 9.111940 -19.659914 -0.312795
C 8.762392 -18.671179 -1.160684
C 8.623646 -19.301016 0.884243
H 8.696493 -19.809355 1.830677
N 7.927839 -18.160946 0.777761
H 7.585233 -17.509447 1.493764
C 8.101727 -17.734897 -0.513916
H 7.662301 -16.814262 -0.776250
C 10.615337 -15.384189 -0.780235
H 10.215825 -16.133435 -1.484035
H 9.817734 -14.656074 -0.682013
N 9.911808 -15.849425 1.609770
C 10.742968 -16.089103 0.545019
C 10.289887 -16.715599 2.555953
H 9.901453 -16.771094 3.573722

N 11.303922 -17.510919 2.126741
H 11.654726 -18.248616 2.688758
C 11.574716 -17.166789 0.825730
H 12.221412 -17.623969 0.162165
C 9.771808 -10.067087 4.372425
H 8.741062 -9.753311 4.480346
H 10.098763 -10.376033 5.349545
N 10.807973 -11.912324 2.827361
H 11.797861 -11.697410 2.942739
C 9.769739 -11.265491 3.488832
C 10.267813 -12.816137 1.991173
H 10.849031 -13.527512 1.469916
N 8.930189 -12.868547 2.147422
C 8.651076 -11.876106 2.995786
H 7.620682 -11.658220 3.229339
C 7.151763 -23.164950 6.224374
H 7.742657 -23.656532 5.484368
H 7.706778 -23.324849 7.139532
N 6.627165 -21.151626 4.662645
H 6.335128 -21.758093 3.925303
C 7.166396 -21.651240 5.830433
C 6.934413 -19.823737 4.548952
H 6.748869 -19.315852 3.645116
N 7.632027 -19.481847 5.595118
C 7.849017 -20.573737 6.357239
H 8.319919 -20.564551 7.306333
C 2.867961 -11.479808 4.670592
H 2.620449 -11.687259 5.696552
H 3.527594 -10.651158 4.738285
C 3.723694 -12.699058 4.196614
O 3.229488 -13.470407 3.386754
O 4.871378 -12.717215 4.707171
N -0.337523 -16.235067 6.192585
H -0.169861 -15.880889 7.112849
C 0.844177 -16.589454 5.412581
H 0.897489 -17.686983 5.618581
C 0.852335 -16.277586 3.943958
H -0.011029 -15.632373 3.761188
H 0.748122 -17.149804 3.327709
H 1.837269 -15.758538 3.751686
C 2.034978 -16.021549 6.125529
O 3.051122 -15.629539 5.565978
NI 7.984424 -14.784977 2.036100

NI 4.375062 -14.347566 1.773907
O 6.131310 -14.158683 2.867600
H 6.005396 -13.473488 3.524644
C 6.007481 -16.347783 3.647829
O 7.045227 -16.645369 3.035414
N 4.811807 -16.673429 3.165392
H 4.778812 -16.930588 2.183376
H 3.951288 -16.601768 3.721651
N 5.939754 -15.920870 4.887996
H 5.223564 -15.157046 5.133215
O 7.213215 -15.630301 5.418695
H 7.573690 -14.892121 4.879371
H 2.723184 -9.190414 -0.991696
H -1.607075 -14.818295 0.129180
H 6.843407 -14.884056 -7.084782
H 9.950269 -18.614383 -3.050995
H 11.543602 -14.946308 -1.147229
H 10.334317 -9.236544 3.945948
H 6.166883 -23.625286 6.303127
H 1.988939 -11.264228 4.063183
H -1.253762 -16.320519 5.827477
H 1.919848 -15.885404 7.200848

^{3}RS C 3.204943 -9.050707 0.113515
H 2.725867 -8.642854 1.041628
H 3.985584 -8.279739 -0.157326
N 5.037750 -10.143773 1.230074
H 5.400263 -9.242012 1.493941
C 3.914627 -10.281876 0.533213
C 5.449673 -11.356372 1.540961
H 6.289504 -11.551493 2.215713
N 4.654471 -12.316010 1.094296
C 3.666976 -11.624262 0.388458
H 2.766130 -12.062838 -0.020274
C -1.229610 -15.470445 1.031340
H -1.512264 -16.496022 0.782615
H -1.692608 -15.175127 1.955997
N 0.975308 -16.478604 0.484741
H 0.615301 -17.330961 0.117509
C 0.277843 -15.459491 1.057286
C 2.281892 -16.222613 0.706355
H 3.083374 -16.825297 0.436176
N 2.481378 -15.057613 1.349899

C 1.202668 -14.598657 1.579974
H 1.056530 -13.687054 2.135098
C 5.629453 -15.072491 -6.226022
H 5.178781 -14.078130 -6.041373
H 4.830174 -15.730949 -6.472167
C 6.221075 -15.530524 -4.916800
H 5.416303 -15.909451 -4.234160
H 6.894808 -16.376700 -4.950315
C 7.007532 -14.428851 -4.280175
H 7.896558 -14.276240 -4.843236
H 6.448020 -13.501247 -4.338507
C 7.399466 -14.708838 -2.762802
H 7.970929 -15.620245 -2.676292
H 8.066388 -13.888570 -2.386107
N 6.184910 -14.755125 -1.968466
H 5.507301 -14.054814 -2.272249
C 6.251426 -14.895251 -0.568244
O 5.151062 -14.912836 -0.000304
O 7.385361 -15.202490 -0.056086
C 8.502805 -19.533255 -2.964821
H 8.246109 -20.546487 -3.338727
H 7.762272 -18.873708 -3.455726
N 8.278254 -20.356791 -0.600435
C 8.130136 -19.383062 -1.502845
C 7.847122 -19.897351 0.550929
H 7.851963 -20.494286 1.443297
N 7.355877 -18.648348 0.438755
H 7.001021 -18.070444 1.193498
C 7.627788 -18.253491 -0.878651
H 7.331065 -17.312606 -1.259272
C 10.157677 -15.801989 -0.781967
H 9.836922 -16.522973 -1.470501
H 9.369807 -15.029083 -0.830439
N 9.607844 -16.008408 1.726977
C 10.113950 -16.458288 0.556252
C 9.595682 -17.069166 2.556916
H 9.268162 -16.996930 3.589464
N 10.149961 -18.158752 1.965472
H 10.067695 -19.085350 2.372266
C 10.416742 -17.797004 0.709332
H 10.938584 -18.485037 0.077604
C 10.119543 -10.242887 4.346566
H 9.118214 -9.888121 4.574846

H 10.603714 -10.471621 5.322685
N 10.917827 -12.002850 2.735006
H 11.852458 -11.666183 2.621646
C 9.965209 -11.433229 3.500191
C 10.319104 -13.027153 2.070746
H 10.862092 -13.624020 1.375043
N 9.070674 -13.192564 2.423213
C 8.865312 -12.185183 3.342573
H 7.910356 -12.138372 3.896538
C 7.448797 -22.585641 6.447368
H 8.066333 -23.006079 5.633222
H 7.812330 -22.872413 7.428644
N 7.908212 -20.526023 5.205062
H 8.322089 -20.891092 4.367756
C 7.456880 -21.111992 6.351708
C 7.965907 -19.106810 5.442907
H 7.970372 -18.492266 4.578504
N 7.541384 -18.883262 6.664548
C 7.173364 -20.043822 7.188639
H 6.651432 -20.015022 8.113555
C 3.608635 -11.152477 4.702013
H 3.370592 -11.159515 5.794280
H 4.356415 -10.459400 4.373775
C 4.243307 -12.460151 4.152701
O 3.533363 -13.236480 3.496178
O 5.480874 -12.503886 4.367986
N -0.089689 -15.289088 5.838756
H 0.104271 -14.335715 5.595152
C 0.967709 -16.189888 5.992767
H 0.752045 -17.035782 6.679980
C 1.326043 -16.711340 4.641399
H 1.526320 -15.971586 3.953803
H 0.565602 -17.443458 4.315030
H 2.164163 -17.347651 4.793034
C 2.193706 -15.406687 6.559608
O 3.101126 -15.220277 5.795331
NI 7.911723 -14.919837 1.779403
NI 4.370422 -14.226056 1.924321
O 6.307886 -14.537897 2.869593
H 6.253857 -13.744316 3.466434
O 6.795253 -16.890264 2.548934
N 6.713788 -16.075730 4.979884
H 7.413600 -15.445653 4.659805

N 4.398057 -16.140610 3.327126
H 3.648106 -16.737809 3.064869
H 3.995965 -15.470993 4.001729
P 5.869969 -16.748917 3.717217
C 6.035605 -15.516265 6.161497
H 5.157191 -16.099768 6.361509
H 5.789551 -14.456834 6.013149
C 6.910527 -15.781331 7.433749
H 7.283735 -16.795176 7.378056
H 7.792551 -15.176530 7.494852
C 6.041095 -15.619650 8.676168
H 5.195110 -16.333176 8.640106
H 5.652660 -14.598862 8.734486
C 6.884114 -16.005335 9.911583
H 6.357286 -15.806221 10.866840
H 7.083480 -17.087274 9.857264
H 7.827097 -15.489433 9.928394
N 5.306771 -18.331361 4.251750
H 5.919183 -19.059631 4.138564
H 4.993248 -18.541080 5.227183
H 2.447907 -9.139645 -0.665642
H -1.583837 -14.882364 0.184708
H 6.341871 -15.039332 -7.050317
H 9.510280 -19.252563 -3.271915
H 11.067220 -15.341649 -1.167868
H 10.695783 -9.443422 3.880837
H 6.447562 -23.014273 6.403753
H 2.685220 -10.940649 4.163001
H -1.020556 -15.615561 5.922437
H 2.181021 -15.076744 7.598394

The Cartesian coordinates for AHA- and HU- complexes optimized at the UB3LYP-D3BJ/def2-SVP level (^{1}**RS**$_{QMa}$, ^{1}**RS**$_{QMb}$, ^{2}**RS**$_{QMa}$, and ^{2}**RS**$_{QMb}$ see also Section 1).

^{1}RS$_{QMa}$

N	6.48814780874553	-10.85627873395009	0.19191444254459
H	7.23462480715227	-10.44179105070998	-0.35242895790624
C	5.55171758868108	-10.16979095418600	0.94398259798247
C	6.21988782801015	-12.18267759794309	0.28056601051518
H	6.80686396349864	-12.97985900258871	-0.17377336039014
N	5.16594838318073	-12.36899800291128	1.05342370775003
C	4.73249451380449	-11.13349101433723	1.47746675518486

H	3.87395883226804	-11.03757339419726	2.13646568846843
N	0.97200856115388	-13.85055124264085	-0.93018301959086
H	0.51200188339010	-13.97132273578975	-1.82500037637357
C	0.40255800990556	-13.31790108017235	0.21059249497717
C	2.25053372046348	-14.19487385695817	-0.64680090911856
H	2.95427196912825	-14.64331989259653	-1.34343734673968
N	2.52284725855869	-13.90843654184083	0.61489199815056
C	1.38548724207859	-13.36051457177210	1.16816219087146
H	1.37601069680058	-13.05085611489325	2.20908129730147
C	7.40025188372317	-17.45997643022739	-1.53910696033129
H	7.94284640833206	-17.72875771771816	-0.61975342703844
H	8.08629654426771	-16.87919450755972	-2.18094281817381
N	6.18669174264434	-16.73642046102039	-1.23501606603496
H	5.30613678624291	-17.04805540320064	-1.62333697876508
C	6.12222241213253	-15.66847391740728	-0.39037491349931
O	4.98056230367535	-15.15628828134282	-0.19485169360635
O	7.22625921222568	-15.25787234369573	0.11288591390662
N	9.56412202074546	-15.87803657605233	1.67848331634405
C	10.14835725260226	-15.96064684720998	0.43239904613252
C	10.46401324992283	-16.27396891710598	2.56313184648442
H	10.30469871436435	-16.31312602682429	3.63799042342766
N	11.61248618653780	-16.61220332948051	1.93028732438174
H	12.45227741273173	-16.96135038819458	2.37840610733969
C	11.43503188110868	-16.42077216514642	0.57415057196980
H	12.21612315608290	-16.63232844094492	-0.15053654924602
N	9.32347573186819	-11.37488775232355	2.42497992282826
H	10.03762472132967	-10.68764305618258	2.21383643380262
C	8.28791313579358	-11.22137226976823	3.32802069256112
C	9.25527297745539	-12.62984396911461	1.91490547781968
H	9.95431786290014	-13.03573280134506	1.18676712480280
N	8.22821439943849	-13.27168426955626	2.44249357231296
C	7.61115279710701	-12.41586918079673	3.32980868339384
H	6.73969621867905	-12.72594389753135	3.90512375553418
C	3.17394062963187	-12.68647015873968	5.50722682698384
H	3.59192436409092	-12.85658752376132	6.50724991700984
H	3.18281618351799	-11.60533936721221	5.29270658691402
C	3.98448615740551	-13.40865554001815	4.44504006446876
O	3.47629156408148	-13.44177306163592	3.27944316374311
O	5.09645001713933	-13.90140989137352	4.76183836213833
Ni	7.66450317345617	-15.20618535797758	2.11649558930578
Ni	4.35185169164993	-14.17487206172139	1.52559106304362
O	5.72975394872857	-15.14967034883598	2.56850936920068
H	5.58609523254630	-14.74107487623176	3.47067783218229

C	7.28842994618675	-16.26319485339916	4.93648098732979
O	8.05200748937331	-15.64152151855851	4.17033636445869
N	6.45032766523948	-17.21606880348196	4.51201558828597
H	5.77887490137293	-17.64916812905649	5.13987470191122
O	6.28639878293715	-17.50263630305363	3.18534538235917
H	5.84918195549529	-16.64327718916586	2.82571183057106
C	7.21949445364440	-15.92922147655061	6.39936187751371
H	6.60114451013960	-15.01957593872996	6.47841376125949
H	6.76597987448854	-16.72703654498641	7.00448362556496
H	2.12469439180288	-13.01431520466306	5.47099316359723
H	7.14130671376936	-18.38563111933279	-2.07119079253793
H	9.58550211400677	-15.69579332963557	-0.45748252205315
H	-0.62573831692721	-12.96931044958633	0.23924896012371
H	5.54833396471456	-9.08660821340125	1.02328200098238
H	8.12997771381169	-10.30020661177292	3.88107293498189
H	8.22787984896453	-15.70606718987973	6.77161825068752

[1]RS$_{QMb}$

N	5.11723994614320	-10.81620666401439	-0.57159376110543
H	5.68343276706593	-10.15446348238898	-1.08904913098219
C	3.73492840344189	-10.86519254826066	-0.55012799923608
C	5.58969952233842	-11.78984892775939	0.24394949841024
H	6.64077582082706	-12.00143181643136	0.42303322327331
N	4.57886420304640	-12.46129492509118	0.77179793238693
C	3.41496280901264	-11.89799353774992	0.29527793935085
H	2.44449689268947	-12.23874905139885	0.64026346329123
N	2.21605317266213	-16.91611313967266	0.38868170867725
H	2.06305957653239	-17.79808912753165	-0.08614476059181
C	1.23711388387294	-16.09237314482426	0.90800987386621
C	3.42165098550274	-16.35018414466814	0.63776707087919
H	4.38344340437251	-16.77767668613758	0.36342774485576
N	3.25851644875121	-15.20753506752064	1.28632842187919
C	1.90262120284542	-15.02886163529201	1.46746494908177
H	1.52971536710903	-14.15313034608983	1.99594667070207
C	6.64454501646989	-13.83486004311969	-2.48460454357806
H	6.52777391330190	-14.39075760720613	-3.42765287342920
H	7.70812522307807	-13.61206264764136	-2.33462358435370
N	6.18226386893363	-14.62454520632918	-1.36403923695477
H	5.23974066979961	-14.99489102950941	-1.37775032351680
C	6.81618754637393	-14.68569119017635	-0.16691170512030
O	6.24542357500657	-15.32920096712278	0.79516271801481
O	7.93538204084181	-14.11908487230983	0.00503318688287
N	9.23422149301406	-16.28721292393575	1.55419585980269

C	9.89559753541072	-16.44095962580724	0.35458673459857
C	9.46866272319959	-17.37263184815995	2.27496994325841
H	9.08164531879091	-17.55369921175377	3.27482713312572
N	10.26111039055731	-18.22332280443167	1.57953248128583
H	10.57924571901245	-19.13038896007292	1.90206735810996
C	10.54457798397143	-17.65125624700775	0.35531786067455
H	11.16411203031704	-18.14407946170744	-0.38846717006153
N	11.50966179543407	-12.82727489559177	3.64167523658957
H	12.45694373500236	-12.84854937715969	4.00274208192264
C	10.72124952818005	-11.70362098993706	3.50093489663911
C	10.80360125534897	-13.89947818981901	3.20791527903452
H	11.18008079184131	-14.91943588233165	3.20160089951793
N	9.60754953725018	-13.51586357272930	2.79938704792839
C	9.53447646263626	-12.15070945205552	2.97346650836754
H	8.63797357426343	-11.60036400901618	2.70231584005880
C	2.69737758669247	-12.35173365181983	5.40542076249697
H	3.57793089902230	-11.85415827404387	5.84063051724712
H	1.82803218432882	-11.68436576139085	5.45164514800344
C	2.97784501880771	-12.76213016807104	3.96768212126571
O	4.07274682290502	-13.45050992110042	3.80937156773175
O	2.20875311206963	-12.46392482582282	3.06043846493697
Ni	8.06787751797989	-14.65660603229330	2.06848249328758
Ni	4.82996810184268	-14.07633743400378	2.02014205884616
O	6.64256855757118	-13.37464031403739	2.56974521701776
H	6.61356906714837	-12.84008790362991	3.37061005258594
C	6.92820149492421	-15.22685543887471	4.76842145092875
O	7.74935776941861	-15.78305836485887	4.00172521714417
N	5.61602589509266	-15.36578112475630	4.55920894096996
H	4.93389100588536	-14.57837025266103	4.71282627053473
O	5.26568427972225	-15.95296054120017	3.34882726450976
H	6.11671899686455	-16.29482588953906	2.99644071631746
C	7.34585256267236	-14.40227639186007	5.94752969463779
H	7.95712578880201	-13.56504536975987	5.57590401157860
H	6.49023592586671	-14.01351003708775	6.51530636568597
H	7.98122952192714	-15.00937847471255	6.60935264011602
H	2.49526663120615	-13.25047447006069	6.01119729113658
H	6.09752738363753	-12.87933930669112	-2.57203693466625
H	9.83830084093712	-15.67455187276946	-0.41346954955892
H	0.17951139930505	-16.32989774131964	0.83820615891443
H	3.11851149055026	-10.16682958532529	-1.10861143041990
H	11.06607932056928	-10.71147703254279	3.77794196521493

$^{2}RS_{QMa}$

N	6.54998482596257	-10.89308358692580	0.34041515998079
H	7.31024073346888	-10.46535441244102	-0.17410600607806
C	5.59485956663718	-10.22511037952294	1.08499669931708
C	6.27746944047431	-12.22066260623605	0.38553318933620
H	6.87402882916362	-13.00801617687242	-0.07456874556588
N	5.20191021049731	-12.42589014521848	1.12412137147673
C	4.75934239252545	-11.20094553469156	1.56939049238433
H	3.88131679120270	-11.11872369721609	2.20416939955811
N	1.02588372993461	-13.84966551944719	-0.97639607126419
H	0.58086813417981	-13.94976675453808	-1.88134154062061
C	0.43674675125187	-13.34649793917071	0.16746111296573
C	2.29947058838578	-14.20063754893642	-0.68017201522739
H	3.01364426140030	-14.63285547508268	-1.37607728975528
N	2.55121500393228	-13.94610438401077	0.59294068212080
C	1.40346605588564	-13.41283761874737	1.13991111296893
H	1.37382816898299	-13.13078667099384	2.18791083531289
C	7.46776826283088	-17.34888643174207	-1.67394688218056
H	8.02475438938531	-17.64418876498484	-0.77170104714272
H	8.13023425275994	-16.71283115306181	-2.28689164994921
N	6.23230121720610	-16.68241981702911	-1.32794943175071
H	5.35933329637199	-17.00350285373698	-1.72589736237186
C	6.14070694626388	-15.66050028993169	-0.43220740954023
O	4.98620763615304	-15.19328804654637	-0.20392170176424
O	7.23434853829076	-15.23942250326542	0.08506673055467
N	9.56139954278716	-15.99017583771448	1.54183162809311
C	10.15783069796621	-15.89284243814601	0.30189453755430
C	10.41744645320337	-16.60254253850543	2.34430181455037
H	10.23083890109924	-16.82787408447634	3.39150067793749
N	11.54782256179822	-16.90431011768530	1.66352395993813
H	12.35163962574153	-17.39122243423251	2.04420459447985
C	11.40656533146838	-16.46143270650843	0.36292496413003
H	12.18190562570197	-16.59666149562691	-0.38582576799569
N	9.42308848808868	-11.54892886262177	2.73705471774815
H	10.17584630846842	-10.87913404254043	2.62630414217532
C	8.30135699491408	-11.38921168269724	3.52809027254052
C	9.37056389763786	-12.78128941452779	2.17331528970426
H	10.13183176417732	-13.18523420603627	1.50934436814075
N	8.27184403976210	-13.40516790079490	2.56020570398660
C	7.58913148779654	-12.55705783795016	3.40779796375758
H	6.64746874373991	-12.84763403478540	3.87036522581622
C	2.96461116797383	-12.96776087083463	5.52172552165705
H	3.36567601274058	-13.11941976107974	6.53161382276842
H	2.85501309127014	-11.88767679225966	5.33348442143735

C	3.87831729595605	-13.56713863597251	4.46743816512963
O	3.44008536360383	-13.56777317788911	3.27787097948167
O	5.00580675304739	-14.00813890176625	4.81618231107014
Ni	7.68960090152702	-15.30567917236137	2.08011604265349
Ni	4.37176029271850	-14.24257549863657	1.52090496523883
O	5.76924771512936	-15.20761487135985	2.56266950729335
H	5.60323596129643	-14.79611535266688	3.44955102529175
C	7.29604855441132	-16.02389802772866	4.90800221003209
O	8.18341572097824	-16.03344424299761	4.02404648191815
N	6.24066261343857	-16.86624697004794	4.87768337141345
H	5.46664853034368	-16.72561153538785	5.52271153192030
O	5.92169457863688	-17.52210669301325	3.71624370829723
N	7.34718756506960	-15.17793420699655	5.98084528145122
H	6.45838418927056	-14.72853432445604	6.20628256902353
H	8.12443195365975	-14.52878124761620	5.92607768240949
H	5.68231326451484	-16.76737514139473	3.09797761962225
H	1.96081985605730	-13.41104383836912	5.44301811295399
H	7.23613172872483	-18.25458344332213	-2.25087223943462
H	9.62701226600285	-15.44570607175176	-0.53303888407951
H	-0.59246062527462	-12.99999328631105	0.18772223924587
H	5.58949839044349	-9.14424372467703	1.19180042713856
H	8.11104250095971	-10.48149703590011	4.09323793074348

$^{2}RS_{QMb}$

N	5.16386702594449	-10.79480079431863	-0.46961316318669
H	5.73534977207407	-10.09624230703869	-0.92951929415136
C	3.78775603214458	-10.90842383910258	-0.55620632504417
C	5.61865109016414	-11.76376374979806	0.36150663389871
H	6.66033593161696	-11.93433533243751	0.61975219796413
N	4.60416112322060	-12.49231011046383	0.79762053265660
C	3.45505490709874	-11.97351387975896	0.24295515088776
H	2.47718817789941	-12.36800392969318	0.49714214467501
N	2.22821484740397	-16.92500863029404	0.35015608054727
H	2.07687403581325	-17.80091280567423	-0.13618482037222
C	1.24723637674453	-16.10397221581466	0.87021062147058
C	3.43339853346878	-16.36549495651904	0.61662501655133
H	4.39646219987143	-16.79208882600214	0.34517967234188
N	3.26797096595194	-15.23014993447342	1.27641511909449
C	1.91117338509270	-15.04902111621358	1.44762059358340
H	1.53717044955621	-14.17614289392517	1.98028701840676
C	6.66033338888470	-13.74505026820061	-2.41940100533993
H	6.53845158485934	-14.26315871386940	-3.38314878922271
H	7.72483331692136	-13.52937903498720	-2.26594551253227

N	6.20281245630874	-14.57847747911257	-1.32894528828050
H	5.25483512548587	-14.93465622601786	-1.34730588421327
C	6.83383505444602	-14.66958852828630	-0.13164409695732
O	6.25874247685398	-15.33447833514546	0.81299446524680
O	7.95456902516897	-14.11206784637089	0.05644603681619
N	9.22011562125285	-16.31016365727305	1.60456942658520
C	9.85429615583127	-16.49276295326058	0.39429212650155
C	9.44732629060801	-17.39006547994357	2.33514179449909
H	9.07537048766923	-17.55082264625793	3.34426360030640
N	10.20942138009489	-18.26560657485780	1.63641955373521
H	10.51581966095108	-19.17464716899995	1.96466986404766
C	10.47983735638698	-17.71549176225900	0.39904217530609
H	11.07414094590197	-18.23103118261959	-0.34994566383420
N	11.61559489889284	-12.76528235022572	3.35824855258112
H	12.61057426396464	-12.75091644523617	3.55271330815785
C	10.75762427185164	-11.68752863305875	3.44045608660258
C	10.90280379166867	-13.84584523754330	2.95678104838748
H	11.32677975098965	-14.83421606704636	2.79666537240520
N	9.63661665775362	-13.51157299730713	2.78221181113227
C	9.52435169901913	-12.17041228617114	3.07696243690130
H	8.56904179673788	-11.66024191395977	2.99250614551937
C	2.68275571166921	-12.35720958069438	5.38832515312385
H	3.56984840706735	-11.88725306894093	5.84075438771377
H	1.83485039511342	-11.66161105590705	5.41328641407913
C	2.98053940405266	-12.78516169087770	3.95823887005846
O	4.06886159557851	-13.48802989347711	3.82499825535763
O	2.22829824082460	-12.48659346780154	3.03727923771613
Ni	8.06934875192700	-14.66208701654257	2.11853636969353
Ni	4.84254157718529	-14.11695860959461	2.04414175242461
O	6.64614726299623	-13.38223635869395	2.62933853106813
H	6.59239509354380	-12.94438799334285	3.48654581772885
C	6.86988007563246	-15.24786045126865	4.80194383047849
O	7.72954462638403	-15.75514536251786	4.04598958148374
N	5.54090059908552	-15.48158858911767	4.61223998629060
H	4.88600141093323	-14.66506163180940	4.70809214578756
O	5.25149627053114	-16.00288847909782	3.33974439380267
H	6.10932149113231	-16.34819964117912	3.01537731067393
H	2.43639427380045	-13.24626533264339	5.99231000090225
H	6.11423478205561	-12.78616279169444	-2.46590636232159
H	9.79501095733790	-15.73558601156457	-0.38286130913811
H	0.18950142795770	-16.33693310391306	0.78800142336942
H	3.18269437983940	-10.22712118130184	-1.14722298260468
H	11.09272694772294	-10.69790543380645	3.73773674755404

N	7.20001256454939	-14.47266664248786	5.85982042997620
H	8.17896182526005	-14.41550741329112	6.10882243960569
H	6.50786219525036	-14.19594969889538	6.54545230150096

The Cartesian coordinates for stationary points concerning the AHA inhibition (**1**) process optimized at the UB3LYP-D3BJ/def2-SVP level

^{1}**TS1**$_{QM}$ (with 286*i* cm^{-1})

N	6.48058970338009	-10.78709673047553	-0.08177846261921
H	7.24006319413779	-10.43734512172524	-0.65287671723555
C	5.54206462817206	-10.01846780526054	0.58379622125806
C	6.19106765493835	-12.09529370832300	0.12784700025362
H	6.76615186149511	-12.94595933425911	-0.23874378920373
N	5.12133486394717	-12.19128412193395	0.89596067632324
C	4.69915847279159	-10.91484562541362	1.19179750100505
H	3.83176906456528	-10.74561958265777	1.82376288724993
N	0.96636998970666	-13.79803554313503	-1.08432808379496
H	0.53504797901386	-14.00617521881058	-1.97739195007840
C	0.36896739114632	-13.13679913262813	-0.02811276989330
C	2.22641617475861	-14.13312976563271	-0.71936312011361
H	2.94267087985387	-14.66958739426846	-1.33672622522429
N	2.46057121928744	-13.71750467823198	0.51386216338699
C	1.31711731359307	-13.09351949656766	0.96380252372695
H	1.27875297620131	-12.67439058517891	1.96494894288304
C	7.30589494091604	-17.72410569716837	-0.60321883524270
H	7.72304972999739	-17.78518438417937	0.41470625273697
H	8.08195209485216	-17.32121108455830	-1.27350487420334
N	6.10271477488608	-16.92048268398923	-0.62988043855236
H	5.21513527252881	-17.36349175503990	-0.82958000507409
C	6.02649966419929	-15.66446754268010	-0.11213344509228
O	4.87625053415690	-15.13583800276954	-0.03797058492349
O	7.12085372626621	-15.10290482321512	0.24737219196853
N	9.54229892208927	-15.60605536322733	1.76134307808494
C	10.07107250751347	-15.70682195624448	0.49256453782511
C	10.50328515940710	-15.91568413135232	2.61496357970898
H	10.40039971244938	-15.91618426511752	3.69755828476350
N	11.63886723740556	-16.21721847190494	1.94021282951629
H	12.51836920654863	-16.49836553923227	2.35900839739902
C	11.38702455960922	-16.09025917094011	0.58825625361794
H	12.14390062196860	-16.28836189367684	-0.16542273901624
N	9.18479999607476	-11.09518844306760	2.31727629083062

H	9.88093084141748	-10.40412144545586	2.06324358499369
C	8.12694913081374	-10.90312903181160	3.18593221069450
C	9.16389688034900	-12.38766197619911	1.90699476449854
H	9.88703558303210	-12.82606267466509	1.22269152632952
N	8.14613264077185	-13.01866838089058	2.46554708092760
C	7.48564882663148	-12.11414288777140	3.27028206790097
H	6.61108203575933	-12.39981877392794	3.85374732514134
C	3.01524863674930	-12.01641098299908	5.26914459188976
H	3.37904916814124	-12.12966818418823	6.29791389934602
H	3.12099012298737	-10.96227659233515	4.96422095887267
C	3.81072687715381	-12.88911306022767	4.31508766578282
O	3.33725845066490	-13.00583989502176	3.13759672754842
O	4.87507370270662	-13.41396125716884	4.72157662488595
Ni	7.62385421460336	-14.98876454908461	2.25031369176695
Ni	4.24957753174450	-13.92005898983584	1.51641696168491
O	5.61546271472328	-14.80634349591965	2.71098589275095
H	5.41897172179253	-14.35874080854584	3.59748499464682
C	7.43486811737349	-16.38500900825325	4.82399040026796
O	8.04255236622995	-15.43985118441965	4.27504495419856
N	6.64749560523741	-17.21693238311502	4.13816631536826
H	6.12616218896068	-17.96301149471729	4.58959370581927
O	6.40111380080191	-17.03859288964757	2.80822068537635
H	5.79438633965633	-16.06167548594097	2.77022478222005
C	7.55429048620157	-16.62532363106370	6.30274215810571
H	7.09991057668241	-15.76948808003688	6.82550486498191
H	7.06267240800375	-17.55123219150657	6.63212127259451
H	1.94562346343289	-12.26534910505753	5.21076998810146
H	7.06053012012811	-18.73970344871631	-0.94242185244263
H	9.45241589507448	-15.50674915056604	-0.37714658082295
H	-0.65209299843884	-12.76941123924505	-0.07450182323456
H	5.55439114248181	-8.93274125135520	0.55964188951501
H	7.93046600564071	-9.94706832951006	3.66194348358072
H	8.61793001463369	-16.65738300793431	6.57948533443763

^{1}Int$_{QM}$

N	6.50232420469130	-10.82984015763782	-0.02280313633940
H	7.30311601750381	-10.52026590402343	-0.55953713935747
C	5.55790400128349	-10.01462087607458	0.57496856264179
C	6.16986398330352	-12.12167240514083	0.21503021034635
H	6.73669869707934	-13.00277424770528	-0.09045585949418
N	5.06283306937843	-12.16408405963854	0.93561341998029
C	4.66276707141355	-10.86715187632708	1.17060586885857
H	3.76961329595566	-10.65147941078842	1.75015724999071

N	0.98290166132613	-13.74178312448591	-1.23868624210612
H	0.59239752027732	-13.98621513836841	-2.14145478982559
C	0.35891045132680	-12.99127255797083	-0.26057007236267
C	2.20283639580369	-14.10908568567148	-0.78163184302196
H	2.92529524671379	-14.71582329408367	-1.32206550193568
N	2.38608215989915	-13.62909543521072	0.43714130328144
C	1.25020843852767	-12.92882453067161	0.78154362444194
H	1.17119632542137	-12.45062782071195	1.75334553886229
C	7.22480937286851	-17.72233452898345	-0.41272813365147
H	7.62121014190120	-17.78106606283098	0.61318726059482
H	8.00129592893838	-17.30470365231727	-1.07217417125251
N	6.01242994681768	-16.93091866988729	-0.45461253383903
H	5.12652194128565	-17.40341417948247	-0.58230103141696
C	5.92247409632130	-15.67964864815793	0.06800275072054
O	4.74965310446781	-15.18750283955234	0.16593205294884
O	6.99683754233816	-15.07356804618091	0.39456811709221
N	9.50325966287888	-15.62873996866906	1.86770450378343
C	9.99341461332754	-15.72193756010946	0.58376010584489
C	10.49855249574543	-15.90628582474492	2.69121800675476
H	10.43244368085526	-15.88926048901588	3.77666104519403
N	11.62013557963311	-16.18415971792243	1.98187114164901
H	12.52032077428935	-16.43739772932784	2.37342246184936
C	11.32147231094418	-16.07184903857498	0.63820869963917
H	12.05821880286652	-16.25570828344991	-0.13873054974841
N	9.15307536954016	-11.11750386252165	2.31276496260872
H	9.86577081304781	-10.44755550509721	2.04816896194020
C	8.08024282623842	-10.87587230932326	3.14931069768569
C	9.13352830299616	-12.43138572849689	1.97236709728808
H	9.87129637179842	-12.90831652005880	1.33077082295110
N	8.10277294196865	-13.02952070626135	2.54137904775272
C	7.43241611243904	-12.07909503826693	3.28276405089150
H	6.54329531777655	-12.32144332471097	3.86124178305800
C	2.77888206517628	-11.77039572635076	5.15512253290742
H	3.08618338603286	-11.86388246702559	6.20389899246648
H	2.95517540194089	-10.73468473502290	4.82093480147654
C	3.57392137854285	-12.71193774419035	4.27479859904922
O	3.14669329585045	-12.88160967606391	3.08935329476155
O	4.60855886935580	-13.24853788179415	4.75076391330326
Ni	7.58321312761982	-15.04175309810444	2.43096973906782
Ni	4.10670184933393	-13.84380639175087	1.53555699176328
O	5.45906517222064	-14.64262635817979	2.90854420877751
H	5.16952660155280	-14.15881067324031	3.79692752575613
C	7.71073954165944	-16.57290298250831	4.81426868213470

O	8.08699734937536	-15.42355456019988	4.45489589984194
N	6.99380124716553	-17.33714180832550	3.99864829265619
H	6.68829896631828	-18.26992805094288	4.26428318381767
O	6.60797233227663	-16.90568676963487	2.77629510907663
H	5.47980431824494	-15.63669897954365	2.98740392942886
C	8.09055041137448	-17.13246936642893	6.15604661540901
H	7.70214319734421	-16.47068013516085	6.94504146298179
H	7.70803513729630	-18.14996757258786	6.31980465980732
H	1.70279253367014	-11.96903569176455	5.04875027973793
H	6.99460977781292	-18.73663839262170	-0.76654489175223
H	9.34362732174751	-15.53875336975251	-0.26671354587469
H	-0.63970494664612	-12.58361418562212	-0.38819639443232
H	5.60108574392909	-8.93099479542372	0.51672397741569
H	7.87817732252058	-9.89543779593527	3.57017976828786
H	9.18740764709437	-17.14248965336774	6.24990652783520

[1]Int$_{QM}$

N	6.24734621811934	-10.94357677979554	0.04351294086528
H	7.00567051140577	-10.60036273788162	-0.53334383507498
C	5.27698156364946	-10.17196293081819	0.65458578864507
C	6.00270702559346	-12.24633285777512	0.33039423217394
H	6.62072463294675	-13.08513423858195	0.01166726096536
N	4.92917219087124	-12.33926728572648	1.09479576030838
C	4.45945090811840	-11.06247525082357	1.30546553475431
H	3.56770425483262	-10.88072706962985	1.89786102317023
N	1.00308526487530	-13.80256569284166	-1.14858857777329
H	0.65562883854085	-13.98638691536194	-2.08242452045567
C	0.31082710790192	-13.17102529504542	-0.13419130415123
C	2.22424766836712	-14.15229351866536	-0.67505496565256
H	2.99226693873314	-14.67504272528302	-1.23847940851966
N	2.34399024503975	-13.77419541539587	0.58451066151416
C	1.16404119830485	-13.16039884298297	0.94199114539428
H	1.03053739108246	-12.76921847669257	1.94582114356912
C	7.20419876574268	-17.20761028651208	-1.62171893981801
H	7.78818097179412	-17.64628652153775	-0.79586211964104
H	7.85186392647048	-16.48310336399544	-2.14413847843591
N	5.98428384044149	-16.59304422565572	-1.14886023708596
H	5.08949491990891	-16.95624093737646	-1.45021755227952
C	5.94210998944973	-15.63773787254889	-0.17482035175279
O	4.80643000460264	-15.22781967629309	0.18607610057842
O	7.07464622989483	-15.21977190895676	0.26189202863413
N	9.41448652789941	-15.86541438947341	1.85624311244131
C	10.03878691636343	-16.06598669896497	0.64408387276388

C	10.20933239498503	-16.35016033038512	2.79756547721344
H	9.98145470425658	-16.34739294724841	3.86169241795167
N	11.32968134349665	-16.85734090656264	2.23185685068984
H	12.09254239823260	-17.30216360293009	2.73034186745848
C	11.24380428800221	-16.68785372837260	0.86366433721304
H	12.02381622520803	-17.02245414699750	0.18569799207379
N	9.39863364732019	-11.26026867824389	2.61325289555799
H	10.19316888763594	-10.63978760245114	2.50666066137720
C	8.20709373425098	-10.95572956226796	3.24280700093433
C	9.33923053791842	-12.54898527755287	2.19732658945809
H	10.14501871747478	-13.06400628383194	1.67873021302883
N	8.17094932563849	-13.07434433969600	2.52430472859927
C	7.44798263029882	-12.09740995151497	3.18010296665827
H	6.44329635979087	-12.26819785793021	3.55776410734832
C	2.22146119773538	-11.44800653269918	4.84920224037626
H	2.55197299836926	-10.95726234053206	5.77267828181511
H	2.04628712420890	-10.69090341025709	4.06813497459078
C	3.25141216505485	-12.45494013486027	4.36734206734713
O	2.94305356563791	-13.11688479009273	3.32103561038997
O	4.31718155221017	-12.58395798732917	5.00829626067668
Ni	7.56178082786405	-15.03107207238295	2.23898046820246
Ni	4.04348159416642	-14.08822865313552	1.76668329204805
O	3.09966770084450	-15.80775160744574	2.75792512380687
H	2.67023721196408	-15.22652007492865	3.41043797178771
C	6.79677516667720	-15.06805120758875	4.90752586424828
O	7.77557921630780	-15.55781887970351	4.27500381669381
N	5.70070791111209	-14.66828540701917	4.27592208003011
H	5.06734176325136	-13.89570374986011	4.65662816102157
O	5.69122754953533	-14.76418843332431	2.90285464818020
H	3.89689256646232	-16.13137736533295	3.20690852873185
C	6.81778471143847	-14.91522310106312	6.40009629890454
H	7.73675869547554	-14.38521986834747	6.69136159501126
H	5.94227558773089	-14.36293474657341	6.76722509777882
H	1.26042240513715	-11.95743640885596	5.01935935100098
H	6.94966399409688	-18.00780353047260	-2.33001898484299
H	9.56041562857367	-15.76443403001776	-0.28299986181342
H	-0.70148445178808	-12.79931064867507	-0.26375421335229
H	5.24851990943411	-9.09000621171300	0.56446839121531
H	8.00684432234360	-9.98004009308200	3.67570108569724
H	6.84694769069401	-15.91037494410082	6.87112928975310

$^{1}P_{QM}$

N	6.24719580033007	-10.89121071324073	-0.01403437983888

H	7.00806199407753	-10.52114419096420	-0.57052004249168
C	5.23720524813207	-10.15416912018033	0.57589849481385
C	6.04994841723524	-12.19893043174436	0.27631858225807
H	6.70796234118060	-13.01331672772593	-0.02424269727505
N	4.96507477045500	-12.32905686373274	1.02309918310679
C	4.44363168715909	-11.06968753007595	1.21906988565323
H	3.54694208351416	-10.90368811703487	1.80773624049173
N	1.10594549711415	-13.74586454661482	-1.02383624594464
H	0.79088492700521	-13.66898021242298	-1.98391306973526
C	0.30623401288059	-13.69240571124052	0.10141401626860
C	2.38559180528147	-13.93010361622676	-0.62562775291618
H	3.23860418553517	-14.03243074448767	-1.29090692333734
N	2.44307190047110	-13.99151672590824	0.69530523666666
C	1.15372821735141	-13.84419531524992	1.17083238116650
H	0.91818265750979	-13.90357430032937	2.23296632015298
C	7.39687452231242	-17.17256389803803	-1.65942866563212
H	7.95611877765187	-17.60392023826026	-0.81346495963036
H	8.06123720804850	-16.45724695996056	-2.17400426696358
N	6.16920929926258	-16.54842887539333	-1.21999662640933
H	5.28063408343769	-16.86972161769321	-1.58165307310864
C	6.10915284332724	-15.59084644548758	-0.25344982263379
O	4.96048102960713	-15.16025970089148	0.06018459234118
O	7.22508812307751	-15.18582201907044	0.22690496437430
N	9.53053037054988	-15.83421118674111	1.92930608295839
C	10.19613923244764	-16.06315523512718	0.74445260801932
C	10.30948951648545	-16.25891992792612	2.91199303633840
H	10.05366242380005	-16.21647578405853	3.96892753044121
N	11.45958585554177	-16.75505410514231	2.39940413666703
H	12.22017889860866	-17.15572249255500	2.93740286944007
C	11.41074970456928	-16.64093819493768	1.02383082465074
H	12.22004907516414	-16.97866314827640	0.38280314959356
N	9.48913668165370	-11.24394758991844	2.43780802115314
H	10.28094553084668	-10.62583938691644	2.30219660725210
C	8.31224732928116	-10.93015171587995	3.09160088848351
C	9.42342271933766	-12.53988955909618	2.04887373593124
H	10.21827713356763	-13.06337138325581	1.52223897116832
N	8.26330553163284	-13.05907486511988	2.41743992631594
C	7.55167880535594	-12.07332135117683	3.07358264640248
H	6.54486066562038	-12.22849693711781	3.46982401812549
C	2.37827830723496	-11.87445858984780	4.78183767136127
H	2.70082506822363	-11.07274303356759	5.45713877336745
H	1.74118313980268	-11.44903811392497	3.98814455269821
C	3.57951683298360	-12.53498512184819	4.13407220072336

O	3.31906754845755	-13.64180154972630	3.47705956728812
O	4.70154153369832	-12.04696596230649	4.22207653752383
Ni	7.67132247834019	-15.00822900863198	2.22155758414308
Ni	4.24884571699693	-14.11905225502229	1.68966538652588
O	0.89379948524415	-14.61176544005031	4.27449899700167
H	0.80178984534594	-15.55421245329490	4.08381520464399
C	6.69619848339346	-15.18937527271994	4.85022869561179
O	7.81467016870625	-15.39463089465686	4.30390031385670
N	5.61418785810140	-15.01790324008071	4.10750854710918
H	4.69915099677399	-14.68851447829563	4.42360983050400
O	5.73266484515400	-15.02715729563022	2.75121647291871
H	1.81139884953335	-14.38637903608930	4.00279518426788
C	6.53785892520263	-15.13540059419229	6.33994418774718
H	7.18587654352975	-14.33912573656068	6.73570332952005
H	5.50073793867004	-14.94034329564703	6.64431703546909
H	1.75609590000867	-12.61221331375874	5.30751119765947
H	7.15507682081333	-17.98049709453036	-2.36314436568791
H	9.73955009856592	-15.80730897550694	-0.20721752863471
H	-0.77060751197283	-13.56161790280104	0.04618825643439
H	5.17106326801973	-9.07410421504620	0.48431535605630
H	8.11993633034705	-9.94612759390646	3.50898471559451
H	6.87383753240407	-16.08768434713492	6.77736226197828

The Cartesian coordinates for stationary points concerning the HU inhibition (**2**) process optimized at the UB3LYP-D3BJ/def2-SVP level

^{2}TS1$_{QM}$ (with 433*i* cm^{-1})

N	6.50508333925994	-10.84055635462528	0.14678895173488
H	7.26717477998878	-10.46037471098229	-0.40081393963303
C	5.56613474581353	-10.10948621419466	0.85278541519749
C	6.21503386249420	-12.15815324872705	0.28354918530623
H	6.79080824158810	-12.98812555770880	-0.12703372933306
N	5.14415107092471	-12.29618900787862	1.04375745007519
C	4.72183770129882	-11.03772969826673	1.40881542022660
H	3.85259648934729	-10.90369584212953	2.04658572880098
N	0.98696735037118	-13.77875024495629	-1.04575103963208
H	0.55698656702016	-13.94297053634459	-1.94851329059797
C	0.39175807129497	-13.16017947671671	0.03726704253294
C	2.24294818360818	-14.14062400200539	-0.69228017489165
H	2.95657652350985	-14.65516771433858	-1.33087314231256

N	2.47662479250177	-13.78290431642504	0.55894242417354
C	1.33684853987738	-13.16996521595797	1.03294708760478
H	1.29837986539243	-12.79691768213191	2.05210443136927
C	7.33054652696816	-17.69621476364566	-0.83537929005657
H	7.73921339177025	-17.84755597997230	0.17659445260204
H	8.10856327212044	-17.22807111143683	-1.45897998869721
N	6.12182862254416	-16.90094988720857	-0.80022416078733
H	5.23628076477909	-17.33995624536594	-1.01722693871667
C	6.03496219729191	-15.69625255883277	-0.17290103964347
O	4.87764176556902	-15.19259379958154	-0.04559305404195
O	7.12377413734854	-15.15080988752697	0.22392454232815
N	9.55253852438518	-15.79088722688687	1.69583837429144
C	10.08156976521917	-15.80159763532525	0.42351915221678
C	10.50206921322955	-16.19681964437129	2.52121251746618
H	10.39569291370818	-16.28487448835250	3.59984894136411
N	11.63069794055273	-16.47374153640145	1.82404162419671
H	12.50067241625794	-16.81328203361638	2.21857076469859
C	11.38609818888786	-16.22845342005674	0.48718119927486
H	12.13907549750348	-16.38477759329770	-0.28010828421212
N	9.22933876539383	-11.30784529358469	2.49949892293586
H	9.93310097000074	-10.61102944082507	2.28550583301018
C	8.16602245126718	-11.15051819988641	3.36876104894518
C	9.20171537613912	-12.57858647034226	2.02589175294204
H	9.92718732667122	-12.98827338821972	1.32642310109206
N	8.17493690349000	-13.22825862705362	2.54469430486476
C	7.51496942275665	-12.35913907362450	3.38820353271852
H	6.63220440330510	-12.66523547468789	3.94878340870800
C	3.03312004373745	-12.26453058813720	5.36931779566212
H	3.36994090420101	-12.43958716703947	6.39879617152440
H	3.19278868574673	-11.20262557802209	5.11866773236993
C	3.81615500252983	-13.12353030521211	4.39188549469597
O	3.33658929129982	-13.20894584494500	3.21396778314536
O	4.87813860944275	-13.66392108781161	4.78145669947242
Ni	7.63677752863761	-15.18606447231703	2.23193438032534
Ni	4.25878401282736	-14.05259871255407	1.56150205471644
O	5.60627726477356	-15.00880920218701	2.73083699956979
H	5.41527951272089	-14.60134230675650	3.63675528670898
C	7.55901482797524	-16.80181201402711	4.66296259313040
O	8.09133467639959	-15.75441396623261	4.23272858357615
N	6.77305981839996	-17.57129949065026	3.88604625806468
H	6.31517134236246	-18.41626646145459	4.21039085073472
O	6.48568633175815	-17.19206568269965	2.59991642899057
N	7.81003991943070	-17.23725386470634	5.93423848551082

H	7.20140963831402	-17.92034530410677	6.36945213929792
H	8.20103517747120	-16.53694433148160	6.55357094600690
H	5.81306052961099	-16.22922041818230	2.70229600129192
H	1.95556410161953	-12.46303516231092	5.27752932239461
H	7.09544910104070	-18.67731943648053	-1.27020456936853
H	9.47034815380780	-15.51573331947312	-0.42727371388601
H	-0.62596534275252	-12.78220404037646	0.00454213470477
H	5.57881018251754	-9.02408757052622	0.88863734884469
H	7.97232309467633	-10.21724578881596	3.88905998439374

^{2}Int$_{QM}$

N	6.47100431533949	-10.80764080219761	-0.03023853090243
H	7.25105484797776	-10.47127060224601	-0.58129269946400
C	5.51057008216510	-10.02568871555553	0.58626713071794
C	6.18423423465287	-12.11051121129833	0.20927860655706
H	6.77100428491196	-12.97174730119026	-0.11436214807037
N	5.09226746649067	-12.19141235869272	0.94858628291994
C	4.65420533708414	-10.90839410733315	1.19464849224342
H	3.76764681076191	-10.72533833763492	1.79483929561722
N	0.97425162944372	-13.76375983690412	-1.13647797920751
H	0.56064101023433	-14.00356893626993	-2.03008727699785
C	0.37314445465879	-13.02332153537328	-0.13659485309674
C	2.20738797043706	-14.12982436291514	-0.71350348704954
H	2.91690200935738	-14.73055474984909	-1.27729031357208
N	2.42038377307914	-13.65758861506003	0.50281580978801
C	1.29145480935214	-12.96466988562941	0.88210905378006
H	1.23830518231663	-12.49355227526751	1.85926470488322
C	7.30841684407404	-17.66255280631080	-0.53749900023831
H	7.68538314831786	-17.82883652900030	0.48518200494586
H	8.08204242614594	-17.13535491618064	-1.11412384329091
N	6.07226385704167	-16.90865524070590	-0.51864838261705
H	5.19737505452939	-17.40364632581086	-0.63581811161397
C	5.96371833490564	-15.67164998426231	0.03042155721548
O	4.78106376175764	-15.21531534893625	0.17439337128245
O	7.02964633413508	-15.04157061486174	0.34334284761864
N	9.53469738311916	-15.64975581602897	1.81597845835126
C	10.03646767807949	-15.68915233929244	0.53414530513890
C	10.49450654419907	-16.05288472146780	2.62845788256797
H	10.41729992838510	-16.10733537678363	3.71196911638822
N	11.60546828695111	-16.35952744898047	1.91351047162908
H	12.48017788174554	-16.69974111697590	2.29646267297515
C	11.33581527297248	-16.13519790782634	0.57795865220570
H	12.06995907092031	-16.31596923992096	-0.20211900694173

N	9.10207802888119	-11.11884431143936	2.34357657954151
H	9.79392080430615	-10.42598479028192	2.08297647334371
C	8.03609749175504	-10.92163589803092	3.20076581162989
C	9.11131185251472	-12.42584434468408	1.97810120961664
H	9.85321247570020	-12.87203450274728	1.31931810386085
N	8.10482544029971	-13.06131274069497	2.54975777120926
C	7.42222020806178	-12.14406805099880	3.32070328630969
H	6.55049359724245	-12.42072815770307	3.91056289434264
C	2.92494143840445	-11.78759557485321	5.20453364447546
H	3.21655217922386	-11.90158914672573	6.25588196532941
H	3.16203635226914	-10.76194742759473	4.87662814980318
C	3.67530770603076	-12.76943844058047	4.32630568415851
O	3.25260025621171	-12.90146571306470	3.13450996846635
O	4.66950724332685	-13.36875980055408	4.81048476735045
Ni	7.62101675403694	-15.07522050245871	2.38853736728087
Ni	4.17383450766188	-13.88985365508082	1.56326193822880
O	5.46847046137336	-14.77045739894475	2.92749851215887
H	5.21521057344715	-14.31240963362518	3.82923653264275
C	7.76948937082460	-16.62734769209515	4.76710546739103
O	8.11432376331059	-15.46241971414557	4.43663199184839
N	7.14181004907929	-17.43119356922088	3.89489920978407
H	6.78496943639900	-18.34925565044696	4.13867989037705
O	6.73397155259715	-16.94132214756009	2.67998179703455
H	5.58649777740155	-15.76819968858253	2.95221281909067
N	8.09722925400318	-17.12011915845029	5.99686532435768
H	7.66807900306289	-17.95966124837193	6.36069814669438
H	8.43069173459912	-16.43582870025918	6.66201710919791
H	1.84018906200798	-11.92473693397397	5.08880351482083
H	7.12403651613706	-18.63126994908916	-1.02154142999713
H	9.41177012289906	-15.41521722949016	-0.31065639230448
H	-0.62991992126730	-12.61851098401197	-0.23519809535571
H	5.51800563859165	-8.94100955010498	0.53199892761883
H	7.81327194406377	-9.95451577737109	3.64151484592990

^{2}**P**$_{QM}$

N	6.22224677426553	-10.88592408410181	0.14690987904847
H	6.95680914083131	-10.47256184208607	-0.41454993478405
C	5.23441627579730	-10.19890161202054	0.82762983314810
C	6.04096133200838	-12.21249741987228	0.35047776420083
H	6.68912928437170	-13.00056093134600	-0.02991478564506
N	4.98864094442639	-12.40184086258369	1.12913563435077
C	4.47003718868337	-11.16317476100522	1.43456983395699

H	3.58803226001377	-11.04801150105674	2.05679875680971
N	1.16121500210543	-13.75059869397738	-1.04802239466248
H	0.85258314382957	-13.66947943883248	-2.00980638950589
C	0.36102698361405	-13.65585065296752	0.07390589092925
C	2.43075358733238	-13.98572336606403	-0.64441992222701
H	3.28158324087972	-14.12751273783959	-1.30493188082233
N	2.48214033032766	-14.04052857138976	0.67743482999823
C	1.19862791964817	-13.83539476890389	1.14688071173104
H	0.95551324452358	-13.87321375049940	2.20779661015421
C	7.44270707296296	-17.14487334067461	-1.74532799894549
H	7.97929568378723	-17.61516616488777	-0.90527618819486
H	8.12344661217577	-16.41553098140226	-2.21658489005847
N	6.21081167699004	-16.52588638736377	-1.30971892256439
H	5.32586400975209	-16.84660881619298	-1.68069084285459
C	6.13960168365914	-15.60030853018226	-0.31379718539138
O	4.98800321581605	-15.17759090709210	0.00018004416934
O	7.24813657328989	-15.20813360928716	0.19290055685132
N	9.50223922880176	-15.87881219135429	1.94584993269633
C	10.18526050605565	-16.10827550620303	0.77103570804722
C	10.28126212447367	-16.27010247212754	2.94186765720223
H	10.01565082699662	-16.21695846140371	3.99573225230711
N	11.44811839172322	-16.74584690233688	2.44792365278592
H	12.21215998626742	-17.12115417635788	2.99909654120381
C	11.41046747705895	-16.65263773342862	1.07058928986089
H	12.23385209815355	-16.98039736150998	0.44246318978958
N	9.33855745525113	-11.27030336029309	2.31535638318647
H	10.09048632471479	-10.62407737721906	2.10464398585183
C	8.24199644354385	-11.01195671810870	3.11690438425362
C	9.26752132164140	-12.56114982830415	1.90728268309464
H	10.00802453172675	-13.04668009552270	1.27526576043840
N	8.18122411649362	-13.12823259921847	2.40361138265329
C	7.52511319089537	-12.18194089551551	3.16589748792121
H	6.58474322388727	-12.38738030929517	3.68227258744998
C	2.49298557371499	-11.85457115642401	4.74886256800930
H	2.83057457077276	-11.09120260307365	5.46017498387183
H	2.01418396317047	-11.37496498311249	3.87959189101604
C	3.65816348980438	-12.70576146964812	4.28149644905286
O	3.34295620918294	-13.67318820170088	3.46299512267196
O	4.80518586354869	-12.49381407391514	4.68300719246003
Ni	7.62109040444713	-15.09425979408026	2.20724180872589
Ni	4.28077243442832	-14.22835820966501	1.68396318185917
O	0.85182883797171	-14.52192233223744	4.26081585514814
H	0.71507226041295	-15.46733503373318	4.11672405937248

C	6.64343997602481	-15.14318207842607	4.81709689850096
O	7.77753240669924	-15.35029988436288	4.31324854543517
N	5.52749323342708	-15.29631229493987	4.07599301796360
H	4.65362319146270	-14.84063731236447	4.34228496407583
O	5.68635408015762	-15.26913593215047	2.70258617092425
H	1.78134130835063	-14.35807042493046	3.99224207982940
H	1.72143151824135	-12.49554467394009	5.20101398100348
H	7.20979448331167	-17.92152728446223	-2.48620790770540
H	9.73183620229674	-15.87625529764817	-0.18817763691941
H	-0.70925313206799	-13.48067162805525	0.01443561900868
H	5.15893172398083	-9.11562969275924	0.81289935320513
H	8.07059220376319	-10.04345669534884	3.57742500814099
N	6.47479238253552	-14.81217979180416	6.12481341767967
H	7.33153403194769	-14.63466507872245	6.63503599170001
H	5.69675741363729	-14.19567026266531	6.34361101653494

The Cartesian coordinates for stationary points concerning the HU inhibition (**2′**) process optimized at the UB3LYP-D3BJ/def2-SVP level

2′R

N	5.77853009633164	-10.59309064935771	0.10855626829553
H	6.39139896077956	-10.05511339007169	-0.49208719324595
C	4.62588518349738	-10.13187219068020	0.71529565871570
C	5.95808723707909	-11.89071897524310	0.45722132279359
H	6.78969314670373	-12.52355488175686	0.14805839974384
N	4.97387846283506	-12.27637851675264	1.25168593392263
C	4.13542556585061	-11.19767287153017	1.42795139351569
H	3.25559513736804	-11.26973904713241	2.06100805509026
N	1.18640720258559	-15.16347992143547	0.27172109803060
H	0.68815662689751	-15.54392640919723	-0.52451710285821
C	0.61271314199569	-14.61696903110229	1.40341006774211
C	2.53289323599507	-15.10117134341114	0.40626266701979
H	3.25452349297471	-15.45476845041884	-0.32469300307899
N	2.84629374738149	-14.54561841734026	1.56492611529836
C	1.66233674736807	-14.23434897938874	2.20155696889083
H	1.67868154540585	-13.75420001853952	3.17547060078653
C	7.90719337349922	-16.19510582148127	-1.78631116214871
H	8.66649431488510	-16.71084810802333	-1.17361791413348
H	8.31364216115856	-15.21309087642710	-2.06966515651923
N	6.65065758759806	-16.04207851996791	-1.08467932291792
H	5.89585013448969	-16.68762662635708	-1.27732090860816
C	6.51629481310694	-15.28586916124865	0.03926174828522
O	5.41414487482277	-15.36371605268506	0.66994063716475

O	7.49880675101492	-14.53469019827217	0.35436215770352
N	9.93688160608177	-15.36746394974019	1.72352977340930
C	10.62715614097636	-15.16581734226454	0.54873330417386
C	10.52311295344619	-16.36821599790260	2.35510043634795
H	10.17733261285643	-16.79933874100298	3.29118557514784
N	11.58013990740051	-16.81401975527466	1.63534506212998
H	12.19209484686663	-17.57950896099468	1.89470820963278
C	11.66537820012277	-16.06290127762325	0.47960770336163
H	12.42945867963966	-16.23573481241403	-0.27285522641046
N	10.11127787585760	-10.87042858154039	2.48024453349020
H	10.93054161692204	-10.28876256767792	2.34681414867187
C	8.90407845769296	-10.46865074230501	3.01828296928008
C	10.02529717265622	-12.18970624654954	2.19048637057675
H	10.83881281385307	-12.77106607902214	1.76438248714325
N	8.82446845397745	-12.64815384519345	2.50990105667579
C	8.10800352161028	-11.58665914549788	3.03168962912539
H	7.09868466532827	-11.70557939090719	3.42229610833553
C	3.47666898302053	-11.72809214449873	5.66398364709522
H	2.69264226132980	-12.39981348484599	6.04402703037844
H	4.04155770061643	-11.29086165027535	6.49677960580799
C	4.40260452017658	-12.47656055492312	4.71834433710090
O	3.81940836901733	-13.19831941331291	3.83483273679858
O	5.63514477334346	-12.33898822832689	4.83703609253081
Ni	8.11112549931733	-14.57965795467288	2.27705985131255
Ni	4.75988728586939	-14.05174080628634	2.22718389769212
O	6.44601698659491	-14.27203650066510	3.21592165168531
H	6.36392415074489	-13.57210712794423	3.90820423516222
C	6.65787841803835	-16.86834932020874	3.25324375853491
O	7.56434034687100	-16.71548361495684	2.42698746609496
N	6.88434094764246	-16.79101193118899	4.62907462525884
N	5.39527274188282	-17.18073417285870	2.89467036993724
H	6.19979922501809	-16.18322677611199	5.08165978739807
H	5.15017709490587	-16.88647316810595	1.94656944302273
H	4.66036755034399	-17.25680933706549	3.58744345591432
O	8.15972904845363	-16.30409079657335	4.93509626288893
H	8.53619255385444	-16.98022297712066	5.51721078955706
H	2.97086875193255	-10.92071901902465	5.10916548698911
H	4.26701497897387	-9.11451120562054	0.58933428934589
H	-0.46338497314637	-14.55242637447572	1.53662729419393
H	7.73772033506111	-16.77731420527371	-2.70259342460474
H	10.30625872440146	-14.42030279353324	-0.17348190310881
H	8.72510966882272	-9.45161244839687	3.35483334243319

$^{2\prime}$**TS1** (286*i* cm^{-1})

N	5.08794456671285	-10.24978482679576	-0.01026938827804
H	5.56890428190464	-9.68238900252305	-0.69815249324509
C	4.05126582564380	-9.83541793546807	0.80428156217893
C	5.35283320678351	-11.55254623695937	0.26023186657851
H	6.14407398699536	-12.15106733300393	-0.19487139607004
N	4.53556468832100	-11.98260391734397	1.20707431101987
C	3.71920842310942	-10.93269967727179	1.56120315964376
H	2.96510642675361	-11.04542017499617	2.33550545272265
N	0.78526413265972	-14.83537740701545	0.15822745960971
H	0.24567891109001	-15.15342100724649	-0.63830316910430
C	0.27246186099496	-14.40425880441114	1.36653327008834
C	2.13709599180032	-14.76008733125553	0.21852790067291
H	2.82417905453530	-15.03089271815648	-0.57947562416325
N	2.50770845537961	-14.30470727894273	1.40222262478474
C	1.36313468805203	-14.07501679224807	2.13379528213421
H	1.42697726703561	-13.68759325824469	3.14675137926743
C	7.76232690785029	-15.91387794173420	-1.99062238453246
H	8.37010470409984	-15.01184805046765	-1.85343191832443
H	7.64871444627255	-16.10680063795461	-3.06897779752838
N	6.47563995775911	-15.69537185231932	-1.37455785995496
H	5.71997731112979	-16.34147202268932	-1.56523760797611
C	6.24744743072821	-14.81145782941116	-0.35709549313715
O	5.08737335467385	-14.80292477408355	0.14531195239620
O	7.21003245052872	-14.02915170977828	-0.02979063602732
N	9.69256449403480	-15.37107359597561	0.99309534615303
C	10.67218346950847	-14.98125990289055	0.10742938191182
C	9.76860805774232	-16.68689673827373	1.10811830855536
H	9.08668643810019	-17.29302322890689	1.70145564729801
N	10.77460418198688	-17.15850321848994	0.33351217888760
H	11.04155180581251	-18.13234847046345	0.24060499095055
C	11.36378154209205	-16.09110946374173	-0.31337612370933
H	12.19598682012230	-16.21241226194897	-1.00088273780558
N	10.36319349258100	-10.96337461028672	2.72942548014139
H	11.22920801788012	-10.49656394435393	2.97424422201084
C	9.10176443474739	-10.40463587377066	2.76239741980977
C	10.24432643777109	-12.25379379475137	2.33561979074910
H	11.08292902032417	-12.93970065083841	2.23808654417582
N	8.97370149928539	-12.54326407581078	2.11028847529975
C	8.24385597457628	-11.40374095765543	2.37509160496918
H	7.16076336353465	-11.39765252915659	2.29421893273608
C	3.55139439087137	-12.39988335031991	6.05656421946330
H	2.69785899766579	-13.06103220953943	6.27038881455193

H	4.18584338321837	-12.29950505546949	6.94576323944080
C	4.34450601744152	-12.94114801294061	4.87989702475723
O	3.68494590773820	-13.19839804002437	3.82578726816097
O	5.58686395470313	-13.09818702484494	5.01339278614704
Ni	8.08970082269906	-14.34922236180920	1.75283023664547
Ni	4.46205392828809	-13.91282795894188	1.97430422557140
O	6.39368513781389	-13.91569260621426	2.73893983609248
H	6.25138700323306	-13.44600058759519	3.62414221806632
C	6.38625661500293	-15.64397501037540	3.11184139778378
O	7.07457973380060	-16.12262819968307	2.15286511294847
N	6.98338442702234	-15.64900256583849	4.41164309279206
N	4.98288967857965	-15.90421615378630	3.10659929432623
H	6.52214403047679	-14.96578366789790	5.02563718162211
H	4.79053998479839	-16.74933756129298	2.57251487450619
H	4.54208167830069	-15.93021954591616	4.02461349260308
O	8.31278585854442	-15.15880291842635	4.26205129522326
H	8.86129729398961	-15.94352966871448	4.40183900343599
H	3.13581709222756	-11.41503165002577	5.79008393067073
H	3.65298720772805	-8.82535356231527	0.77343862946336
H	-0.79453847419414	-14.37209719262842	1.56718737725703
H	8.31072833489338	-16.76481292598822	-1.54387715102638
H	10.79792356041315	-13.94237051489139	-0.18592574288379
H	8.92894713382971	-9.37505805688367	3.06189987749164

$^{2\prime}$Int1

N	5.10666722738207	-10.24833389428709	0.17252707125546
H	5.62068680417524	-9.63756291362312	-0.45123662159999
C	3.99912600467753	-9.90011588893527	0.92151553655836
C	5.38986521962784	-11.55416225463912	0.40504141448937
H	6.21867436783084	-12.11716453849736	-0.02899097469563
N	4.51830747063207	-12.05162644810279	1.26717723984979
C	3.64519789452413	-11.03971130418862	1.60133819332576
H	2.83280828884853	-11.20457040356695	2.30354912979633
N	0.78172809484108	-14.87621801974691	0.18002474706883
H	0.23642434402074	-15.17990509062204	-0.61823910203503
C	0.27851090748151	-14.47725946940966	1.40266025570902
C	2.13379235200316	-14.79316579916645	0.22912290423126
H	2.81231876088995	-15.04168812520818	-0.58301332974857
N	2.51445130836798	-14.36307121805005	1.41878952054572
C	1.37518691812129	-14.15903646193939	2.16615840284946
H	1.44185949433053	-13.79680416660352	3.18796128674235
C	7.71424587418185	-15.84265098939906	-2.04184281439319
H	8.27894073173025	-14.90504396978434	-1.97911564365895

H	7.57300627023080	-16.10389706258722	-3.10176575155086
N	6.43620952006242	-15.65623866737575	-1.39600637361755
H	5.69771904204631	-16.32923574325007	-1.55968590637736
C	6.21645277122226	-14.78669354258742	-0.36679004852113
O	5.07624092794938	-14.81570134813855	0.17744424162167
O	7.16487363868880	-13.97707442863450	-0.06495721686939
N	9.70845452666966	-15.26402906163407	0.88897406711295
C	10.63941579372280	-14.93586666573814	-0.07089751088849
C	9.84053581824107	-16.55694857267235	1.13911650765122
H	9.20418146034737	-17.11364903430343	1.82528644112663
N	10.83555674347911	-17.07149634993146	0.37835436260628
H	11.13884347502079	-18.03908680800966	0.37980521300793
C	11.35885983447905	-16.05876391260989	-0.40019485727453
H	12.16991581331324	-16.22288710268153	-1.10385180656697
N	10.33306588841466	-10.79975232193693	2.47241183200448
H	11.20481514905755	-10.28918868821543	2.55777739315320
C	9.08425201439510	-10.34340848077064	2.84174113701657
C	10.19995150994132	-12.06130728569501	1.99960944625422
H	11.03169323420725	-12.67152305055362	1.65553201161697
N	8.92995585542910	-12.43083854395177	2.04115614799349
C	8.21777897012361	-11.37185729305666	2.56813956312941
H	7.14845188572165	-11.43210382212463	2.74917706321676
C	3.64615983292024	-11.83652404788934	5.70926771662257
H	3.49054878668485	-10.81903425213597	5.31297694195671
H	2.66092987668821	-12.27831555658232	5.90868182212689
C	4.39705486366298	-12.63697508508446	4.66765132510958
O	3.72407320940280	-13.26121846756426	3.80076048588492
O	5.66436326937545	-12.61262797007687	4.71131415676261
Ni	8.10467462770729	-14.26937731013702	1.67732467080896
Ni	4.46042709485181	-13.99657043853353	1.97097348006521
O	6.41555490078766	-13.95506325277838	2.81238194502834
H	6.19135342367190	-13.33689888574750	3.67048455757912
C	6.42728165435530	-15.42417271682049	3.14750434584428
O	7.23914487764221	-15.97977678101496	2.27661141062896
O	8.17874335825553	-15.11790008212282	4.64689322588713
H	8.68809485111112	-15.93241725751272	4.75456336492663
N	6.84046015432526	-15.59243026923165	4.53324120603536
N	5.01779295775133	-15.81800729793447	2.99137496421207
H	6.30942530176412	-14.95664372369245	5.13521857036413
H	4.99312220113153	-16.68959613524525	2.46318472786145
H	4.53834275927694	-15.95016151946278	3.88310413786205
H	4.23328478867373	-11.75244720592049	6.63301952687940
H	3.57231794747603	-8.90144295497276	0.90364156188576

H	-0.78641784234543	-14.45758343230314	1.61562408309889
H	8.32131193296473	-16.63574797521092	-1.56641304786835
H	10.71156846011260	-13.93178241074612	-0.48078580581783
H	8.92636635532112	-9.35805214904907	3.27036598411909

[2']**TS2** (413*i* cm^{-1})

N	-1.17573657243196	3.48904018971443	-1.55170858772536
H	-0.67449500626220	4.09212548905642	-2.19323634339516
C	-2.28420257659649	3.83826636656533	-0.80500731288205
C	-0.87113145711360	2.19362856798372	-1.29038950890536
H	-0.03852562459423	1.63043140600903	-1.71816345163606
N	-1.72990975407917	1.70277965343471	-0.41128796878449
C	-2.61729350193652	2.70925397568794	-0.09733402142977
H	-3.42918699342055	2.54970909016766	0.60682349110819
N	-5.45913747613453	-1.09345169048234	-1.49140254025796
H	-6.00542378265750	-1.39347194513539	-2.29041587019743
C	-5.96080028868184	-0.69924622751745	-0.26692215315652
C	-4.10677920285455	-1.01366319459593	-1.44271832634975
H	-3.42833720639751	-1.26116653943240	-2.25538737763682
N	-3.72376009595471	-0.58970076627811	-0.25162486634486
C	-4.86254015786737	-0.38695313319134	0.49693336408582
H	-4.79961911235239	-0.03136215316142	1.52126481219761
C	1.48310796113513	-2.07392094815348	-3.72208605349446
H	2.04865563499763	-1.13875001676282	-3.63687369161154
H	1.35302659266990	-2.31748591375917	-4.78781355479117
N	0.19953104946440	-1.89327123080538	-3.08550872315984
H	-0.53213490795752	-2.57408887139202	-3.24740175386448
C	-0.03151887263680	-1.01952456714511	-2.06256229292284
O	-1.17584560865091	-1.05430905271622	-1.52403549950196
O	0.90718450516486	-0.20188490233189	-1.75808446314997
N	3.45685746946884	-1.47132887607128	-0.79110177105489
C	4.39654764750992	-1.12518660720931	-1.73586065922050
C	3.58570560089650	-2.76881428998124	-0.56571110189006
H	2.93993092096238	-3.33640999293776	0.10299241247767
N	4.58700710317175	-3.26961532011674	-1.32819774983622
H	4.88937274037252	-4.23726172906199	-1.34319995441616
C	5.11844118428152	-2.24186386531215	-2.08133369341968
H	5.93577794213292	-2.39269963370066	-2.78069688499157
N	4.05901367948068	2.95420576009065	0.84653015456151
H	4.93331497185958	3.45170706295088	0.97244303320291
C	2.79925887068395	3.43925846686066	1.13285031483792
C	3.92997197344461	1.68519914207352	0.39110257213331
H	4.76866346146052	1.05361452920363	0.10757215566398

N	2.65368348229091	1.33834501623473	0.36589751274635
C	1.93239866752104	2.41914442755343	0.82947911335940
H	0.85370698518936	2.37705851087923	0.94530087893684
C	-2.48804623387201	2.01533470312574	4.02189454143436
H	-2.55197833199670	3.04649167483951	3.63593162756173
H	-3.50550589177722	1.65490394885257	4.21853557828877
C	-1.81488161919157	1.16524883489399	2.97525004939151
O	-2.50083894259756	0.52114766546018	2.15686495078881
O	-0.52697112079184	1.18088702151971	2.97814640173617
Ni	1.82721153353090	-0.50449271674670	0.00080875722159
Ni	-1.76220093609726	-0.24988708815865	0.28464330265780
O	0.15533376783338	-0.23131811374851	1.15202111482267
H	-0.12252319131980	0.52215007325585	2.13198041856109
C	0.17016607989138	-1.66783411893813	1.44225027545261
O	1.00220660880910	-2.23020304771413	0.58066020302138
O	1.88572508614058	-1.43697171491545	3.00727213613054
H	2.39508087368474	-2.25742649104307	3.04625001384081
N	0.55068307298705	-1.90614362917269	2.83595472958825
N	-1.24120187757819	-2.08419172837729	1.25597502246546
H	0.00697804291285	-1.29453787356620	3.44998584982727
H	-1.23983329481090	-2.92732015246831	0.68232057676495
H	-1.71054515815156	-2.29380436239714	2.13866884843040
H	-1.89328228388378	2.04137772060473	4.94449807291868
H	-2.72731393839360	4.82920051099606	-0.84485954164158
H	-7.02548515006937	-0.67877374650886	-0.05276492172895
H	2.08236131193529	-2.87616835161989	-3.25236441749350
H	4.47458979442713	-0.11265068945325	-2.12342248860303
H	2.63549155280044	4.43664548406558	1.53037525927757

$^{2\prime}$Int2

N	5.06313898758495	-10.28093840317620	0.13741087214894
H	5.56292611667581	-9.67713144737167	-0.50458373653394
C	3.95411946131572	-9.93363502322284	0.88418941737141
C	5.37064639146088	-11.57548037394744	0.39948755038561
H	6.20414515375236	-12.13746312576493	-0.02819058495260
N	4.51305734502315	-12.06776522703226	1.27899991386450
C	3.62352789876399	-11.06301891515629	1.59246351538840
H	2.81183773864902	-11.22374717240623	2.29658221245165
N	0.78637473342729	-14.85428636558806	0.19727561990194
H	0.23972130787802	-15.15099114478589	-0.60272775561871
C	0.28517243282316	-14.46210912865071	1.42259310280035
C	2.13889166187075	-14.77783657726864	0.24663037828605
H	2.81711193547614	-15.02472872444969	-0.56641382342526

N	2.52251021862125	-14.35788386520527	1.43897977397243
C	1.38395327967825	-14.15441448210986	2.18759999495768
H	1.44705059364613	-13.80170607559224	3.21293275767088
C	7.73120917763419	-15.84255460813430	-2.02862765129220
H	8.29704199782564	-14.90799225048712	-1.93901186235975
H	7.60190875824980	-16.08166807798531	-3.09549468195074
N	6.44750685862368	-15.66378388253141	-1.39186299898448
H	5.71617835381740	-16.34476819180590	-1.55452613558794
C	6.21424985539450	-14.78834951402053	-0.37101914522822
O	5.06880337358749	-14.82306244769907	0.16559065173883
O	7.15147271492903	-13.96932244816929	-0.06620292641112
N	9.70200843816393	-15.23668307980205	0.90091451062722
C	10.64274892778084	-14.88877184791212	-0.04216725881702
C	9.82916530767734	-16.53499895999300	1.12243080658778
H	9.18225952400894	-17.10370860263258	1.78913452573862
N	10.83035516561338	-17.03466098686965	0.35903811693845
H	11.13158891655445	-18.00260901213637	0.34127076952548
C	11.36358936780360	-16.00522930577573	-0.39054847614546
H	12.18125461493648	-16.15491767734193	-1.08977459004403
N	10.30203663093399	-10.81533898622988	2.54908352949397
H	11.17624773572741	-10.31934036174797	2.68135120857145
C	9.04106051032806	-10.32803675021566	2.82603613878324
C	10.17405300691021	-12.08402272030609	2.09248379565055
H	11.01362895057922	-12.71699916811572	1.81468136252095
N	8.89738856821172	-12.42863320451533	2.05783030606842
C	8.17462860450812	-11.34656286251002	2.51623207307396
H	7.09508848269058	-11.38716092801682	2.62397388040041
C	3.75748211822125	-11.74190676695588	5.70895357366477
H	3.70465312419467	-10.70988069910626	5.32370047461904
H	2.73625689933690	-12.09389086242304	5.90083980325869
C	4.42946751089058	-12.59678831843321	4.66562513095156
O	3.74513513891278	-13.24787106218443	3.85241436950841
O	5.71821412947531	-12.57692268750717	4.66677525614012
Ni	8.07183536592838	-14.27094634704599	1.69295756749247
Ni	4.48553573695630	-14.02076898819269	1.97381312418421
O	6.40020415345635	-13.99937278673161	2.84270651377222
H	6.12084262703977	-13.23711287612646	3.82820177718513
C	6.41675708592212	-15.43438374451733	3.13360991036894
O	7.24728712530552	-15.99849883933373	2.27143627568706
O	8.13450153912330	-15.19944229430588	4.69555144151977
H	8.64577392665562	-16.01868240981327	4.73499112393469
N	6.80012047174118	-15.67195232680936	4.52709449997387
N	5.00527894284866	-15.85213283283212	2.95009832773627

H	6.25612878339787	-15.06140622798043	5.14190722473765
H	5.00500942640484	-16.69828967290811	2.38102632186056
H	4.53390677556681	-16.05414264554888	3.83342452497385
H	4.34840999351596	-11.72168758097495	6.63425990183641
H	3.50909603237122	-8.94356835520720	0.84406085053408
H	-0.77952428016912	-14.43965921236419	1.63648016797335
H	8.32967823523533	-16.64702848765569	-1.56176329943957
H	10.72233875064144	-13.87508604038795	-0.42640594630062
H	8.87624614788975	-9.33030628797123	3.22226822625925

[2]′**TS3** (273*i* cm^{-1})

N	5.42540695035226	-10.23858833899128	0.50076452772909
H	6.02436768682033	-9.61991622386489	-0.03260702892458
C	4.29472941641027	-9.86945141658988	1.20350787749459
C	5.59674826525299	-11.57758219445287	0.63259944818088
H	6.40242459948504	-12.16676621467567	0.18809595200895
N	4.63157319725375	-12.07733757102540	1.38591286810878
C	3.81032211645156	-11.03328001156569	1.74998040464931
H	2.93045543155753	-11.19228515625032	2.36736530873703
N	0.80101769090370	-14.60046630416295	0.04733572522565
H	0.26056758273129	-14.80508512984534	-0.78507563864983
C	0.28996896968918	-14.29955106203296	1.29392099797789
C	2.15406429865916	-14.57928865776359	0.12579504139119
H	2.83953795189388	-14.78041268372683	-0.69377357522966
N	2.53036791513852	-14.28099789060927	1.35685378873808
C	1.38429491282568	-14.10152046502923	2.10039682356631
H	1.43460181550565	-13.84075258351397	3.15322080481472
C	7.55743104835946	-15.78889748536351	-2.31138287932408
H	8.09693832460541	-14.83858100248885	-2.41827670531392
H	7.37060870609098	-16.20199287450682	-3.31323208500934
N	6.29716549708149	-15.55780693464533	-1.64122120735046
H	5.52853151307353	-16.19485326473420	-1.80849368969216
C	6.17130595966206	-14.76708189629733	-0.53502753119718
O	5.05045774200106	-14.77586357126078	0.05380413440980
O	7.18266655938319	-14.05183013304327	-0.21284368903933
N	9.70035196498513	-15.33723396730465	0.89453355326258
C	10.55564787329399	-15.10230556027199	-0.15764631503296
C	10.05706287586958	-16.48617878068420	1.44294192765722
H	9.53364713591454	-16.95587337580206	2.27412468093037
N	11.12471594591263	-16.99758330235605	0.78410819176366
H	11.58964306545210	-17.87131139964381	1.00314630314866
C	11.45969390902960	-16.13403235526333	-0.23997851300956
H	12.28257519667375	-16.32922879649970	-0.92165084796761

N	10.03847734657669	-10.76860802335817	2.27556391722151
H	10.82170375474600	-10.13144938534631	2.18477848308884
C	8.92049469092473	-10.57732023611278	3.06414801663334
C	9.92170985874199	-11.97313580875080	1.66343003714909
H	10.67113843471873	-12.38825963681901	0.99325125363518
N	8.78884966035862	-12.55339769774691	2.01585742011021
C	8.14963856224020	-11.70092163823019	2.89242309121574
H	7.20070747558298	-11.98441433734330	3.33897676189912
C	3.70485401809806	-11.62487471016030	5.53540956304817
H	4.09004059839917	-11.54832279552985	6.55999435102204
H	3.99169563002601	-10.71200867200153	4.98697335680309
C	4.29659555944921	-12.80129193206576	4.81076425899670
O	3.71480261787835	-13.36335787032489	3.88717297953563
O	5.49227387632309	-13.14023163279839	5.23561563415638
Ni	8.00500607518322	-14.41686525815298	1.58528896369324
Ni	4.51465573351575	-14.07335590359466	1.91454784676631
O	6.36839375624759	-14.14412684885641	2.75021360351680
H	5.84306016920532	-13.91398115714088	4.71234237375416
C	6.36711439116431	-15.52648211203021	3.00441065419402
O	7.20889642465278	-16.15208287563457	2.18917429959891
O	8.04814835374461	-15.30959906696774	4.63236165501965
H	8.02174482389923	-14.58396648714714	5.27153660872097
N	6.67523764716637	-15.65989736183678	4.46079845687388
N	4.95437616985398	-15.97243757508686	2.82441801509328
H	6.63824754152622	-16.65460884922233	4.70162436272660
H	4.94015772693509	-16.81789610774073	2.25582993026460
H	4.45396415394647	-16.12433847874851	3.70104424295961
H	2.60976115385688	-11.69373555636364	5.53164654262203
H	3.94057171964399	-8.84332693321361	1.24341979888987
H	-0.77716088202231	-14.25577019989501	1.49176044170209
H	8.21311192816754	-16.48684480298572	-1.75938339348026
H	10.44540711999273	-14.23211628397189	-0.79988136116954
H	8.78230034093664	-9.68617224055489	3.66979738968453

$^{2\prime}$Int3

N	5.44711944281966	-10.21887628212492	0.11745634047360
H	5.97937839489954	-9.66060547813080	-0.53920220990111
C	4.37421604040116	-9.78523873593477	0.87200465619791
C	5.67074121808494	-11.52612405392391	0.40173202912975
H	6.47090897655618	-12.13397716891739	-0.02259913836335
N	4.79288480686913	-11.94389913319017	1.29888919079755
C	3.97940243282352	-10.87678682398747	1.60631444480573

H	3.18210875753201	-10.97023820023039	2.33773253813764
N	0.99952170137229	-14.80226839420712	0.30714326508892
H	0.47350691424851	-15.19642041305691	-0.46413458342823
C	0.46698496353008	-14.21907892611391	1.43863252389686
C	2.35101623104023	-14.75794626386772	0.40285406751015
H	3.05080351675919	-15.13621999117265	-0.33697806769343
N	2.70768219883609	-14.17887786210649	1.53588420961493
C	1.54732674106148	-13.83502265002689	2.19498824411172
H	1.57291146453196	-13.33516039094343	3.15754881444262
C	7.34977248754001	-15.32600578557003	-2.58752606311761
H	7.86364187965501	-14.35636482762020	-2.65046523858467
H	7.00551536007744	-15.60380543079762	-3.59386739163149
N	6.20903921899569	-15.22909085984547	-1.70350803209827
H	5.37669412510389	-15.76463962726442	-1.91235957087355
C	6.27277082806235	-14.68315089341884	-0.45151341833368
O	5.24704381140796	-14.78315152670477	0.28204297840756
O	7.36319752436245	-14.09087003921921	-0.14445670158172
N	9.72977295588525	-15.50278260740907	0.92954345042411
C	10.45273414498381	-15.38391475885907	-0.23609858854869
C	10.09814356348305	-16.63253694747775	1.50918357987479
H	9.67024065023731	-17.02007220436463	2.43218913059283
N	11.04619089558441	-17.24477432182305	0.75994376449116
H	11.49302936752366	-18.12789273607451	0.97985651616731
C	11.28805906058597	-16.46859552123703	-0.35617719554300
H	12.00798620927454	-16.75254295334438	-1.11844798428923
N	10.59037323728547	-10.96319456459963	1.96946797887069
H	11.47529083523495	-10.47239897539341	1.90709075189270
C	9.40696906750741	-10.44597119933896	2.45622360601161
C	10.37378753271078	-12.25302327949994	1.61333355432421
H	11.13921950661667	-12.91023097745067	1.20631046820870
N	9.11477770549089	-12.58119307532758	1.84357184752288
C	8.49376011059953	-11.46855409877381	2.37199846469171
H	7.44606281399365	-11.49755446329287	2.66020393838702
C	2.65769694347535	-12.55091651683127	5.88489410491141
H	1.85071748458273	-13.27765993463505	6.07460995197566
H	3.02920049582722	-12.21878015361038	6.86432746423663
C	3.75737292812201	-13.24076830054509	5.11193101256382
O	3.90623789388463	-13.01011743614868	3.90288717543122
O	4.45426970747919	-14.07180283089833	5.81235645443153
Ni	8.17417704617081	-14.39718891755097	1.66961541909634
Ni	4.69156976972262	-13.84441985504834	2.09215899956812
O	6.59506850915794	-13.86097672945409	2.84680721396630
H	5.36241406479496	-14.50288715858285	5.35646955022169

C	6.55366592003738	-15.16509078422426	3.34494681014356
O	7.53752344223694	-15.89214979856739	2.82863767288492
O	7.59702265562051	-14.05607322012514	5.17236487816068
H	7.43140745867835	-13.40779761223808	4.45194928994677
N	6.59898114546445	-15.01305837238206	4.84952687244205
N	5.19265031731499	-15.69809613738541	3.04221425059357
H	6.95273431824446	-15.89045272146323	5.24071788895744
H	5.29190293963831	-16.46160382302834	2.37415377448226
H	4.68649875626668	-16.02895435582213	3.86351168941095
H	2.25502585247031	-11.70353928492201	5.31660375165709
H	3.99770075100575	-8.76769762188792	0.82090601980439
H	-0.60333622806301	-14.13165444833974	1.60172051524630
H	8.08865101762327	-16.07569771233682	-2.25081925871406
H	10.30259613494672	-14.54725771099362	-0.91337477200342
H	9.32417388172820	-9.42577476033433	2.81987810049780

²′TS4 (107*i* cm⁻¹)

N	5.51153789642109	-10.25773609250284	0.46088976598979
H	6.09548908624321	-9.65078623564785	-0.10164925948059
C	4.42063516417643	-9.86656682482862	1.21364505110178
C	5.66792196286731	-11.59836064117102	0.59581252595434
H	6.45459029736234	-12.19611814843953	0.13268957481220
N	4.73139669614339	-12.07556664291566	1.39734714831291
C	3.94474254487939	-11.01779408483026	1.79270157032434
H	3.09956202739439	-11.16264115040924	2.45953191622999
N	0.82883332488134	-14.49362001340559	0.07444307193906
H	0.27283790759881	-14.67562844743714	-0.75288060399062
C	0.34098755007668	-14.18372748105085	1.32910876206939
C	2.18259617340392	-14.51534972289583	0.13852485226208
H	2.85081758156953	-14.73102834015326	-0.69115337015863
N	2.57905078120857	-14.23625622990501	1.36730058317657
C	1.44849740390376	-14.02421127362985	2.12571089302391
H	1.53377190385068	-13.77081588751582	3.17839861412276
C	7.55272287113699	-15.81806750141192	-2.31916135717988
H	8.10727667022811	-14.87917971455227	-2.45362184619360
H	7.33751760159992	-16.24285755595194	-3.30995294217620
N	6.30919008177919	-15.56494750521883	-1.62618839015156
H	5.49771492048930	-16.12977764467415	-1.84228556591349
C	6.22401138891438	-14.81936530350172	-0.48464826930165
O	5.11095059250721	-14.79029255894815	0.10987562122183
O	7.28248891472641	-14.18141501497646	-0.13982446808594

N	9.75900095857676	-15.41000036886294	0.93315410844636
C	10.55663574106078	-15.09274275153903	-0.14317793123788
C	10.13762963009407	-16.60103128603561	1.36552926179975
H	9.66537511333380	-17.14500758869843	2.18108136106936
N	11.16417565154423	-17.05904276214617	0.61056039426818
H	11.63608635062070	-17.94815129379898	0.73255881645710
C	11.44789470871893	-16.11604415472458	-0.35659675707277
H	12.23210640753934	-16.25630365061239	-1.09500514551171
N	9.97734621853148	-10.77325219139544	2.33692424851014
H	10.74336967169689	-10.11288280059607	2.26744361269113
C	8.81523207572566	-10.59861655408061	3.06341569833443
C	9.93471089207885	-11.99823526908764	1.76009482728155
H	10.73087093256712	-12.40505381992712	1.14066209382662
N	8.80507472470458	-12.60813222206870	2.07602331493657
C	8.09176827740844	-11.75234685707570	2.89300705213358
H	7.12937053602683	-12.04400921539688	3.30529313878192
C	3.55200411978098	-11.77388091170812	5.46968408772337
H	3.85362528156180	-11.61451533204678	6.51319036018405
H	3.75623003130039	-10.84397978771685	4.91242885990422
C	4.36608380558582	-12.89491204077178	4.83573253963307
O	3.81951777791202	-13.50595105869768	3.86027768716509
O	5.50183296054558	-13.12214781825765	5.30919550079758
Ni	8.09588082397567	-14.48548171103865	1.65684013853838
Ni	4.53737399358188	-14.03761745461335	1.96493079416488
O	6.41574612714350	-14.22088965229412	2.73184361266576
H	5.93948454509528	-15.33571438839457	5.35230960724787
C	6.37819553918322	-15.52419651344311	3.02560608292681
O	7.29597068943408	-16.23892701264813	2.46216440979193
O	7.67207653915789	-14.47585101662481	5.03606354866018
H	7.02917930967381	-13.70838100643462	5.04971549850445
N	6.77629572514734	-15.50030003070472	4.77490109342269
N	5.02269052262722	-16.02260590482154	2.98450297602656
H	7.22930823004648	-16.38417295911022	5.02232065388187
H	4.96884349393364	-16.99795509098729	2.70338458910084
H	4.44051911908636	-15.79862294509132	3.79004225347284
H	2.47370064723335	-11.97518744067918	5.40366151796849
H	4.08653005737254	-8.83428746669365	1.26466859347140
H	-0.72225140499831	-14.10722393822349	1.53766045807534
H	8.21051652140842	-16.51886672535064	-1.77313675172761
H	10.41353924133720	-14.17475736466154	-0.70706140900875
H	8.61532410928248	-9.69684223696465	3.63492161478541

2′P

N	6.23482040157319	-10.38182854142030	-0.51841087799541
H	6.61112006457947	-9.89893032276383	-1.32583942514462
C	6.04601187715147	-9.84133754756939	0.74148635919745
C	5.78345326421189	-11.66025680565502	-0.50574347956754
H	5.83479552442965	-12.35306063205766	-1.34239829174981
N	5.32477646904051	-11.95709502996490	0.69783327857874
C	5.47547040916001	-10.84116679540332	1.49056660397757
H	5.15957059520453	-10.86099164687796	2.52972467976132
N	0.64915520418965	-13.21028177424546	-0.28569601768692
H	-0.03418077829988	-13.35508574736050	-1.02014428882153
C	0.44666616892403	-12.50318610808483	0.88400211740597
C	1.91384055336190	-13.69219708578136	-0.28032520366345
H	2.36870647292942	-14.28657899918100	-1.06909018970083
N	2.52512388391692	-13.32777432048168	0.83485197247062
C	1.63023568082826	-12.58567789050304	1.57417243323330
H	1.91276915009660	-12.17558344485628	2.53950538587652
C	7.06173405028799	-17.19592121252026	-1.52429959319527
H	7.54687197808960	-16.73912360735772	-2.40010606560353
H	6.83900383175550	-18.24482536171044	-1.76168199785812
N	5.82275997303784	-16.51449943698508	-1.20362671530229
H	4.98817689243349	-17.05472565556268	-1.01184235640492
C	5.75034327474207	-15.19266244645163	-0.91241550054195
O	4.63680960911499	-14.71979606485030	-0.53140398378472
O	6.83033313265242	-14.50472319808541	-1.04328370430814
N	9.65966810120973	-14.14650517850883	-0.48354018365985
C	9.74671448364093	-14.29252539071705	-1.85187435556136
C	10.88739091682137	-14.24183952003870	0.00243468437899
H	11.16054976088477	-14.19148067677441	1.05437917031149
N	11.76681430159821	-14.43696977179500	-1.00725351030210
H	12.76835862925184	-14.55832863212352	-0.90342045138797
C	11.06378109940142	-14.47258053686854	-2.19506621462689
H	11.55017415379512	-14.62493339303311	-3.15424299748260
N	9.06714142152222	-10.75949711208550	2.74346961264183
H	9.48966443651544	-9.84014623922411	2.80185387147465
C	8.34013246965461	-11.38513319918842	3.73733150807105
C	9.11982877588990	-11.58799543550778	1.67517983902118
H	9.62903768642523	-11.35219156948635	0.74336060487492
N	8.46491085278761	-12.70919795129069	1.94000183001392
C	7.96366072326251	-12.60138572742429	3.22415366165172
H	7.37478821644781	-13.39444962635782	3.68902728009313
C	3.95956554311657	-13.34918478495732	5.52232272567645
H	2.90224689070344	-13.45380430536215	5.81584870164040
H	4.55059244950408	-13.97637980957589	6.20373509907671

C	4.11667699237046	-13.84675373128228	4.09884650268530
O	4.20530996166297	-12.96837199969990	3.18176373743360
O	4.12265680064780	-15.08168539088108	3.88227716575138
Ni	7.93713849784793	-14.14910587492384	0.59067449182080
Ni	4.47019246882519	-13.72306193346936	1.31586878409367
O	6.28379760910640	-14.58319858065395	1.75741344367647
H	7.78659780813071	-15.77235688654329	4.82240898326060
C	6.77365284671038	-15.76168302863268	1.99947796456535
O	7.96509358602073	-15.96624115155407	1.61272793795852
N	7.04440404784800	-15.16714086105664	5.18827470953325
N	6.00305372074623	-16.68677625210553	2.58194182891681
H	7.43165883588865	-14.77582952465970	6.05185724141216
O	6.01067295194673	-16.04730043742873	5.61559785055530
H	5.23689061354052	-15.76824134266151	5.07606321259854
H	6.43878516647148	-17.53433869752032	2.92236865176966
H	5.16076551739537	-16.32677628947415	3.04466579714542
H	4.24875353275944	-12.29451046630750	5.61439957930928
H	6.31120668370416	-8.81523538474798	0.97935739081175
H	-0.49779943057032	-12.01958886949709	1.11594858731362
H	7.76892613053567	-17.16296849099696	-0.67874102077528
H	8.85183800426187	-14.27878742088242	-2.46762686439917
H	8.15424690830299	-10.92040864896629	4.70124439948442

The Cartesian coordinates for stationary points concerning the HU inhibition (**2′**) process optimized at the GFN2-xTB level

2′R

N	4.53118428612032	-10.28020819558765	0.43971660001814
H	4.96773299058347	-9.47184860363867	0.02633690508117
C	3.26390006284882	-10.33301935006809	0.93952989631800
C	5.09308703968109	-11.48677913358769	0.63378091757939
H	6.09055897261313	-11.74507601711819	0.33340942538249
N	4.24867712824018	-12.30149668599146	1.22196259978131
C	3.10117898541769	-11.59728837928058	1.42070838285110
H	2.24902389734510	-12.03076459946459	1.90297663480613
N	1.24208601649211	-15.50095066322628	0.26600765246802
H	0.71178956195205	-15.75231329680032	-0.55307294278405
C	0.74576191038308	-15.37718265892515	1.53116462071834
C	2.55698162037025	-15.21869428795743	0.31857702864621
H	3.21952846266861	-15.23373507118387	-0.52302454528852
N	2.92235070393726	-14.92816776226445	1.54424563159109
C	1.80289626198112	-15.02102446453360	2.31487350365609

H	1.83719673421292	-14.81954507916376	3.36620856926538
C	7.98650146879336	-15.26079627491245	-2.40808680006219
H	8.13704324323577	-16.31918795161543	-2.63844070189245
H	8.82839188448043	-14.90237502484310	-1.82069322010578
N	6.79212362660241	-15.05185314767166	-1.62862164498936
H	5.92139838598189	-15.38124591104905	-2.01390108840868
C	6.79306666091055	-14.78749323530668	-0.27999158645204
O	5.67226355349353	-14.84116098022031	0.27505351747045
O	7.91143472786895	-14.48099832051716	0.22069414508059
N	10.11431345654726	-15.72214887319945	2.25263075384943
C	10.82768805303409	-16.23782272347958	1.21389747498777
C	10.40931455626644	-16.44822103167712	3.30702799194961
H	10.00793383136005	-16.29728650499992	4.29039664805843
N	11.29339451323816	-17.40956912440256	2.98909940914682
H	11.67696276617777	-18.10143925271763	3.61324229696537
C	11.56948675412686	-17.29153869539019	1.65879089707214
H	12.24810315567852	-17.94427823714650	1.15103964631763
N	10.80256147967876	-11.09789239389500	1.64359781413026
H	11.29977977487237	-10.44285083130829	1.06118530133904
C	10.75769127975730	-11.07871709244569	3.00649862196569
C	10.07463533925028	-12.15002533184341	1.22840147731223
H	9.92275868093489	-12.42118812137963	0.20254105124637
N	9.57094394684793	-12.79748746360660	2.25254912244713
C	9.98677515121419	-12.14192326013811	3.37313269714157
H	9.70969079474017	-12.47209833859716	4.35435928605376
C	3.92140634248686	-12.61254261395391	6.00715151809611
H	4.80895971811569	-12.71181851406156	6.62926393788716
H	3.50660510462744	-11.62010765650848	6.15426324291759
C	4.33125254378965	-12.77666822955370	4.54369128547140
O	4.16852341180638	-13.94729092698867	4.06437939692304
O	4.80572570313401	-11.83215149557943	3.92242909118528
Ni	8.41545933406064	-14.52425868429226	2.18691906641542
Ni	4.81334428483414	-14.10013029361612	2.05814678571135
O	6.70616511469241	-13.41146675292598	2.69211981964817
H	6.69351882580949	-12.45133606851133	2.72145792937719
C	6.36686927715444	-16.14805686829152	3.21819528101258
O	7.33396319246126	-16.31460789537761	2.47774932694188
O	7.57277455405437	-14.77206235314005	4.65973498505440
H	7.29742370163227	-13.95519881766202	4.11785508458553
N	6.49091596971686	-15.64492065146586	4.48700387633829
N	5.10518523672798	-16.51000139461173	2.85274670128943
H	5.63716979243920	-15.24443813557602	4.88051401875776
H	5.01503441540266	-17.05457723186921	2.00862387220888

H	4.36019487418604	-16.53659910106370	3.53148558845162
H	3.19956408340126	-13.37348576745308	6.28891302153581
H	2.60042634832448	-9.49416577934645	0.91902268675986
H	-0.28386160193089	-15.54815211763236	1.76556878977303
H	7.92814094010854	-14.69941789004208	-3.34224772689736
H	10.75558927923474	-15.82271501658738	0.22945226816426
H	11.25842684379092	-10.33525927273512	3.59021976167721

$^{2\prime}$TS1 (278*i* cm^{-1})

N	5.084281	-10.246842	0.021235
H	5.581580	-9.681751	-0.650364
C	4.035524	-9.835042	0.799364
C	5.326075	-11.542829	0.304874
H	6.117008	-12.135825	-0.127120
N	4.485927	-11.970046	1.222420
C	3.673904	-10.921029	1.544163
H	2.903127	-11.022042	2.286067
N	0.749969	-14.845543	0.188752
H	0.206711	-15.157461	-0.602178
C	0.249147	-14.423222	1.390755
C	2.093743	-14.764807	0.254353
H	2.767841	-15.027395	-0.541069
N	2.471236	-14.316851	1.430852
C	1.333702	-14.097365	2.153481
H	1.386876	-13.718639	3.157307
C	7.690772	-15.954378	-1.995715
H	8.336299	-15.093806	-1.818928
H	7.562872	-16.098008	-3.071651
N	6.419607	-15.690846	-1.373767
H	5.648733	-16.309350	-1.574138
C	6.239625	-14.810897	-0.339109
O	5.084097	-14.778199	0.153727
O	7.224490	-14.064816	-0.040685
N	9.769069	-15.398844	1.008130
C	10.738323	-15.003536	0.129424
C	9.859597	-16.708698	1.102297
H	9.206557	-17.328290	1.691785
N	10.855254	-17.171943	0.321967
H	11.130316	-18.137105	0.214410
C	11.426416	-16.100249	-0.305991
H	12.247975	-16.201702	-0.989629
N	10.438162	-10.968841	2.760863
H	11.293394	-10.502590	3.025119

C	9.187475	-10.416673	2.767677
C	10.317937	-12.249161	2.358898
H	11.148072	-12.927757	2.272491
N	9.059893	-12.539388	2.106435
C	8.339087	-11.404833	2.357660
H	7.269541	-11.389524	2.244433
C	3.446941	-12.419126	6.040467
H	2.589971	-13.067688	6.224110
H	4.052038	-12.331516	6.940776
C	4.285275	-12.987261	4.897685
O	3.658724	-13.214997	3.825930
O	5.496834	-13.192279	5.090941
Ni	8.134836	-14.348754	1.749973
Ni	4.443757	-13.899634	1.981549
O	6.396427	-13.798024	2.688021
H	6.311732	-13.359263	3.564275
C	6.395271	-15.677289	3.093297
O	7.083706	-16.127527	2.149788
N	6.992333	-15.569095	4.369896
N	5.002097	-15.883346	3.095055
H	6.518949	-14.871086	4.957871
H	4.735957	-16.652530	2.490341
H	4.561984	-15.907000	4.007829
O	8.328029	-15.093681	4.198281
H	8.890111	-15.871020	4.327258
H	3.070912	-11.436848	5.752025
H	3.644848	-8.835671	0.760858
H	-0.803956	-14.391757	1.597135
H	8.173133	-16.846273	-1.574164
H	10.863205	-13.972203	-0.145842
H	9.007468	-9.399671	3.060683

[2']Int1

N	5.07780873431333	-10.26691715836452	-0.07942639832375
H	5.69223509612604	-9.68957473565877	-0.63094543816606
C	3.83689927902632	-9.91656375192233	0.36850529080424
C	5.32353052766572	-11.52252938198350	0.33595981965686
H	6.22368661139358	-12.07538894614826	0.12803355863527
N	4.30225138143093	-11.98437010501496	1.02251944360178
C	3.36546017829003	-10.99649724759905	1.05373833235228
H	2.43491716607276	-11.12552930400034	1.56796119577232
N	0.69304555761207	-15.29470625436858	0.43725871173626
H	0.14352307406742	-15.73618552460134	-0.28288057246216

C	0.22225196852184	-14.83801166316809	1.63406822251623
C	2.01381872357432	-15.04476561555303	0.39853184448960
H	2.66201263758554	-15.28755235814861	-0.41965628222205
N	2.40494549839300	-14.45741845075394	1.50551639851326
C	1.29933468130403	-14.31905318549202	2.28869320161282
H	1.36134811071555	-13.85692212136727	3.25259319812217
C	7.63566406127318	-15.80989876495392	-2.08268243012613
H	8.38087124054631	-15.15252646895146	-1.63814146739591
H	7.49735881107827	-15.53772263173833	-3.13246600218667
N	6.41599073864632	-15.64171665398237	-1.34110643478913
H	5.61415975900786	-16.19176143512418	-1.60335912040138
C	6.23894963269053	-14.74176496783612	-0.32357369173456
O	5.07322880537151	-14.68897933665203	0.13861314807172
O	7.22535659132953	-14.00238070530102	-0.02669275410088
N	9.81264198966115	-15.36462390688776	0.89910268234380
C	10.89309738403398	-15.00563947464691	0.14906509639946
C	9.66030441178133	-16.66205661430524	0.73931110253522
H	8.87631304509940	-17.23915779265298	1.19173446715321
N	10.60665223183220	-17.15201222233886	-0.07988906071838
H	10.71634956580759	-18.11346382150417	-0.36121492764619
C	11.39969497854113	-16.11211401792887	-0.46559487738747
H	12.23434407101628	-16.23508808084360	-1.12329871171343
N	10.44968962862040	-11.05726541730963	3.30811376368955
H	11.25550244083110	-10.67765928928382	3.77911350984729
C	9.23028764754815	-10.45836888703384	3.20162123788053
C	10.36923009951923	-12.25037610683787	2.69292696452152
H	11.18685856272183	-12.94053497681032	2.62535260096238
N	9.16812297774148	-12.44231993045217	2.19641542218636
C	8.44279454346063	-11.32854965673197	2.50851760645604
H	7.41355371194145	-11.23148266048990	2.22535584121441
C	3.62941100206557	-11.76244130349184	5.79118915907872
H	3.66944509988686	-10.68916516519103	5.61752560121061
H	2.59249677869180	-12.08246038591764	5.81188708115189
C	4.37408005115725	-12.45545953575060	4.65809845697526
O	3.67784100947367	-12.91683730545911	3.72663440907227
O	5.62211449909882	-12.51432920630121	4.73344358940866
Ni	8.26672968671878	-14.26843649442715	1.71540891633034
Ni	4.31768054010866	-13.85120995652615	1.92630165050215
O	6.39467723759539	-13.73898661945536	2.66515824067335
H	6.17696663029242	-13.16240690704607	3.52336213415071
C	6.42706863591930	-15.14346493067117	3.06297443860690
O	7.07565992766659	-15.85503134470578	2.17234734846101
O	8.50091290745295	-14.88764581392018	4.09235168736025

H	8.93737663389904	-15.75094247076946	4.03496104945474
N	7.12637369627339	-15.20711506817548	4.36861665961233
N	5.02790050676439	-15.56517514680533	3.20885436063500
H	6.80374046503512	-14.45140704086789	4.97691240609390
H	4.92056345235085	-16.48005795152433	2.78039140340552
H	4.73676757203166	-15.60409808672251	4.18152591361146
H	4.11810482851330	-11.97732962777254	6.73719307297459
H	3.40248576815766	-8.95869768501321	0.17267470474954
H	-0.80664611051206	-14.91751662221463	1.91609419582782
H	7.97930165464586	-16.84586589959710	-2.02942145672867
H	11.22755305288618	-13.98980534806348	0.09322939127505
H	9.02231016763238	-9.49507037886712	3.61735962440593

2′TS2 (624*i* cm−1)

N	5.10756899405310	-10.27870664520196	-0.06988599066265
H	5.72933596085798	-9.70784231657437	-0.62016818711187
C	3.86681649718793	-9.91753594848803	0.37035645889979
C	5.34132452175148	-11.53529154989372	0.34909007750153
H	6.23849787440420	-12.09675597797231	0.14982786076044
N	4.31304272472515	-11.98758741424714	1.03166619710822
C	3.38360144290198	-10.99244164557051	1.05535487256292
H	2.44813165849351	-11.11267262303525	1.56292824398294
N	0.68490464642030	-15.26218821722134	0.41165427636316
H	0.13043035419772	-15.66967489128274	-0.32449567444866
C	0.22912063916226	-14.89277322083566	1.64370099845030
C	1.99949336573382	-14.98251903894822	0.36477806244490
H	2.63582270125638	-15.16028944820281	-0.47898021343650
N	2.40137752934293	-14.45875877302847	1.49930544488207
C	1.30886172910012	-14.39421128504501	2.30984146320458
H	1.37791834179775	-13.99185288260112	3.29961083355910
C	7.61596183740052	-15.88656909726286	-2.04476302490011
H	8.35884227886555	-15.20723077267808	-1.63044642073162
H	7.48437342922958	-15.66901753790364	-3.10810640811381
N	6.39131837707553	-15.67856797029441	-1.32219081592591
H	5.59477576563690	-16.25057362845274	-1.55121774766399
C	6.22029738534328	-14.75520245779898	-0.32577972745009
O	5.05135239101710	-14.68735106549157	0.13210233017550
O	7.20501808532463	-14.00890905795119	-0.04869484666035
N	9.80983115002856	-15.33137302743367	0.90419841334609
C	10.87539550447284	-14.94723591976284	0.14576695535493
C	9.68185251149434	-16.63023149830238	0.73749296996032
H	8.91286043927411	-17.22542373677254	1.19195946149574
N	10.62898313316701	-17.09711081198319	-0.09455687437594

H	10.75340986127822	-18.05409172923005	-0.38539777764706
C	11.39762798469043	-16.03932604737718	-0.48155663878991
H	12.22757629463978	-16.14200196790493	-1.14826535909949
N	10.40153980280695	-11.06579489722906	3.36109866786849
H	11.20400955749835	-10.69317998036274	3.84322214553296
C	9.18005324966406	-10.46941077937162	3.26132170909784
C	10.32791945813033	-12.24614080221044	2.72027534783190
H	11.14840087962898	-12.93172115402874	2.64246095149604
N	9.13030021401083	-12.43175296104890	2.21370513350151
C	8.39951280863050	-11.32760479167696	2.54560487148041
H	7.37064680212626	-11.23219012665741	2.26097072459543
C	3.71428775879906	-11.66905995228487	5.76274368331159
H	3.84109688220187	-10.60080178077219	5.59787060368692
H	2.65535044783672	-11.90459452160447	5.78924498656645
C	4.39009961514200	-12.41174493391545	4.62745446714506
O	3.68378458686801	-12.91119638061847	3.74004284975957
O	5.65772152106601	-12.46794274508873	4.67049272677582
Ni	8.23401353926569	-14.25805615382649	1.70789271079160
Ni	4.32333633287578	-13.86546959622796	1.91995737072181
O	6.37981564436741	-13.76544005472956	2.67892224975206
H	6.10106063821456	-13.09539536046530	3.65339358948636
C	6.41338749956302	-15.14789538091835	3.03895876368040
O	7.06105654017779	-15.86083426764496	2.14040507489234
O	8.49683551399208	-14.94376895035811	4.06822227525342
H	8.91256924621711	-15.81095595145395	3.94631156786170
N	7.12129779632779	-15.25569632932948	4.34647391841628
N	5.01335532616274	-15.58908994020251	3.18651498059271
H	6.81723202173268	-14.50943426416687	4.97349494025807
H	4.92011411885092	-16.49647479696182	2.73833172987212
H	4.73584477266024	-15.66057350492389	4.16154113526539
H	4.18860144611991	-11.92535653621800	6.70575607103692
H	3.44037121801631	-8.95720247668193	0.16900961751171
H	-0.79229148343077	-15.01133612664712	1.93911344164405
H	7.95952899762327	-16.91824940002808	-1.93553771999707
H	11.18982681834366	-13.92475960660454	0.09380461142608
H	8.96750285818492	-9.51524390099201	3.69542272984944

$^{2\prime}$Int2

N	5.11783861141828	-10.32222664147817	-0.11110857521913
H	5.72739894425128	-9.77086848654847	-0.69370332037375
C	3.88061746706416	-9.95227460722281	0.33134283521559
C	5.36530886648732	-11.56071971731385	0.35189698573153
H	6.26233485782478	-12.12594939047111	0.16170178921312

N	4.34998733940861	-11.99338895778782	1.06579715824323
C	3.41415189685868	-11.00336170897675	1.06334067564099
H	2.48441454278854	-11.11025005328765	1.58440987282052
N	0.68750895859248	-15.24337712131626	0.43654585790896
H	0.12813163512425	-15.63509594334490	-0.30433998581870
C	0.24073203254813	-14.90133203700645	1.67967598933888
C	2.00188278765008	-14.96166215966478	0.38687084324102
H	2.63213711434390	-15.12117759461450	-0.46510332883566
N	2.41264316585741	-14.46230501832218	1.52872463666235
C	1.32554438457715	-14.41668392872653	2.34789567507206
H	1.39973236235668	-14.03850777678303	3.34664239701212
C	7.58128949141028	-15.92592581296564	-2.05701259407222
H	8.33352010163990	-15.25424402915565	-1.64707739906226
H	7.44708007027452	-15.70828882707567	-3.12005523851139
N	6.36304882379300	-15.70278210959804	-1.32800991859414
H	5.56248550393776	-16.27528032842388	-1.54070530485057
C	6.20894301168033	-14.76921177991568	-0.33886141132267
O	5.04232798140286	-14.68942881667163	0.12805599129101
O	7.19889451136060	-14.02706930361335	-0.07848241855333
N	9.81468693676327	-15.30217773022177	0.91267077549783
C	10.88726691035410	-14.89147804238447	0.17873305744598
C	9.69693504489623	-16.59588095498248	0.70771108424292
H	8.92609596757661	-17.20818727889327	1.13550361513256
N	10.65738854666073	-17.03493895649260	-0.12531594681414
H	10.79084886082112	-17.98249482334764	-0.44069939197961
C	11.42435742943633	-15.96207061405708	-0.47245470482244
H	12.26339456836389	-16.04141879581698	-1.13127004068820
N	10.32419827182441	-11.10494988370824	3.46922094125529
H	11.11335523461871	-10.75426351261844	3.98818221866963
C	9.11696999301641	-10.48688883712448	3.33274778670246
C	10.25670446997164	-12.27262440682403	2.80382588519114
H	11.06852691815571	-12.97026953251051	2.74502069628106
N	9.07754574659079	-12.42939292977496	2.24761895819235
C	8.35221754095629	-11.31965540293694	2.57108888318874
H	7.33756444380299	-11.20380947875286	2.24653070143218
C	3.78369714317766	-11.53921937276283	5.73604107904887
H	3.89737733921995	-10.48148563825961	5.50713543000683
H	2.72810775089176	-11.77328022848231	5.82929700381994
C	4.39896777771904	-12.34136646638971	4.61844071003677
O	3.70267940459157	-12.91342116257801	3.78959015631895
O	5.69575494897714	-12.36161917292983	4.62504116044599
Ni	8.19444583322209	-14.26058858854235	1.70283263944413
Ni	4.36011424921384	-13.88949203565358	1.93192458680110

O	6.37133005786979	-13.82640429940836	2.67194665560055
H	6.05967179951501	-12.93966092411289	3.80347071251707
C	6.40715382276505	-15.18405536787302	2.99771153797618
O	7.06570189359083	-15.90239741547946	2.10584085462210
O	8.48763475711926	-15.00636953558918	4.04750630733832
H	8.87869798432414	-15.87095199094977	3.84626704515828
N	7.11016867261310	-15.31319323595556	4.31769357937954
N	5.00602787088980	-15.64186254652654	3.14546012844848
H	6.81226048248114	-14.56714459198428	4.94641966886628
H	4.91958458283838	-16.53363503313328	2.66481328845441
H	4.74509184680721	-15.75885969134302	4.12076055768176
H	4.30486377605798	-11.73934602004223	6.66789576822864
H	3.44453752837456	-9.00340168374803	0.09873377911737
H	-0.77827968305487	-15.02729691496604	1.98011192270062
H	7.91375302430970	-16.96120934898979	-1.94746263560343
H	11.19610630687213	-13.86632850530818	0.15981042347769
H	8.90353397315356	-9.53651709826056	3.77486863900826

$^{2\prime}$**TS3** (110*i* cm^{-1})

N	5.51221679876452	-10.19030425569655	0.18589637978336
H	6.13256195852559	-9.65217295104091	-0.39719079609663
C	4.35585324391028	-9.74070176802568	0.75541773087993
C	5.66095313703029	-11.48340439418406	0.52697569664662
H	6.48271901048530	-12.11089822515578	0.22766988950485
N	4.66057530630982	-11.87485760695470	1.28343622462626
C	3.83666140107650	-10.80147014269875	1.43638803078102
H	2.94102864318177	-10.86205399839673	2.02045347887694
N	0.76745431723725	-14.55539610469684	0.31810017634950
H	0.16962308276126	-14.64097771049756	-0.48844928225009
C	0.38897300593052	-14.73656589797504	1.61591148573995
C	2.07652067280954	-14.24429142560058	0.31812554131713
H	2.65896353450569	-14.05442362803137	-0.56092642951580
N	2.54957868338953	-14.22046966531450	1.54138272055537
C	1.50874185187169	-14.52535624150906	2.36459002256253
H	1.62643459347523	-14.56649059937224	3.42799698606037
C	7.46651322386758	-16.15391630125283	-2.04413619136260
H	8.28198561858612	-15.53998031456102	-1.66572160036824
H	7.37031890748990	-16.00329113200930	-3.12206760486839
N	6.26904230159315	-15.74820554397632	-1.35872565428975
H	5.41547111579539	-16.25156145093376	-1.53998379711310
C	6.21682271767667	-14.76534084082689	-0.40902904665459
O	5.07767971553193	-14.56497689492172	0.08783485951764
O	7.27039700335686	-14.10093191897873	-0.19419860314178

N	9.93700365733787	-15.34065528642026	0.75787713840010
C	10.76436826045335	-15.02968499331446	-0.28087943618210
C	10.11067064463099	-16.62040675390270	1.00097251335183
H	9.58729208103425	-17.16935528556103	1.75922958882085
N	11.02168627955966	-17.14521553161334	0.16282143019578
H	11.33501536208834	-18.10258318812391	0.14218892051472
C	11.44819828529993	-16.14598671592555	-0.66144217437285
H	12.17931944238334	-16.30156768440831	-1.42676539333771
N	10.55985037504219	-10.84750133879894	2.54704078463595
H	11.41882949001045	-10.33664151763625	2.67540063993674
C	9.31561063296397	-10.42745100764009	2.91458014069926
C	10.42817874777440	-12.07127117603013	2.00303963441631
H	11.25103003125589	-12.64716031315517	1.62869663988135
N	9.17225223527863	-12.45167399861519	2.00158803326117
C	8.46165460009724	-11.43283145227812	2.56999955476519
H	7.39780966986373	-11.50323162921128	2.69043898921769
C	3.30389069701635	-12.21367110215828	6.03065330508929
H	3.00323670712486	-12.79264507752439	6.89929951484894
H	3.92914659823538	-11.39416867916492	6.37814475073529
C	4.11130324228117	-13.07840112547630	5.09732757707977
O	3.89612913740641	-13.09911663874787	3.89845456198789
O	5.02292139828651	-13.77413340450524	5.71714868356330
Ni	8.29100041759542	-14.33527248197879	1.53725325463960
Ni	4.55701582986663	-13.82748843172658	1.98819849584570
O	6.54942028121413	-13.95127043085822	2.65807896798096
H	5.61936693005450	-14.34000207174637	5.13567453332102
C	6.52348910189782	-15.28838279771350	2.95423100411797
O	7.39541825447825	-15.99000136046042	2.25065791463100
O	7.99320132487170	-14.58985532377748	4.66743415092902
H	7.80961660094961	-13.77588476517927	4.15471523066193
N	6.81691629574633	-15.37041266096616	4.43905099824630
N	5.12381132995316	-15.78374913854859	2.80314941622459
H	7.09941762751991	-16.32163653220859	4.67368918846474
H	5.11974981266183	-16.46999250289157	2.05152100001180
H	4.77439370355735	-16.21631379792510	3.65394235643209
H	2.43588094008152	-11.81421579192331	5.51660519175720
H	4.00587435100858	-8.73689803734287	0.63490779752752
H	-0.61125489236188	-14.99435267997751	1.89453032280513
H	7.68431801493016	-17.20692657921990	-1.84936580182735
H	10.80541943335359	-14.03987584758840	-0.68864770927221
H	9.14272556203401	-9.47986179514477	3.37998682245372

$^{2\prime}$Int3

N	5.58364551902939	-10.22770237000737	0.05744465156364
H	6.23125081386569	-9.71582127302191	-0.51976501202505
C	4.39198472771156	-9.76144673296698	0.53225000163803
C	5.72555701832530	-11.49603151776754	0.48339632132324
H	6.57060589476592	-12.12599087000469	0.26675296652979
N	4.68779593085208	-11.85633590306887	1.20421709581455
C	3.84586011821180	-10.78720437595563	1.24494466610603
H	2.91726919275556	-10.82241597971121	1.77654685349995
N	0.81949835959800	-15.00253231172170	0.59431539102353
H	0.26185113438807	-15.54655305279711	-0.04468775833023
C	0.34239833711650	-14.12706811485518	1.52482439977158
C	2.16145630874385	-14.99986725566794	0.68760379651689
H	2.81618117886326	-15.58172608907116	0.07111009022930
N	2.56314633313180	-14.17318797230287	1.62371763904583
C	1.43858289444579	-13.61864289071221	2.15552652616621
H	1.49053987598218	-12.90336510409927	2.94982287451804
C	7.35786502861622	-15.93046557687888	-2.28187689111382
H	8.22908567528879	-15.50871677992648	-1.78375358523812
H	7.24861814617861	-15.46418403133469	-3.26506278524404
N	6.21536243859156	-15.67680743545191	-1.44400745532306
H	5.32235800589986	-16.04120564430267	-1.73470631096044
C	6.21215335071128	-14.78092316683161	-0.40614400292382
O	5.09901472881926	-14.61806176058505	0.15543328913449
O	7.29708678247663	-14.18841430965382	-0.15679210144473
N	9.91604101510457	-15.33709756169793	0.82048390208072
C	10.71095449692710	-15.07452290029135	-0.25630332780615
C	10.06005430290958	-16.61650002896447	1.08568892745346
H	9.55207991098561	-17.13099900753854	1.87807981040553
N	10.92168414590913	-17.18705270089936	0.22553211196345
H	11.20474214661649	-18.15398230166894	0.21450475725924
C	11.34550065964014	-16.21951932105318	-0.63709395203138
H	12.04166071199852	-16.41380797526474	-1.42580446841136
N	10.63913468421155	-10.84627900531994	2.56890322333549
H	11.51598772372544	-10.37620782465679	2.72836848072606
C	9.40615319986768	-10.38068693254052	2.91808831607752
C	10.46795716719919	-12.05219116529780	1.99713148384585
H	11.27377637982005	-12.65391675152350	1.62610868170058
N	9.19676240563451	-12.37840109964597	1.95923616739752
C	8.51804988535407	-11.34075974636546	2.53346752460723
H	7.45018755287719	-11.36540994477447	2.63273282890684
C	2.73537400969692	-12.46946097786261	5.90877772597063
H	1.90730468654513	-13.16078709875526	6.05383896785330
H	3.13669304545156	-12.21690760084413	6.88553999954889

C	3.79406828675897	-13.15273620527542	5.08077642749468
O	3.91019755374039	-12.93348924405439	3.88650242095290
O	4.52338842264396	-13.97880295699720	5.77338989978088
Ni	8.30092303147535	-14.28067310252258	1.59611220737818
Ni	4.58465110934252	-13.76131256611701	2.02316340487659
O	6.57531808798584	-13.83221007407899	2.71377529586968
H	5.29054460419438	-14.40809051976271	5.27200278466599
C	6.55045918614942	-15.13560913961854	3.12658277756263
O	7.50328281696090	-15.85957738024021	2.56087970046281
O	7.63771402988796	-14.11463628951535	4.98027520503588
H	7.49917668121677	-13.44948221889024	4.26796487529956
N	6.68183438111650	-15.11537867787727	4.63452789771075
N	5.18026156603849	-15.68603169546505	2.88450423559662
H	7.09876579118379	-15.99528943832417	4.93891098471106
H	5.25503849625828	-16.34457319593942	2.11121988446659
H	4.81639912975852	-16.17193403174937	3.69983831856140
H	2.37518646113981	-11.57987599454959	5.40205604926364
H	4.03854734982465	-8.77167881689596	0.33216854670052
H	-0.70246377733646	-13.94179410845259	1.66014943389995
H	7.49481332309836	-17.00531271150347	-2.41216334662729
H	10.76666570531867	-14.09527337121007	-0.68714789808775
H	9.26247173839843	-9.43654643729606	3.39992007326315

$^{2\prime}$**TS4** (143*i* cm^{-1})

N	5.55078613602057	-10.15373882370988	0.10393292974191
H	6.16184894247831	-9.64168272591956	-0.51170098459277
C	4.42188478776362	-9.66766232195321	0.69804466318280
C	5.68469678890196	-11.44117877971436	0.47319080603412
H	6.48973999921448	-12.08505809987760	0.16714773847044
N	4.70128570403378	-11.79355539000486	1.26986631095787
C	3.90412638766699	-10.70070673983416	1.42140950750326
H	3.02885355680856	-10.72820865456089	2.03705462793586
N	0.80926588591463	-14.87126919075224	0.55837432650369
H	0.23942445127227	-15.38754672663657	-0.09285194700574
C	0.34917083096858	-14.04657150058430	1.54301128987269
C	2.15305291744883	-14.86494964620888	0.62090551749172
H	2.79740493150015	-15.41195772312141	-0.03712709448284
N	2.57013827981399	-14.08301049787452	1.58827994851869
C	1.45617737673491	-13.56241974261385	2.17362278210139
H	1.52760343755000	-12.89027545326764	3.00335186083946
C	7.44443094087939	-15.98576049457619	-2.16338780024237
H	8.30903075529526	-15.56284176156651	-1.65485622627711
H	7.36477256797720	-15.54265180853847	-3.16014544871416

N	6.28529789228056	-15.69809286445941	-1.36052938079804
H	5.39570384337414	-16.05871404866155	-1.66693859173389
C	6.25770381703183	-14.76604778963818	-0.35302342349805
O	5.13648731361587	-14.56687570284093	0.16638867024580
O	7.34788131444994	-14.17529762356688	-0.09528141570462
N	9.93404360681154	-15.39001392823785	0.89064486678209
C	10.75299918097742	-15.15052423450769	-0.17409822029230
C	10.05637501966988	-16.66873296217606	1.17425311185711
H	9.52589452844325	-17.16829993657323	1.96168378330561
N	10.92682517629469	-17.25942285867344	0.33800014484869
H	11.19805920754709	-18.22993602889218	0.34410134733486
C	11.37961720709798	-16.30844754547218	-0.52786024383891
H	12.08796597446437	-16.52135244827970	-1.30082757016402
N	10.59233833845389	-10.72859243641195	2.31503842295076
H	11.44757536777323	-10.20052101041830	2.38864661261376
C	9.35000775150228	-10.29326200086106	2.66997779319717
C	10.46648190475854	-11.99147129948769	1.87037661187871
H	11.28995274529234	-12.58580366556797	1.52700672697852
N	9.21380234346200	-12.38371722253603	1.92056579521080
C	8.50085382864129	-11.33007441399145	2.42045010474632
H	7.43958001563889	-11.39617272954087	2.56405987775932
C	2.72084607059124	-12.72174406706751	5.92959172272984
H	1.82654604308751	-13.34039520814632	5.96665254819152
H	3.10325080499336	-12.61303263767506	6.94022174131377
C	3.76068596190860	-13.42890560743033	5.06618893206383
O	3.91405765435760	-12.99659326547022	3.90651243733443
O	4.36476689759796	-14.39317952083494	5.59530266520425
Ni	8.34787499692566	-14.28899683599765	1.64681351759875
Ni	4.57530634760212	-13.71061573236488	2.04485989579362
O	6.58963715341372	-13.82722918568097	2.71525051617575
H	5.65581732178973	-14.75864116870718	5.14866497530047
C	6.51553855830635	-15.11449248508923	3.08360916818569
O	7.46832863020563	-15.86831232567952	2.61870321027738
O	7.58364443888561	-13.99475498826835	4.99675196398116
H	7.43212052008853	-13.38107205202400	4.22979564263776
N	6.67990459784940	-15.02583163805287	4.72033672846100
N	5.15340487584049	-15.64827194058979	2.90468785749486
H	7.07279630177497	-15.89216427127533	5.09350570591904
H	5.19652813016711	-16.43844176496956	2.26749084545293
H	4.69148255640521	-15.92049483950053	3.76793968123239
H	2.47643554798323	-11.75206961387580	5.50742989977326
H	4.08758211150189	-8.66034475627290	0.56316585991194
H	-0.69343625045565	-13.87602307264028	1.71124731053659

H	7.56893791742432	-17.06520805024164	-2.26491966804050
H	10.82816734161091	-14.17815082339560	-0.61729161273527
H	9.17349108431996	-9.31479324663972	3.06457733368696

2′P

N	3.88901152602084	-8.93318825086746	1.27748918429899
H	4.12804469731695	-8.01864433369922	0.92868489280133
C	2.88678204941647	-9.21320546686995	2.15975180152215
C	4.49431117831651	-10.09429216663396	0.96866652240706
H	5.32027322806344	-10.19598448047148	0.29279277489154
N	3.92785879408545	-11.09385344927305	1.60318591478258
C	2.92263103071210	-10.56212182229963	2.35324394603524
H	2.30883531921850	-11.17603397431573	2.98135478975520
N	2.33280550921477	-15.51097814072413	-0.72383979103850
H	2.23981243391972	-16.32408122580579	-1.31155723408016
C	1.32332818152624	-14.67371410896694	-0.34957770579644
C	3.46593378359541	-15.05886509351162	-0.15763638896582
H	4.42491392086972	-15.52192704890692	-0.28255605506414
N	3.23074086760902	-13.98016397053332	0.55293897948080
C	1.89725545309238	-13.72515915388291	0.44393284390521
H	1.44309059185489	-12.89145147483011	0.93903149352133
C	6.65323024584697	-14.42514952876805	-3.07635848984042
H	7.21937374865934	-15.21788855270387	-2.59145686785634
H	7.33830958041903	-13.81047049363541	-3.66841982669454
N	5.99757366521424	-13.66320197201242	-2.04341839114974
H	5.37730351724366	-12.91988409279971	-2.32503361044331
C	6.44700283827993	-13.59971170806246	-0.74399581097766
O	5.90174762990408	-12.72734803022902	-0.02406656707213
O	7.36035145780957	-14.41032655384155	-0.42954312649456
N	9.59631604624187	-15.91222790834440	1.07709848971991
C	10.10164391143092	-16.05246045679879	-0.18272422804199
C	10.02755257629536	-16.94393959172800	1.76772540346037
H	9.80307673966191	-17.13901201805967	2.79728713123856
N	10.79575271598550	-17.74036759144063	1.00542354282269
H	11.23901210508544	-18.59866417935206	1.29267218438524
C	10.85141182218968	-17.18932481790984	-0.24049566369388
H	11.40177087463858	-17.63418542502455	-1.04285450605325
N	10.27142094709293	-11.21957695630503	0.81685120399241
H	11.08733642155274	-10.71723166757410	0.50498863791070
C	9.10515690930211	-10.66292259501732	1.25657744495943
C	10.12917598579596	-12.55532251641340	0.88260506508057
H	10.89368789263349	-13.25087625773464	0.59901439838208
N	8.94142326076128	-12.87301227525902	1.34154618958450

C	8.28559350707594	-11.70277472495181	1.58188892335406
H	7.27771703445653	-11.69038218758706	1.95203949251820
C	4.97598701345142	-13.27315987757450	5.68441595215447
H	4.06432076104701	-13.70071503784439	6.09007195677438
H	5.81697384527678	-13.94110860888055	5.86720808406584
C	4.83539829976881	-13.05972368534824	4.19855996623073
O	3.81468202707045	-13.46088895846678	3.59413741308006
O	5.77128805333406	-12.49125124534275	3.56723551425363
Ni	7.98807362380594	-14.71178301122634	1.53825916023556
Ni	4.70168142445537	-13.01044006539498	1.68585808060744
O	5.84419595027154	-14.70864510888727	1.89821456607182
H	7.62242542935214	-15.78877537481301	4.38917733010639
C	5.88802122109480	-15.86022322762343	2.42372002706869
O	6.97863739097838	-16.48380195081566	2.44430973962219
N	7.87015573333033	-14.88184176975518	4.79131064338714
N	4.75029488338054	-16.39646202628389	2.92503342320516
H	8.79187130492519	-14.97047448871654	5.21706144229156
O	8.05531080370647	-13.98209559906985	3.68742842336417
H	7.26337101325604	-13.37536337862709	3.69861055103819
H	4.80073070709952	-17.23689886303029	3.47388343262377
H	3.98200949840406	-15.75952587393553	3.08006645455623
H	5.18933850777618	-12.32211551832182	6.16612623953399
H	2.24867995204147	-8.45734744607075	2.56701969251615
H	0.31071021426772	-14.81401173942507	-0.66480930555069
H	5.90813296717703	-14.86130023705905	-3.74265914590847
H	9.87684410919842	-15.34014756479775	-0.95059752722697
H	8.96020311712088	-9.60417287954422	1.30744728835138

The Cartesian coordinates for stationary points concerning the NBPTO inhibition process optimized at the UB3LYP-D3BJ/def2-SVP level

^{3}RS$_{QM}$

N	5.86771363993451	-9.82095418902344	1.43475993818473
H	6.22152260057460	-8.89522541466338	1.64332261811776
C	5.46921862240574	-10.28709024390307	0.19585832081789
C	5.67030150132293	-10.80627743528644	2.34654090605879
H	5.90570800940698	-10.74493429822122	3.41100103592659
N	5.16738586054486	-11.87393274694486	1.75102956786954
C	5.03703858893561	-11.57283635198991	0.41390261270261
H	4.65182601412941	-12.30072126906914	-0.29391825709600
N	0.88900336418868	-13.69570773769492	0.95771359176144
H	0.24521800216038	-13.82685458926779	0.18629567933303
C	0.57123791924121	-13.19388985680609	2.20463558380513
C	2.21476405340904	-13.98344091525322	0.94418928926737

H	2.76024688967409	-14.39493004069072	0.09960787692506
N	2.75508591863543	-13.69179627953972	2.11382083432762
C	1.74754044383177	-13.19783348033529	2.91429009591563
H	1.96223958200263	-12.88948189783856	3.93328436724696
C	6.80281258435332	-15.56688019495825	-2.28378209475114
H	7.77099788861501	-15.68467114809714	-1.78015256997638
H	6.89511648375420	-14.73726789340729	-3.00949024772670
N	5.78687959381164	-15.32747906421062	-1.28484732045669
H	4.82224482946668	-15.54714761219551	-1.49353587528018
C	6.03412175035310	-14.82273741395309	-0.03561434197159
O	5.04012011452416	-14.68965788793168	0.73828888516610
O	7.24050690402626	-14.51954039997412	0.22733565990362
N	9.65254841663756	-15.53998471862177	1.61094653829926
C	10.92470283316674	-15.09042680618825	1.33405960289421
C	9.64475720766450	-16.84636202805930	1.40325377027374
H	8.77645025967224	-17.48529318695103	1.54590004658468
N	10.87199119153026	-17.25529338122914	1.00244774259139
H	11.13369455306636	-18.20964349665378	0.78343347056481
C	11.70395442325470	-16.15475298204383	0.95277371315929
H	12.74820484810597	-16.22471701774045	0.66231554744981
N	9.19937584635045	-10.73352634602050	0.67849793924682
H	9.28640751058349	-10.08285570336227	-0.09323570279347
C	9.48842268217534	-10.46962476261395	2.00386489388251
C	8.78818615797305	-12.02172872758700	0.58040913302644
H	8.45815599853692	-12.51201850947319	-0.33248225478083
N	8.80790586080156	-12.59115387807401	1.77216179922954
C	9.23351858781390	-11.64134983801861	2.67437331888218
H	9.31330152116198	-11.85291915253079	3.73798718610255
C	4.14580664959340	-12.12475565632425	6.65236737571920
H	3.54307559225028	-12.97979189578364	6.98851420821616
H	4.87912508305013	-11.84255365340577	7.41918623080160
C	4.83786468575901	-12.42610870408826	5.33058044591676
O	4.19316924000532	-13.17373849364541	4.52156267470027
O	5.95970740172020	-11.91686941781528	5.11177024899101
Ni	7.95577008542351	-14.47674796765218	2.09899361128045
Ni	4.80180825662180	-13.73638507526445	2.62353621641975
O	6.69964763698992	-13.78120236611338	3.36643680646697
H	6.71027570036303	-12.93940185820119	3.87225405931504
O	7.03705760786831	-16.42995940578448	2.60510661673764
N	6.46298577544187	-15.83936372605872	5.10965706193177
H	6.72086701789774	-14.87899639331634	4.81998986486340
N	4.51837523325058	-15.86341495344053	3.25104708433411
H	4.09945961666950	-16.24727344798589	2.40399539345388

H	3.83272877993416	-15.75076304255056	3.99818080912790
P	6.00531612877808	-16.57410625997368	3.70225368127483
C	5.66701556195331	-15.89139086175764	6.33432173732065
H	5.56285398246171	-16.94275233467792	6.65380205756910
H	4.64414147517090	-15.49165173034563	6.17861134853966
C	6.33851188050860	-15.07254259363743	7.43182488205883
H	7.35126770457105	-15.47505086266277	7.60530719841296
H	6.47496609864474	-14.04102167899300	7.06197499723617
C	5.54551283099152	-15.04954929741895	8.73790173088069
H	5.44336839974890	-16.07998990634599	9.12331221788232
H	4.51830401033062	-14.69947509230624	8.53221415399136
C	6.18288101128117	-14.16012330499090	9.80350002256092
H	5.59858428401899	-14.16379254237896	10.73643389648207
H	7.20389522638971	-14.49722950343120	10.04638460923295
H	6.25390516760473	-13.11640658846237	9.45568199284790
N	5.64749949906251	-18.18679942978124	3.95988608334025
H	6.43956468779616	-18.78501965276009	4.18403068641759
H	4.82920286817159	-18.41223200389483	4.51864784757046
H	3.46089749902036	-11.27304753640535	6.50527887353484
H	6.57968078033432	-16.48747688057281	-2.84528547530435
H	11.19292651900927	-14.04182558844493	1.42905804647300
H	9.84812803485905	-9.50236919437594	2.34318262007714
H	-0.43681192480253	-12.88710663674142	2.46876442034032
H	5.51538289345762	-9.67591323578364	-0.70104373770045

$^{3}TS_{QM}$

N	6.07527991766041	-9.87503918163800	1.75207018274445
H	6.45228024612927	-9.01492150862445	2.13116757160508
C	5.73659453205083	-10.10834262123389	0.43081319526676
C	5.77186557445853	-10.97956597222181	2.47807484853032
H	5.91766463765857	-11.12013764855037	3.55395964384807
N	5.25654515174755	-11.89986081692052	1.68088042174072
C	5.22665103648060	-11.38322607337846	0.40547187667125
H	4.84489426444975	-11.96147109483212	-0.43156278205109
N	0.76710603957610	-13.61563639944985	0.41215537918578
H	0.14093335376326	-13.85897133292295	-0.34639033159624
C	0.43565108890239	-12.88489931684558	1.53709254818438
C	2.07181249244312	-13.96848061302044	0.50517000120261
H	2.62150689612225	-14.55515337040607	-0.22513956671582
N	2.58703492647438	-13.50003409985565	1.62876773455541
C	1.58357272219548	-12.82360151577952	2.28753626432765
H	1.76831334238092	-12.37060200899482	3.25636484901068
C	6.83347376998544	-14.96735437856371	-2.58462230359620

H	7.73706022674013	-15.31311589941719	-2.06575730242526
H	7.07095353486789	-14.00662554819577	-3.07835643705344
N	5.75722323186768	-14.85247669070280	-1.62689540342090
H	4.80079212930383	-14.95774809564690	-1.93749415169884
C	5.93574323352189	-14.66452725425150	-0.28319354444159
O	4.89512881529698	-14.64036857703544	0.43792675032645
O	7.13462980034073	-14.51976674203789	0.11650929187756
N	9.51215397799283	-15.73354168525059	1.38005595340203
C	10.66965822651189	-15.21743648378627	0.84278870468641
C	9.61465054890323	-17.05191131840304	1.33448192331335
H	8.84888248657533	-17.73849902062781	1.69017267746562
N	10.80263968473834	-17.40315019084578	0.78739385352926
H	11.12657130584691	-18.35227508300490	0.63985219791941
C	11.49212513798790	-16.25096124847344	0.46649024193631
H	12.47954801222597	-16.26828547193259	0.01423362185291
N	9.20925771962758	-10.96668386291799	1.41266140604047
H	9.43998841672907	-10.20343400830231	0.78758618096807
C	9.34588780332660	-10.96485453759997	2.78759647774356
C	8.71120872716434	-12.17036689063876	1.03557849918861
H	8.46387424125413	-12.46056384167400	0.01705107948039
N	8.53324889687751	-12.93484565379838	2.09810006596599
C	8.91402880005468	-12.20226528776237	3.19947799182572
H	8.83007418851160	-12.59415617978224	4.20991832788725
C	3.52998025342590	-12.60619379937735	6.45070717905484
H	3.11583311581410	-13.59346972882521	6.70625317279245
H	4.11373179290010	-12.20974292691097	7.29089475968233
C	4.37561719120420	-12.68936598879388	5.18440694702573
O	3.79161243358367	-13.26930787889920	4.19274249152407
O	5.51819381356932	-12.21181231275813	5.17207202193214
Ni	7.74463877918842	-14.83977989814895	1.97444419027141
Ni	4.56374007885042	-13.72364730555588	2.28967450692805
O	6.20619474084943	-14.47604456125993	3.20397183973523
H	6.59187042205087	-14.36189387904899	4.42662958133032
O	7.13324752135455	-16.62755014063114	2.72961999743007
N	6.80540212571587	-15.27602269606836	5.23585443055742
H	7.79995290249869	-15.50673812484841	5.30776259601118
N	4.40446917976327	-16.03986977310056	3.78721482132339
H	3.84652491319838	-16.85973836733399	3.99985335977472
H	3.99220904756631	-15.13550780894441	4.04548109084194
P	6.07107157346862	-16.23578612202117	3.80629722291111
C	6.16729229244593	-15.31260253653584	6.55719827355174
H	6.26254034551734	-16.32553407794889	6.98737126001024
H	5.09364952899627	-15.12737249581486	6.40645187483611

C	6.75468471231593	-14.26373721444685	7.49482345805785
H	7.83746372187947	-14.45418818637755	7.61910197435542
H	6.65153794082610	-13.27922919781427	7.01030879333879
C	6.08019959884763	-14.25038692536818	8.86721515346249
H	6.18843343732891	-15.24076180927284	9.34496604624946
H	4.99547053459475	-14.09572457141393	8.73210870929532
C	6.63935374951764	-13.16795373495494	9.78841889086640
H	6.13834692942545	-13.17266914381489	10.76846368236116
H	7.71831014602800	-13.31112715638453	9.96426360843608
H	6.50628506975934	-12.16674121843082	9.34681914244412
N	5.98116942147329	-17.79689206873991	4.51877545268784
H	6.81030253496684	-18.37456945464141	4.43686929981928
H	5.49498701410565	-17.92571184430948	5.40089199670121
H	2.66950674763515	-11.94274323824858	6.26750241250081
H	6.57119332503746	-15.69670670230304	-3.36619698906332
H	10.83722931698391	-14.14682168011500	0.76513656884873
H	9.72988485711194	-10.10992170056813	3.33675315481808
H	-0.56211705415871	-12.48802427488170	1.70051198035990
H	5.86990316561287	-9.36229871975972	-0.34750623234772

$^3PS_{QM}$

N	5.78619247070265	-10.15472466606581	2.51116426956718
H	6.05344172286595	-9.37948529743712	3.10560610244711
C	5.68597454923710	-10.13142811280234	1.13111692073436
C	5.41044468260931	-11.38115522981252	2.95212474527133
H	5.38103299110468	-11.73605491243607	3.99055997002500
N	5.07835046157835	-12.13681497945870	1.91898962903189
C	5.24259938420573	-11.38209805759851	0.77812599507397
H	5.03245615951932	-11.78859742535322	-0.20722253684910
N	0.80533618876251	-13.17503424208129	-0.11979092359756
H	0.27558524830969	-13.12409819542889	-0.98206325843929
C	0.34847885578201	-12.82511142958812	1.13592595218017
C	2.08437610884693	-13.60862123267060	0.00166366723946
H	2.70989989258702	-13.95694565116709	-0.81498128204357
N	2.46188360096490	-13.55245485470374	1.26608261804405
C	1.39557719924497	-13.06809512679445	1.99083394393933
H	1.47136383988315	-12.95054650681883	3.06768780178728
C	7.06529130234943	-14.22748932289539	-2.78987041742170
H	7.90439943896266	-14.74026877485899	-2.30260244357607
H	7.39143186712894	-13.20270148895329	-3.04597186211687
N	5.92787857199890	-14.24324883656669	-1.89676913890704
H	4.99509207286199	-14.17047564047077	-2.28020958679818
C	6.00560448393682	-14.42727663731955	-0.54318272543962

O	4.91717242971618	-14.48626726496314	0.09816712763081
O	7.17894215606107	-14.51490894544766	-0.05275715939874
N	9.30292676043806	-16.18753240300721	1.27273664247274
C	10.58338849496949	-15.74380253956047	1.02659946480971
C	9.29411312628643	-17.49776333584054	1.07553229665140
H	8.41571237674447	-18.12976135294212	1.19431533818841
N	10.52889777785359	-17.91166496329784	0.71160868711363
H	10.79160153091050	-18.86913010691885	0.50603130455283
C	11.36638555932880	-16.81455643447059	0.67354508257882
H	12.41709216982145	-16.89078579784962	0.40911077856600
N	9.04705459754119	-11.30596131821969	2.11849696070911
H	9.32217901893387	-10.43074387428953	1.68862814773350
C	8.93727658096457	-11.55071057662991	3.47395940044913
C	8.70824255839513	-12.43531878091067	1.45263505022164
H	8.65768360991425	-12.53644515225094	0.37123997166938
N	8.39858998498737	-13.38802161088383	2.31558706883343
C	8.52435932811915	-12.85524574389064	3.58009064566333
H	8.27299684220160	-13.42023863938899	4.47235885463648
C	3.22940520608015	-14.46642515273155	6.19765742250320
H	3.55080462392145	-15.52093340403512	6.22226471547686
H	3.48166935146414	-13.99334067722212	7.15561499350776
C	3.95689483252011	-13.79222103367422	5.04302317606108
O	3.44779974311675	-14.00926280266380	3.88919539749191
O	4.98752202779900	-13.13161815301860	5.27206797550598
Ni	7.60596069535905	-15.18352328429667	1.74354771650798
Ni	4.30982061187093	-14.02571075469691	2.06298605978446
O	6.01455137251062	-14.93633694688073	2.99507060624397
H	6.44896026019466	-14.52574440144518	5.45853965722532
O	6.49835242442281	-17.08440530339295	1.85607785269581
N	6.90670335312030	-15.29520096778782	5.96463027339964
H	7.89061377168620	-15.30488576688818	5.69244290676798
N	3.98031934447915	-16.30887976192127	2.18353339987437
H	3.86403875503687	-16.69540758702025	1.25017960918509
H	3.19545892481975	-16.46414915715804	2.81186377157029
P	5.53723418956867	-16.43062234604063	2.85730187010952
C	6.78003170847912	-15.05978410868575	7.40513149015176
H	7.32107851688776	-15.85745667586771	7.94341390362545
H	5.71680408459839	-15.17617506594304	7.67122697370709
C	7.26272582839253	-13.68465005540438	7.86717541429202
H	8.32683280919080	-13.56135632406763	7.58524814845437
H	6.69247572825623	-12.92379327268330	7.30666230830701
C	7.10479355870243	-13.45455851477282	9.37022118225657
H	7.65380992128138	-14.23923412594596	9.92171101783909

H	6.04202298276072	-13.58253157476882	9.64378307800808
C	7.58673660576456	-12.07750820411209	9.82350179587277
H	7.45329276012269	-11.93617125168834	10.90730177487308
H	8.65718275052308	-11.93560996884132	9.59843778817847
H	7.03194903932689	-11.27416416256778	9.31083949010974
N	5.37143870957553	-17.04400793387676	4.37060070985569
H	5.96603901260838	-16.54882896302494	5.08980798762404
H	5.29849845996806	-18.05191539263478	4.47672461211781
H	2.14181831309006	-14.45828555125136	6.03840392639778
H	6.82474471578573	-14.75362582730570	-3.72661090188274
H	10.85551132939936	-14.69626034551171	1.12270582843218
H	9.15144151728579	-10.79616071777255	4.22518965547323
H	-0.65598686520956	-12.44805164386096	1.30523433010103
H	5.91565221860725	-9.24766061648922	0.54256877905955

The Cartesian coordinates for stationary points concerning the NBPTO inhibition process optimized at the GFN2-xTB level

^{3}**RS**$_{SQM}$

N	6.02978580748889	-9.88466089954230	1.20074517265009
H	6.54557547446232	-9.02637443160844	1.30811129769942
C	5.35420830772474	-10.28277885698457	0.08357631808075
C	5.84232049419223	-10.82760723527370	2.14366537257063
H	6.26174233785484	-10.76916069427109	3.12982676472148
N	5.08667303943311	-11.79832065425208	1.68721865815852
C	4.77382928253737	-11.47362156798886	0.40226027235492
H	4.16546139840872	-12.11487868183655	-0.20190094339410
N	0.70779028824197	-13.81906582770057	1.35029897860177
H	-0.00927041908879	-14.13446019907188	0.71653381712037
C	0.52581401515745	-12.97536302014253	2.40729649460196
C	2.01240900229705	-14.14832849747057	1.31783550993403
H	2.45712555811822	-14.79787193342289	0.59106998324885
N	2.66762781916012	-13.56134499832254	2.29179826069292
C	1.75254057094292	-12.82409523057465	2.98143448638754
H	2.04363315873218	-12.25002788943570	3.83649333684642
C	6.69971971893063	-16.09608466879793	-1.89616736080359
H	7.66339245955291	-15.77307422781257	-1.50620281063122
H	6.52255195477698	-15.60949980716112	-2.85966831004785
N	5.70172177285358	-15.73053461255055	-0.92584477250620
H	4.74921856696656	-16.01114583636440	-1.09365743107813
C	5.92446820834539	-14.86443225728289	0.11627747384936

O	4.92253781682159	-14.60102887172961	0.81996273569308
O	7.09597606740128	-14.40498050141311	0.22631597156574
N	9.71538180771629	-15.60981997566498	1.08996018075625
C	10.99323657422028	-15.35422701386091	0.69024760389003
C	9.47833753288685	-16.87031872169526	0.79688785026827
H	8.55439608356571	-17.37721820104976	1.00074254892901
N	10.55542466301951	-17.43504187486775	0.22460497575023
H	10.63762383090779	-18.39005633037728	-0.08637337965536
C	11.53016976108765	-16.48472163554376	0.14952527160564
H	12.49633526788684	-16.68005995407672	-0.26622298438746
N	9.71439651946843	-10.74608314565878	0.83066331282206
H	10.04803296866837	-10.10723508693988	0.12649760811668
C	9.53214325863271	-10.46019940841994	2.15311611556051
C	9.39768447192472	-12.04132860059300	0.65005104614693
H	9.43119180234279	-12.54810613346919	-0.29358173377493
N	9.02540626193854	-12.59196270396258	1.78200765041849
C	9.10009982674182	-11.61612238300591	2.73309371186728
H	8.84278946253152	-11.79788449728682	3.75840461221976
C	4.52097351955095	-11.40337666204300	6.41883717220939
H	3.66742654094525	-11.98016385144635	6.76346749649644
H	5.25625337035527	-11.28998418751880	7.21052008289591
C	5.15702835834376	-12.08009820453852	5.20555166249592
O	4.36834290241815	-12.66173340968262	4.42140769323290
O	6.39443190047865	-12.00763872773938	5.06621596543495
S	8.15780538377595	-14.48670011386626	1.94178213605755
S	4.71513335326998	-13.60785336095005	2.61707632186860
O	6.65587342319275	-13.81840933159196	3.16188425603390
H	6.74840348720303	-13.06607143398037	3.79366877035614
O	7.34360908754763	-16.13581895050520	2.81508581094009
N	7.05250959012418	-15.00030263887496	5.32890808788464
H	7.03642071326016	-13.98946854068224	5.35032851564006
N	4.75172145218764	-15.43678938210762	3.55842413486343
H	4.49297821784906	-16.17280745959276	2.90585748859752
H	4.17905331675700	-15.51873199063617	4.39363834537723
P	6.43285331127545	-15.72084410469366	3.94090469544042
C	6.93602704609953	-15.52272351445647	6.67361429141390
H	7.73268021637756	-15.06100236371831	7.26617369156567
H	7.11970197510029	-16.59914993356862	6.66708234184427
C	5.58502972538843	-15.21296297020345	7.32317802854934
H	5.41758370011475	-14.13447115365919	7.28478017290734
H	4.78489669665189	-15.68834232052275	6.74719746880406
C	5.51859962809345	-15.68747357196884	8.77228612554099
H	6.32080066000636	-15.21349740083922	9.34125842689894

H	5.68166838074612	-16.76725274694103	8.80935006201852
C	4.17723685049196	-15.35310573315514	9.41331802887895
H	4.15215927644580	-15.69611814561214	10.44425040515704
H	4.00844999099460	-14.27867338071962	9.40550128537445
H	3.36280669622021	-15.83200020065182	8.87359658109794
N	6.08902734994002	-17.31257879034232	4.51408335251557
H	6.90279169280543	-17.90573302167953	4.50283752357208
H	5.59717430921406	-17.41698097014106	5.38771133435517
H	4.17351937718983	-10.41708122467051	6.11768632926720
H	6.70372172122657	-17.17770491998971	-2.04256787677854
H	11.44450297186182	-14.39175463104106	0.82113145040791
H	9.72816580048205	-9.49299130846765	2.56656761059987
H	-0.42980440240488	-12.56479542371259	2.65755899764005
H	5.33808765356702	-9.70482452600552	-0.81659243430592

$^3TS_{SQM}$

N	6.02361774957662	-9.83890536852777	1.88900538464580
H	6.48793383118356	-9.02831627088611	2.26511478814024
C	5.53263421193518	-9.96918515583763	0.62090935079593
C	5.72215012510341	-10.96716237014585	2.55942579596654
H	5.98838877021438	-11.16015104857656	3.58264918532800
N	5.06845528415012	-11.80033496605797	1.78594402792760
C	4.94274091532725	-11.19655066306090	0.57203367214954
H	4.44721585167749	-11.68111925516795	-0.24429847656015
N	0.67052517040453	-13.87154596395152	0.56433229065355
H	0.03731592096518	-14.18368657062862	-0.15407061095275
C	0.35261247412260	-13.05536637476627	1.61109799783029
C	1.97701204406442	-14.17646333129209	0.67317372735672
H	2.51834503265692	-14.79801741308544	-0.01090050279904
N	2.50363541826746	-13.59845300675459	1.72573115186341
C	1.50343614024170	-12.89353216621801	2.32306489405626
H	1.68314729700715	-12.33646196231074	3.21899180448843
C	7.03068850561978	-15.71969464650371	-2.22827235072633
H	7.93801732140682	-15.54427899142155	-1.65298255433030
H	7.05588970088411	-15.10558771224994	-3.13288084097033
N	5.91604728668833	-15.37206028085628	-1.38736908307921
H	4.98512010714329	-15.54959680753201	-1.72698167609064
C	6.02950533720706	-14.73485364062596	-0.17878140961387
O	4.95337842153809	-14.49254731685404	0.40853104118536
O	7.20215278321338	-14.42801093595492	0.18350275196055
N	9.66155190892763	-15.66542738214811	1.30730621567410
C	10.90238491980842	-15.27249591228298	0.90090705268340
C	9.51633566725852	-16.91025197951884	0.90499271256845

H	8.64148844215817	-17.50712769104219	1.08066039356445
N	10.61769252778752	-17.33325365952520	0.26110616250934
H	10.76387339762246	-18.24687399778725	-0.13757042987273
C	11.51111497141566	-16.30383050364963	0.24965254838761
H	12.47766359996264	-16.38286180973452	-0.20280061212074
N	9.43771176395968	-10.79430458058417	1.57195365838648
H	9.79153161461925	-10.05575488386807	0.98481847113078
C	9.17322974693627	-10.70252019440049	2.90832835517590
C	9.16471343104659	-12.05544664685235	1.19218114076622
H	9.26331219819140	-12.42479532431345	0.19077455164077
N	8.74556900892108	-12.76833135582095	2.21235931636337
C	8.73927086823113	-11.93647927802097	3.29358414860871
H	8.40668044760241	-12.26081629814678	4.25998742371464
C	3.67836289122353	-12.11719332674264	6.41222415749493
H	2.87105240855176	-12.82084757063046	6.59294699227879
H	4.30814223175175	-12.02136692795530	7.29160289827649
C	4.51853127213378	-12.57099801693670	5.21072598173815
O	3.85508063136716	-13.08146938397974	4.26762588439948
O	5.74302364285874	-12.40896640209830	5.24357745081223
Ni	7.95032412090277	-14.68343030195483	2.03950605630023
Ni	4.51846670050775	-13.72684490748478	2.34342293918601
O	6.33039570478918	-14.22829865578759	3.22697695548873
H	6.53450822388338	-14.10622187429472	4.50806190678959
O	7.12064882765011	-16.46384506801762	2.68911228985179
N	6.74867230168082	-15.13142196442954	5.26109563013236
H	7.75548378615649	-15.27671075076612	5.30995188627806
N	4.41681156705946	-15.73850676333652	3.16771966978199
H	4.03219186159167	-16.62456160683172	2.85764573586583
H	3.88758805245604	-15.41236276151744	3.97538973185835
P	6.09305346683316	-15.91420927352328	3.64281207859326
C	6.15440978684233	-15.27867046287899	6.57795320489976
H	6.26507360183713	-16.30718763485505	6.95045881056847
H	5.08634416597466	-15.05960321868510	6.48935692536532
C	6.79023654743815	-14.30304631906492	7.56698574679504
H	7.85385085706414	-14.53563125537729	7.67231868732957
H	6.70075857220620	-13.29657374802693	7.15427248690450
C	6.12157687786911	-14.36490169545235	8.93772338459660
H	6.21558838806288	-15.37437894708494	9.34527505740391
H	5.05558763746909	-14.15015382888832	8.82922382110238
C	6.74339875022124	-13.36704343548606	9.90657172399204
H	6.24990085983857	-13.41525331220927	10.87384067627977
H	7.80083041622608	-13.58192598647208	10.05131755855879
H	6.65006936948648	-12.35414933571818	9.52194358621046

N	5.71270943346421	-17.42859876999788	4.43174942940084
H	6.52366973205132	-17.98919494736673	4.63655805861167
H	5.08923248908229	-17.42007521500248	5.22365805125135
H	3.24453562383232	-11.14781183285901	6.17827163780094
H	6.97562121855881	-16.77134593397701	-2.51680661248386
H	11.27929606249089	-14.29223582174148	1.10907686669520
H	9.32211313143808	-9.80091094614788	3.46512698767029
H	-0.63373739796761	-12.66881818200587	1.75965525357445
H	5.63400476006832	-9.20166713745247	-0.11779088606007

$^{3}PS_{SQM}$

N	4.24643726329483	-9.75016704281258	2.73564325424833
H	3.95729220042815	-8.91262639792327	3.21510663288295
C	5.46584855178912	-9.97108091371095	2.16428039200285
C	3.51828076722386	-10.86696748613909	2.58073187662549
H	2.51258314721038	-10.98330086004716	2.93211994712626
N	4.20536887847248	-11.78214460073787	1.93513186714840
C	5.42780929947101	-11.24105747199515	1.66988822239866
H	6.18998731273031	-11.80103015960843	1.16352654575077
N	0.84691198662944	-13.33724247207636	-1.20179327491446
H	0.58149928253990	-13.15675149264822	-2.15703048062410
C	-0.00647402625578	-13.62693942774472	-0.17687059904459
C	2.09511142500315	-13.33221848887963	-0.69959911490163
H	2.98110585972577	-13.13762774226041	-1.26925992698902
N	2.08041558916628	-13.60471296364296	0.58368909365380
C	0.77533486136033	-13.79165312480895	0.92756387107299
H	0.50123868700537	-14.01854357106879	1.93754536586781
C	7.88161488382522	-15.05662126343590	-1.70954221727771
H	8.53177628030132	-14.92503389141145	-0.84613433706706
H	8.13567769661439	-14.30596381850638	-2.46341104094756
N	6.52366247830025	-14.91362746166750	-1.25290532313367
H	5.78432867235576	-14.99734950345792	-1.93276939614842
C	6.17870085688695	-14.35385094174848	-0.04476803136588
O	4.95510514013457	-14.14920313561431	0.12204031319750
O	7.12925708472072	-14.07901114210246	0.74171620835889
N	9.01012272143610	-15.78455966744544	2.49275697882490
C	10.25948180569645	-15.74735143854030	3.03924155142919
C	9.02745705405834	-16.75091038225136	1.59832915614119
H	8.18235180047765	-17.04346076326225	1.00469981563995
N	10.23599057683993	-17.33455172154115	1.55213996292295
H	10.50705794637423	-18.10285585207540	0.95897531956187
C	11.03374037517033	-16.70950575131880	2.46325622820160
H	12.05352482844286	-16.98773069628344	2.62747975117792

N	8.85378457891841	-11.10384041787781	4.05770924249964
H	9.58947498244978	-10.41982425201794	4.13772796305828
C	7.60213005540317	-11.00578420194468	4.59288634156427
C	8.92385449151109	-12.27929348088219	3.41129636445623
H	9.79121440091852	-12.62292435521024	2.88354794569880
N	7.78906608017352	-12.93628148100069	3.51492039020953
C	6.94410849072491	-12.14846995668292	4.24668738165278
H	5.92291749035882	-12.42820029861084	4.44614503881179
C	2.14714006077023	-13.98741930879605	5.80890570581996
H	2.46108107091878	-15.02238835578316	5.92182523735998
H	2.37591586200197	-13.43117696146947	6.71259217248272
C	2.91715015692000	-13.38992756743498	4.62082124461271
O	2.49889030551454	-13.78596439225724	3.49229434338149
O	3.83374632183232	-12.60205905590486	4.83209828916947
Ni	7.20235966170581	-14.74472055428419	2.66882837826292
Ni	3.74044255395909	-13.79099788211522	1.83605604632691
O	5.21152659635029	-14.44172796334369	3.16095247569753
H	6.19330328294368	-14.53618624625521	5.63053425722263
O	6.12304619434604	-16.58922479610771	2.28289046590732
N	6.72565703738241	-15.40229083491245	5.60915248603982
H	7.14871014392285	-15.49227298556881	4.67523688901132
N	3.56249357483801	-16.02559440892164	1.88326060365964
H	3.71488285497948	-16.55875930663461	1.03789449469138
H	2.72392697046998	-16.30753121118568	2.37539587002238
P	4.92429064173935	-15.91455841868564	2.89954473180879
C	7.75804386436277	-15.38825944457385	6.62940763972662
H	8.31240294129586	-16.32832102240509	6.55549816011607
H	7.25839742179899	-15.36465523402060	7.60189168645201
C	8.72646732391412	-14.20701254009607	6.51868346517160
H	9.13536082915625	-14.19478277036972	5.50333666180444
H	8.16896544799754	-13.27766941079213	6.66377400511893
C	9.86786094001434	-14.27803074179888	7.52822747401961
H	10.42147324459911	-15.20845671685286	7.38547482282195
H	9.45659470507416	-14.28965225489187	8.53894280367154
C	10.81695698030774	-13.09425459097340	7.38190684835762
H	11.62200942938067	-13.15860613341798	8.10891456224776
H	11.25611850717680	-13.07494970417063	6.38584852159952
H	10.28566370662881	-12.15850781989959	7.54227486850234
N	4.36172969357545	-16.63375776934553	4.30915551916138
H	4.92603771527700	-16.34174923843470	5.10282890532166
H	4.38780998299098	-17.64132827367240	4.24266118070822
H	1.08070700716210	-13.96393000007507	5.60544570682703
H	8.02977040612765	-16.04960881154514	-2.13824687400760

H	10.52373368007490	-15.05022449900098	3.80790066262256
H	7.28735163558260	-10.16150706435895	5.16913561576647
H	-1.06577082248601	-13.69233162191757	-0.31158019354716
H	6.23490940150342	-9.22737725672706	2.14791478628799
